# Dose–Response Activity-Based DNA-Encoded Library Screening

**DOI:** 10.1021/acsmedchemlett.3c00159

**Published:** 2023-08-21

**Authors:** Patrick R. Fitzgerald, Wesley G. Cochrane, Brian M. Paegel

**Affiliations:** † Skaggs Doctoral Program in the Chemical and Biological Sciences, Scripps Research, La Jolla, California 92037, United States; ‡ Department of Pharmaceutical Sciences, University of California, Irvine, California 92697, United States; ¶ Departments of Chemistry & Biomedical Engineering, University of California, Irvine, California 92697, United States

**Keywords:** microfluidic, miniaturization, assay development, protease inhibition, phosphodiesterase inhibition, combinatorial chemistry

## Abstract

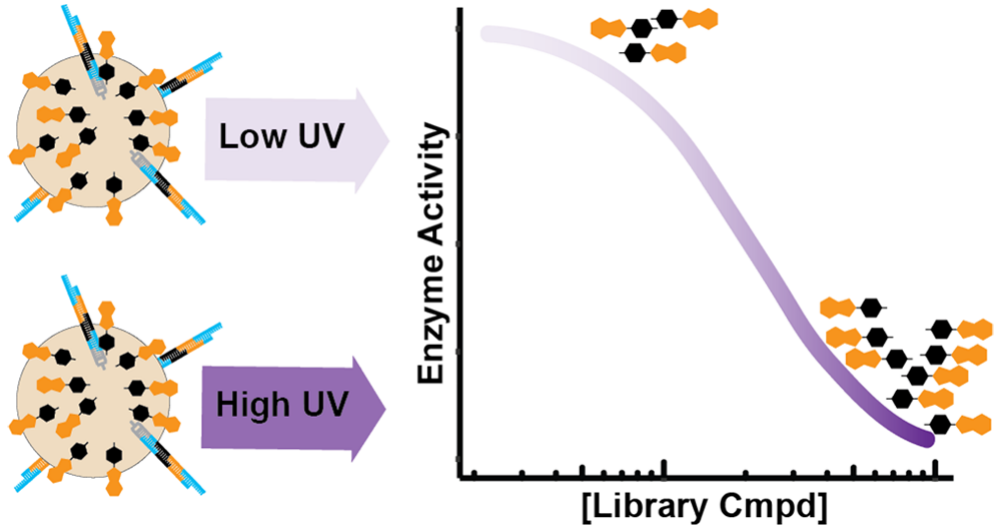

Dose–response, or “conforming” behavior, increases confidence in a screening hit’s authenticity. Here, we demonstrate dose–response solid-phase DNA-encoded library (DEL) screening. Compound dose in microfluidic droplets is modulated via the UV intensity of photocleavage from DEL beads. A 55,296-member DEL was screened at different UV intensities against model enzyme drug targets factor Xa (FXa) and autotaxin (ATX). Both screens yielded photochemical dose-dependent hit rates (FXa hit rates of 0.08/0.05% at 100/30% UV exposure; ATX hit rates of 0.24/0.08% at 100/20% UV exposure). FXa hits contained structures reflective of FXa inhibitors and four hits inhibited FXa (IC_50_ = 4.2 ± 0.1, 7.4 ± 0.3, 9.0 ± 0.3, and 19 ± 2 μM.) The top ATX hits (two dihydrobenzamidazolones and a tetrahydroisoquinoline) were validated as inhibitors (IC_50_ = 7 ± 2, 13 ± 2, and 1 ± 0.3 μM). Photochemical dose–response DEL screening data prioritized hits for synthesis, the rate-limiting step in DEL lead identification.

DNA-encoded library (DEL) screening is an important tool for high-throughput empirical analysis of novel chemical space. DELs are synthesized using the split-and-pool strategy to generate small molecule ligands (<600 Da) covalently attached to a DNA tag that encodes the compound’s structure.^1^ Ligand candidates are isolated from these “on-DNA” libraries by affinity selection against a target protein, and subsequent DNA sequencing reveals the compound’s structures for synthesis and further characterization. DEL technology has been applied to hit identification for hundreds of targets^2^ and has delivered several clinical assets.^3−7^

As DEL technology has taken root across academia and industry, efforts to enhance the robustness of screening hit identification have intensified. DEL significantly reduces the cost of library sourcing and screening compared to high-throughput screening (HTS),^8^ but DEL hit validation is a major hurdle. To improve validation rates, selection experiments are extensively optimized. Strategies include maximizing target protein purity, selection at multiple target concentrations, counter selections against irrelevant or off targets, and selection in replicate.^6,8−10^ Even rigorous affinity selection protocols generate false positives, so small-scale on-DNA hit synthesis and target binding assay technologies have emerged as early steps during validation.^11−14^ A subset of hits is selected for scaled synthesis, secondary assay, and further follow-up studies. DEL selection and hit synthesis procedures emphasize the importance of high-quality screening data.

Like DEL, small molecule HTS relies on optimized assay designs to streamline validation studies. HTS assays are designed to achieve robust discrimination of positive and negative control compounds, as assessed by the *Z*′ score. A *Z*′ score >0.5 indicates suitability for screening, corresponding to a separation of >12 standard deviations (σ, assuming equal variance) between positive and negative control assay signals. Primary HTS experiments are almost always conducted at one compound concentration, but replicate dose–response screening dramatically reduces the false positive rate.^15^ This so-called “quantitative HTS” (qHTS) is less sensitive to assay quality, can distinguish partial inhibitors, and assigns putative hit potency during screening.

The advantages of HTS can be integrated with those of DEL using solid-phase DEL technology. Solid-phase DEL screening enables HTS-like activity assays on DEL members via microfluidic compartmentalization of polyvalent DEL beads.^16,17^ Libraries are synthesized on a photocleavable linker allowing measurement of their biochemical activity free from interference by the DNA tag and the bead surface.^17,18^ Of note, the amount of compound liberated during photocleavage is directly proportional to UV irradiation intensity,^19^ unlocking the ability to conduct qHTS-like screens of a DEL.

Here, we apply the principles of dose–response qHTS to solid-phase activity-based DEL screening using factor Xa (FXa) and autotaxin (ATX) as model enzyme targets. FXa is a chymotrypsin-like serine endoprotease integral to the blood coagulation cascade, while ATX is a lysophospholipase indicated in inflammation and cancer. Following microfluidic assay development, we synthesized and screened a 55,296-member 2-cycle solid-phase DEL at two different UV intensities for each target. The hit rates were different for each target and UV dependent with conforming behavior: lower UV intensity resulted in lower overall hit rates. Sequencing of hit collections revealed clear UV intensity-dependent trends in building block representation, highlighting the most promising hit series for synthesis and validation.

We designed and synthesized a solid-phase DEL to investigate the feasibility of UV intensity-dependent activity-based screening. The 55,296-member combinatorial library was prepared via split-and-pool DNA-encoded solid phase synthesis:^16^ 192 amino acids were used in the first cycle of chemistry, followed by 288 carboxylic acids in the second cycle (Figure 1A). Building blocks (BBs) were selected to skew the library toward lead-like chemical space (Figure S1, Tables S1 and S2). Analyzed computationally,^20^ the photocleaved library members possessed favorable molecular weight (98% of library <500 Da) and hydrophobicity (99.8% cLogP < 5) (Figure 1B). Synthesis proceeded on a mixture of resin sizes, where smaller resin (10 μm diameter) was used for library screening and larger resin (160 μm diameter) was included for library quality control (QC). Following library synthesis, the larger QC resin was separated from the screening resin and single beads were analyzed by DNA sequencing and MALDI-TOF mass spectrometry to confirm that the DNA sequence matched the predicted library member’s mass.^16,17^ The predicted library members’ masses largely matched the predicted structures (20/23 spectra matched; Table S3).

**Figure 1:**
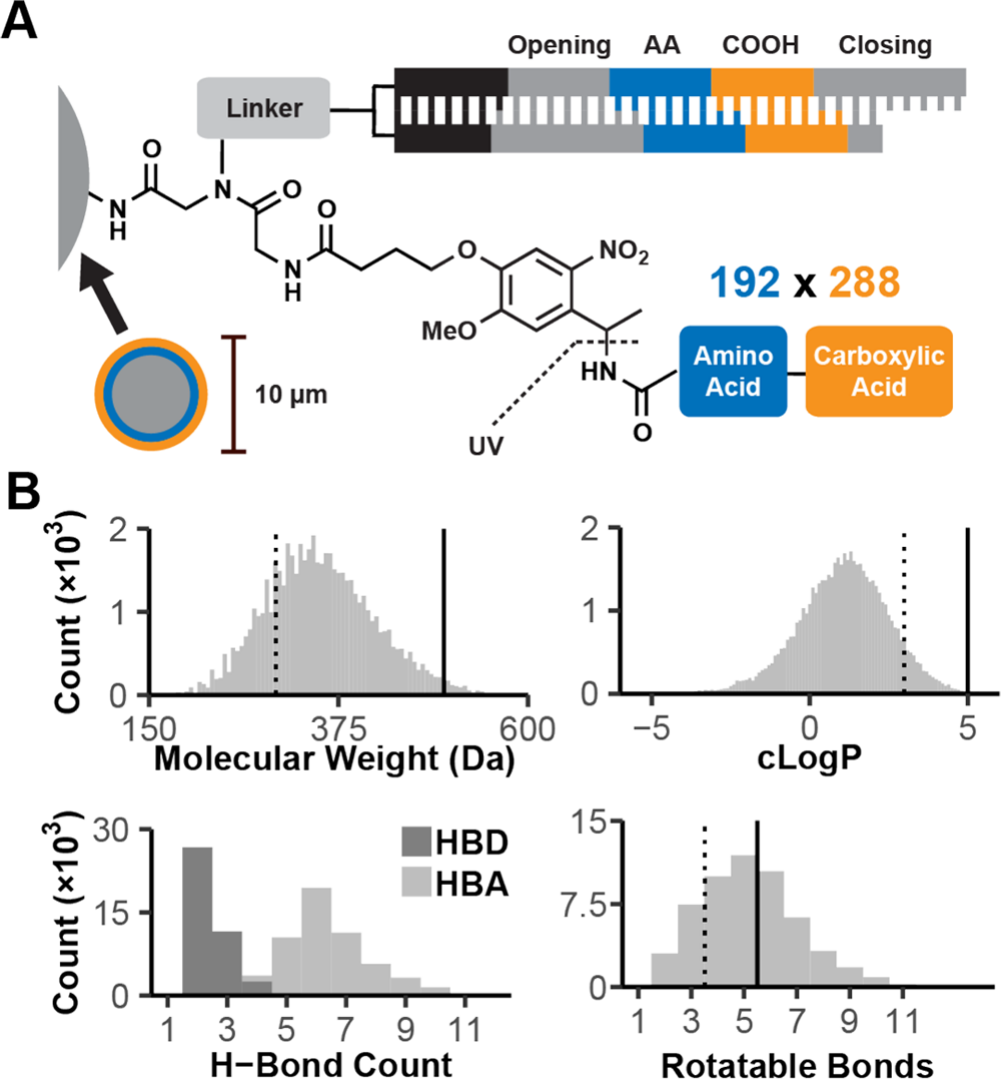
DEL structure and property distributions. (A) DNA-encoded solid-phase synthesis yielded a 55,296-member DEL via two cycles of split-and-pool synthesis. In the first cycle, 192 Fmoc-protected amino acids (AA) were coupled and encoded by their corresponding DNA tag (blue). Following Fmoc deprotection, 288 carboxylic acids (COOH) were coupled and encoded (orange). Library members were synthesized on a photocleavable *o*-nitroveratryl linker (photocleavage junction indicated). (B) Library member properties including molecular weight, *n*-octanol/water partition coefficient (clogP), hydrogen donor and acceptor count (HBD, HBA), and rotatable bond count were calculated for photocleaved primary amides. Vertical lines indicate the upper thresholds of “rule of 3” fragment-like (dashed) and “rule of 5” (solid) drug-like properties.

The DEL design featured a robust reaction sequence that yielded advantageous chemical matter. The 2-cycle library was constructed using high-yielding, DNA-compatible amide bond formation, which minimized synthesis variability^21^ and explored diversity around one of the most prevalent transformations in medicinal chemistry.^22^ The library design incorporated amino acids and carboxylic acids (Tables S1 and S2), two of the largest commercially available BB classes. Biasing the final library composition toward drug-like or even fragment-like properties further followed recent trends in DEL design.^23^ Collectively, these design principles yielded a validated ∼55k-member DEL suitable for dose–response studies.

Droplet-scale assays were optimized for dose–response DEL screening using FXa and ATX. The FXa assay detected protease activity upon cleavage of a fluorogenic FXa peptide substrate (Figure 2A) with excellent performance (*Z*′ = 0.7) using the FXa inhibitor gabexate mesylate as the positive control (Figure S2). We compartmentalized FXa, peptide substrate, and DEL beads in microfluidic water-in-oil droplets and measured hit rates at 25%, 0%, and 100% UV intensity (Figure 2B). Droplet generation and bead occupancy remained stable, while the overall hit rate and extent of target inhibition varied with the UV intensity and sorting threshold (Figure 2C). ATX screening used a previously disclosed and commercially available fluorogenic substrate that detected phosphodiesterase activity upon substrate hydrolysis^17^ (Figure 2D), and assay quality remained excellent (*Z*′ = 0.6) using a fluorous oil continuous phase (Figure S3). ATX, substrate, and DEL beads were combined in droplets as described above and evaluated at 100%, 0%, and 10% UV intensity (Figure 2E). Similarly, droplet generation and bead occupancy were stable, while the hit rate and the maximum extent of droplet inhibition varied with the UV intensity (Figure 2F). Photocleavable fluorescein (FAM) beads were used as a surrogate for DEL library members to confirm UV-dependent dosing in droplets,^19^ and we observed 90 μM, 60 μM, and 50 μM FAM in droplets at 100%, 30%, and 20% UV doses, respectively (Figure S4).

**Figure 2:**
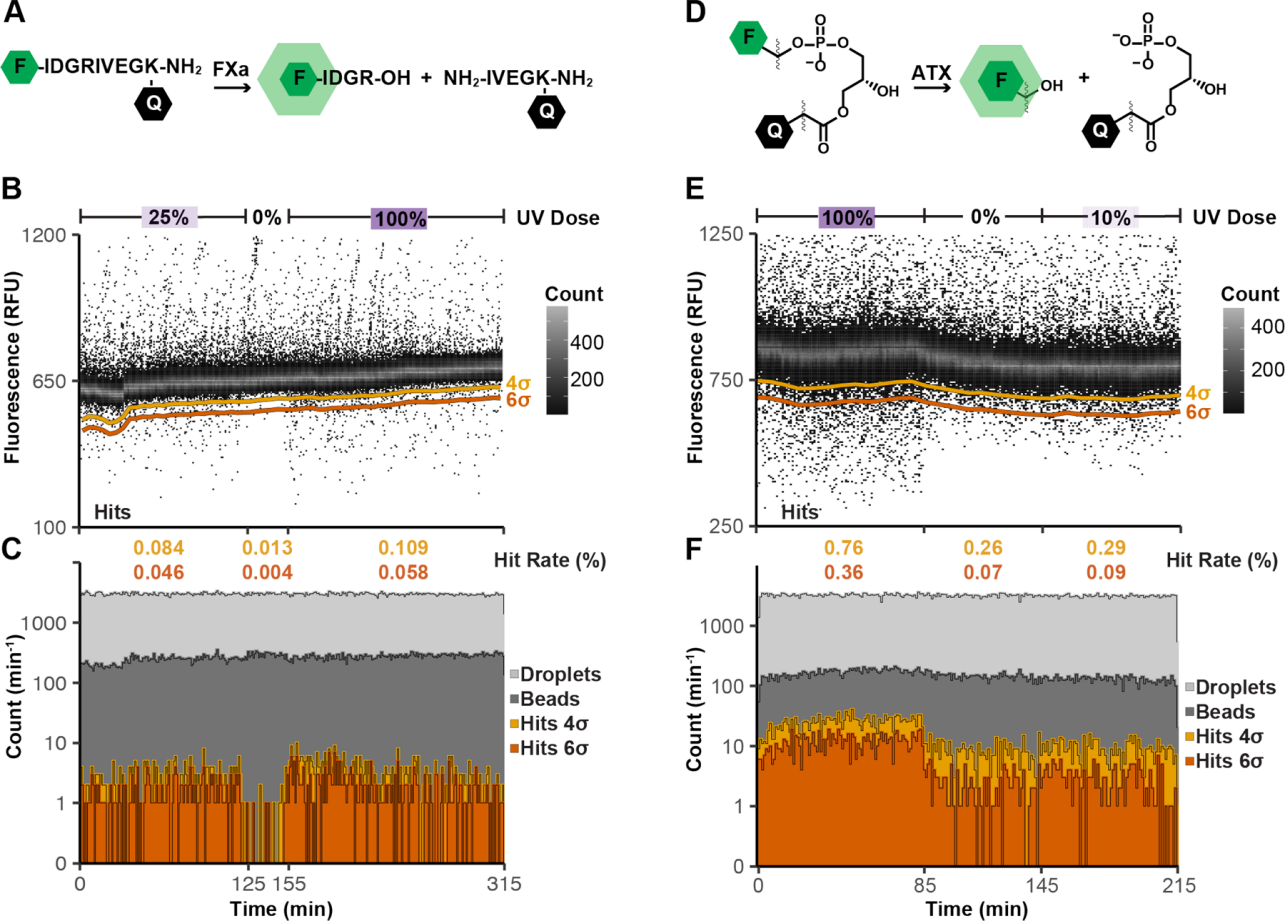
Dose–response activity-based DEL screen for FXa and ATX inhibitors. (A) The FXa fluorogenic substrate is a fluorophore–quencher pair (F/Q) flanking a FXa peptide cleavage site. FXa cleavage liberates F from Q, increasing fluorescence. (B) DEL beads combined with FXa (45 nM) and substrate (3 μM) in microfluidic droplets were screened at three different UV intensities (100%, 0%, and 25% of maximum), yielding UV intensity-dependent hit rates. Data were binned by time and fluorescence (1 min, 5 RFU) and the dynamic sorting threshold (4 and 6 standard deviations, 4σ and 6σ, below the mean) is indicated (gold, orange). (C) Droplet, bead, and hit count were binned in intervals (1 min) and plotted. (D) The ATX substrate is a F/Q pair linked through a phosphodiester bond. ATX cleaves the phosphodiester bond to liberate F from Q, increasing fluorescence. (E) DEL beads combined with ATX (50 nM) and substrate (5 μM) in microfluidic droplets were screened at three different UV intensities (100%, 0%, and 10% of maximum), yielding UV intensity-dependent hit rates. Data were binned by time and fluorescence (1 min, 5 RFU) and the dynamic sorting threshold (4σ and 6σ below the mean) is indicated (gold, orange). (F) Droplet, bead, and hit count were binned in intervals (1 min) and plotted.

Initial UV dosing experiments with FXa and ATX confirmed the hypothesis that dose–response screening would yield UV-dependent hit rates. The clear relationship observed between UV intensity and hit rate for FXa and ATX indicated that the DEL was productive against both targets. For FXa, 4.5σ below the mean negative droplet signal (μ – 4.5σ) was selected as the sorting threshold for future screening, as this differentiated hit rate between all 3 UV intensities. Alternatively, for ATX, a larger μ – 6σ sorting threshold was selected, as this more stringently discriminated between 10% and 0% UV intensities. Under these sorting conditions, the maximum hit rate was ∼3-fold lower for FXa than for ATX (0.08% vs 0.24% at 100% dosing), implying that FXa was inhibited by fewer library members. These FAM dosing and initial DEL screening results supported the hypothesis that modulating UV intensity would affect hit identification and potentially differentiate between low and high μM IC_50_ inhibitors. However, demonstrating that dose–response screening would provide meaningful insight into structure–activity relationships required exhaustive DEL screening^24^ at different UV intensities.

Exhaustive FXa screening was performed at high (100%) and low (30%) UV intensity, identifying several hit series. Collectively, ∼9 and ∼13 library equivalents (9ε = 9 × 55,296 ≈ 500k DEL beads; 13ε ≈ 720k DEL beads) were screened at 100% and 30% UV intensities, yielding 0.08 ± 0.02% and 0.05 ± 0.02% hit rates, respectively (Table S4). Droplets containing hit beads were sorted, and the beads were pooled according to their screening condition. Encoding tags from isolated hit beads were amplified (Table S5) and sequenced for hit structure determination (Figure 3). Benzothienyl alanine **1**, d-tryptophan **2**, piperidin-4-yl proline **3**, and piperazine-2-carboxylic acid **4** were the most highly represented amino acid BBs. The cumulative replicate count, or *k* class,^24^ for these building blocks was 111, 54, 111, and 38, respectively (Figure S5). Chlorothiophene **5** and 1-phenylpyrrolidin-2-one **6** were the most enriched carboxylic acids. Their cumulative *k* classes were 93 and 81, and were followed by structurally related acids **7**–**9** with cumulative *k* classes of 48, 39, and 31 (Figure S6). These amino acid and carboxylic acid hit series all showed enrichment across both screening doses.

**Figure 3:**
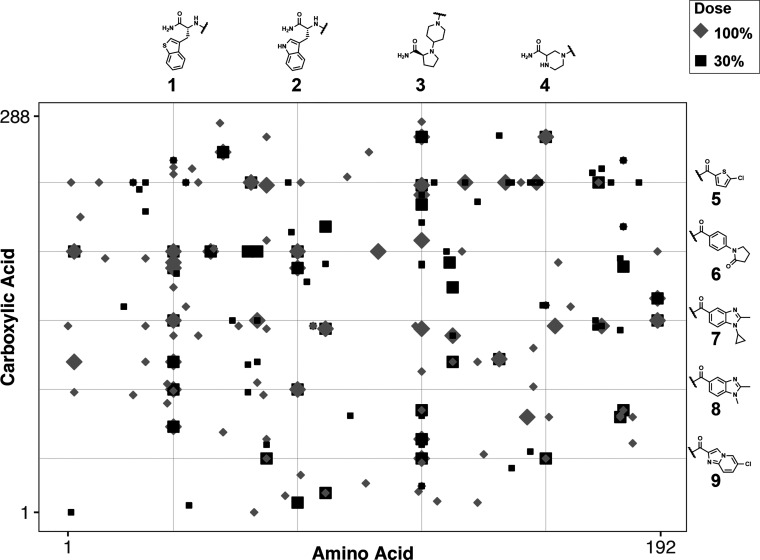
FXa dose–response DEL screening hit deconvolution. Data point shape and shading indicate UV intensity, and data point size indicates hit replicate count (*k* class). Small points indicate *k* = 2–3 and large points indicate *k* = 4–13, while black squares represent 30% dosing and gray diamonds represent 100% dosing. The top 4 amino acid and the top 4 carboxylic acid hit series are displayed.

FXa hit representation data suggested that screening successfully identified the FXa inhibitors. The top amino acid hit series share structural similarity with known potent FXa inhibitors (Table S6). As examples, **1** and **2** resemble substituted benzothiophene and indole compounds that are known FXa inhibitors.^25,26^ Amino acid **3** resembles the S4 binding motif of TAK-442, an orally active FXa inhibitor.^27^ Similarly, the FXa carboxylic acid hit series recapitulated features of potent FXa inhibitors (Table S7). The most represented carboxylic acid, chlorothiophene **5**, shares the S1 binding moiety of highly potent rivaroxaban (IC_50_ = 0.7 nM), while the second most enriched carboxylic acid **6** shares the S4 binding motif of a marginally less potent (IC_50_ = 4 nM) rivaroxaban derivative.^28^ Benzimidazoles **7** and **8** reproduce additional FXa inhibitory chemotypes.^29^

Dose–response data further supported the determination of the major FXa hit series. The top amino acid and carboxylic acid hit series were highly represented in both the high and low UV intensity screens, boosting confidence in their authenticity. Additionally, several distinct disynthons were highly represented at both doses, suggesting that dose–response screening was useful for prioritizing hit series for synthesis and further study. FXa screening did not yield any hit series exclusively enriched at the high dose alone. Hits from the highly represented series may have similar potencies that cannot be distinguished from one another given the applied screening conditions.

A set of FXa hits was validated following synthesis and purification. Hits were selected on the basis of high *k* class at both doses (Table 1). Hits **1a** and **1b**, both of which are from the benzothienyl alanine hit series, robustly inhibited FXa (IC_50_ = 4.2 ± 0.1 and 7.4 ± 0.3 μM). Hit **2a**, the d-trytophan analogue of **1a**, was similarly potent (IC_50_ = 9.0 ± 0.3 μM). Hit **3a** explored an amino acid hit series defined by pyrrolidinyl piperidine and weakly inhibited FXa at 100 μM (Figure S7). Finally, hit **5a**, which explored the hit series defined by chlorothiophene carboxylic acid **5**, moderately inhibited FXa (IC_50_ = 19 ± 2 μM).

**Table 1:**
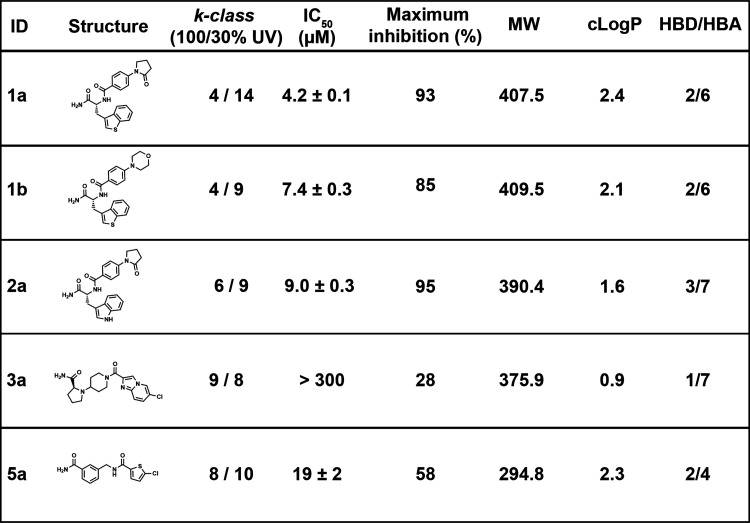
FXa Screening Hit Validation

Dose–response FXa screening successfully identified authentic FXa inhibitors from several chemotypes. Known FXa inhibitory chemotypes from rivaroxaban^28^ coincided with the most enriched hit compounds, but these hits explored alternative adjacent functionality. Hits **1a**, **1b**, and **2a** explored similar functional groups and exhibited similar potency. The relative potency of **1a** and **1b** was consistent with the potency of phenylphenylpyrolidin-2-one and phenylmorpholine S4 binding motifs in rivaroxaban derivatives,^28^ despite lower potency of these compounds relative to rivaroxaban. Comparing **2a** with **1a**, which only differs by an S to NH substitution, IC_50_ was slightly increased. Hit **3a** inhibited FXa only at high concentrations and appears to be a genuine, albeit weak, FXa inhibitor. Finally, **5a**, which contains the S1 chlorothiophene binding motif, was moderately potent, indicating the importance of a corresponding S4 binding motif to improve binding. Collectively, these data demonstrate that the applied screening approach reliably identified valid hits.

To extend this approach to a mechanistically distinct target, exhaustive dose–response DEL screening was performed by using the ATX activity assay. Collectively, ∼7ε and ∼6ε (∼330k and ∼390k) DEL beads were screened at 100% and 20% UV intensities, yielding hit rates of 0.24 ± 0.2% and 0.08 ± 0.01%, respectively (Table S8). DNA encoding sequences were amplified from hit beads and sequenced for hit structure determination (Figure 4). Many hit series (**10**–**18**) were evident, but five amino acid hit series (**10**, **11**, **13**, **16**, and **17**) were enriched with cumulative *k* classes of 148, 79, 124, 81, and 67, respectively (Figure S8). Notably, phosphonomethyl-phenylalanine **10**, phosphotyrosine **11**, dihydrobenzamidazolone **16**, and tetrahydroisoquinolone **17** were isolated from both 100% and 20% UV intensity screens, while tricyclic amino acid **13** was only isolated during the 100% UV screen. Carboxylic acid hit series were less prominent, but BBs **19**–**23** showed conservation with cumulative *k* classes of 31, 23, 36, 46, and 41, respectively (Figure S9).

**Figure 4:**
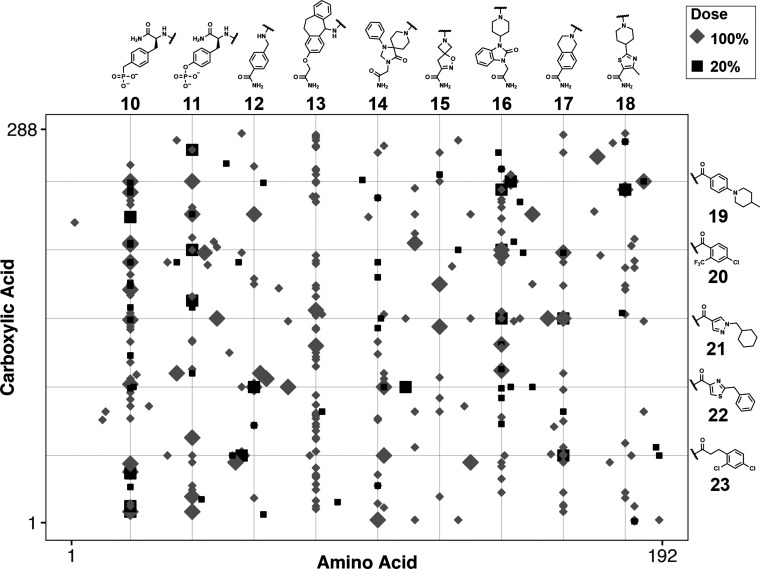
ATX dose–response DEL screening hit deconvolution. Data point shape and shading indicate UV intensity, and data point size indicates hit replicate count (*k* class). Small points indicate *k* = 2–3 and large points indicate *k* = 4–11. Black squares represent 20% UV intensity, and gray diamonds represent 100% UV intensity. The top hits are displayed.

Photodose hit enrichment patterns in ATX screening data identified and prioritized hit series. Several ATX hit BBs resemble known ATX inhibitory fragments (Tables S10 and S11). For example, top ATX hit series **10**, **14**, **22**, and **23** all recapitulate previously validated activity- and affinity-based DEL screening results,^7,17^ while hit series **16** and **17** resemble ATX inhibitory fragments identified from HTS experiments.^30^ Tricyclic amino acid **13**, a previously unreported ATX inhibitor, was interesting from a screening perspective as it was highly conserved (cumulative count rank 2), but it was never observed in any hits when the library was screened at 20% UV. Hit series **12** and **15** similarly were not enriched in the 20% UV screen outside of select BB combinations. Collectively, UV intensity-dependent hit representation highlighted series **10**, **11**, **16**, and **17** as high-priority hit series while implying that **12**, **13**, and **15** might be less potent.

Dose–response DEL screening for ATX inhibitors informed the hit selection for synthesis and validation in microplates. Hits from series **16** and **17**, which were enriched at both UV intensities, were deemed high-priority hits. Hits from series **12** and **13** were not represented well or at all in the 20% UV screen and thus were deemed lower priority. Hits from series **10**, **11**, and **14**, despite high representation, were not investigated further due to their discovery in previously reported ATX screens.^7,17^ Of the synthesized hits, 6/7 inhibited ATX with relative IC_50_ values 1–27 μM (Table 2, Figure S10). Low-priority hits **12a** and **12b** were the least potent hits and a false positive, respectively. Low-priority hits **13a** and **13b** were moderately potent (IC_50_ = 6 μM for both) and only partially inhibited ATX (57% and 26% inhibition). High-priority hits **16a**, **16b**, and **17a** were the most potent (IC_50_ = 7, 13, and 1 μM, respectively) and inhibited ATX fully.

**Table 2:**
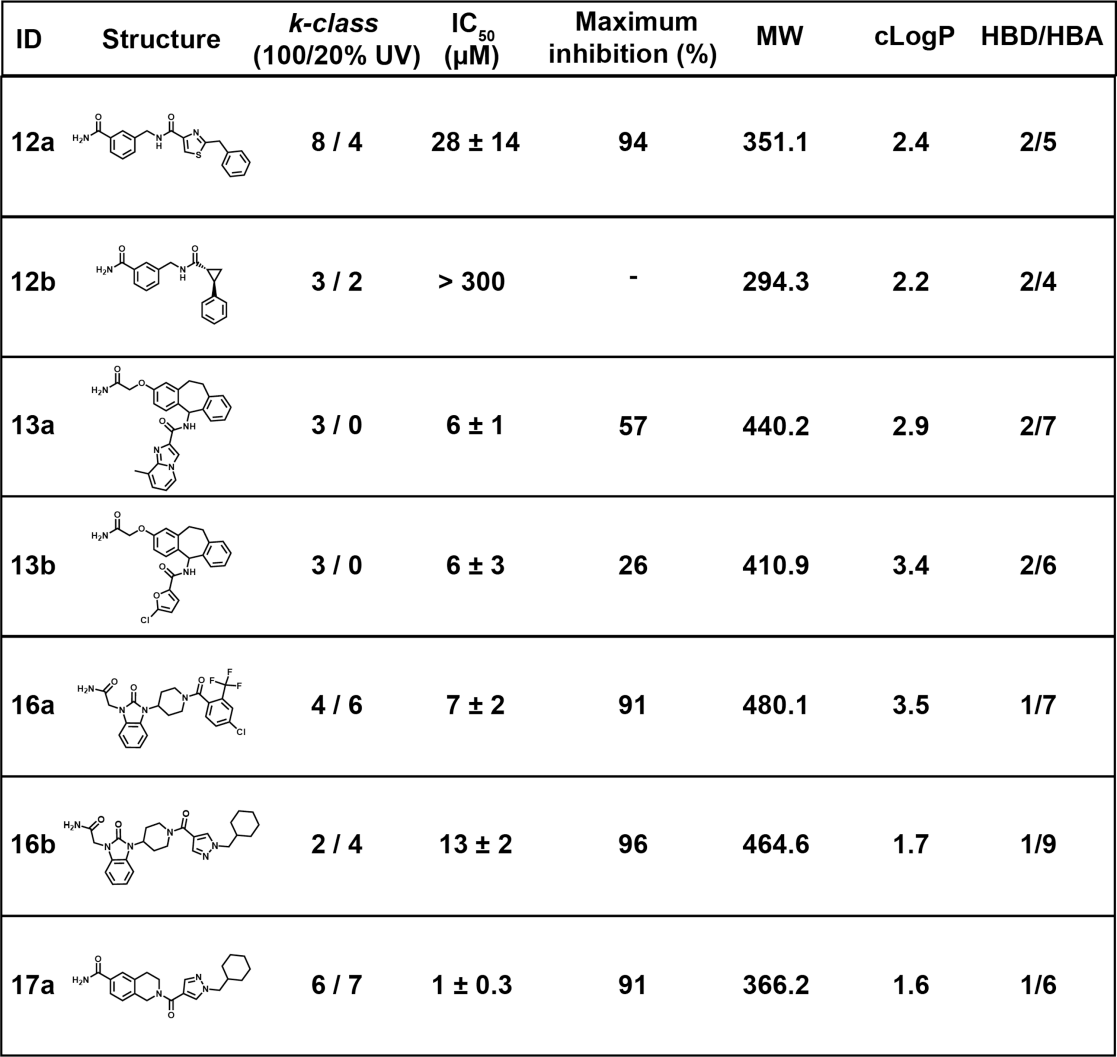
Autotaxin Screening Hit Validation

ATX hit synthesis validated dose–response DEL screening as a technique for prioritizing hits. Low-priority ATX hits **13a**, **13b**, **14a**, and **14b** weakly, did not, or partially inhibited ATX, while high-priority hits **16a**, **16b**, and **17a** more potently and completely inhibited ATX. Partial ATX inhibitors **13a** and **13b** would have been missed if ATX was only screened at the lower 20% UV intensity. Hit **12b**, which did not inhibit ATX, was enriched at a low level (*k* = 3/2 at 100/20% UV) bordering on the experimental noise level for screening. While exhaustive hit synthesis and testing was not possible, our hit validation together with known ATX SAR for hit series **10**, **14**, **22**, and **23** supports the hypothesis that dose–response DEL screening increases confidence in hit identification and may also predict hit potency.

Dose–response DEL screening critically expands the number of metrics available for assessing a screening hit’s authenticity prior to committing effort to synthesis. Replicate hit identification^24^ solely guided previous DEL screening efforts.^17,18^ Random library sampling and experimental variability sometimes clouded relationships between hit enrichment and quality. With the additional library screening UV intensity data, we could analyze BB enrichment under different experimental conditions to prioritize hits. However, potential sources of experimental variation include synthesis yield or truncate noise,^21^ low photochemical cleavage yield of hydrophobic compounds,^31^ or compound retention in droplets.^32,33^ Presently, dose–response screening has been demonstrated using a 2-cycle library. Screening 3-cycle libraries may introduce additional challenges stemming from increased synthesis variability.^21^ Library sampling variability is still an important consideration, but as in qHTS,^15^ dose–response DEL screening transforms the process from a primarily statistical endeavor to a biochemical experiment.

Our results further suggest that dose–response DEL screening could predict hit potency from the primary screening data. Across both screens, target inhibition in droplets was dependent on the applied UV intensity, and hit enrichment patterns were consistent with a dose–response relationship. Explicit quantitative mapping of inhibitor potency was not possible because our microfluidic device conducts binary sorting. Droplets are sent to waste or to hit collection; a hit that strongly inhibits the target activity well below the sorting threshold is indistinguishable from a hit that weakly inhibits the target slightly below the sorting threshold. Multiplexed droplet sorting could be implemented to sort hits at several sorting thresholds (i.e., 4σ and 8σ) in a single screening experiment.^34^ Combining multiplexed threshold sorting with more granular UV photochemical dosing could further enhance the resolution of DEL hit identification, perhaps approaching the exquisite capabilities of qHTS.

Future advances in dose–response DEL screening technology are likely to provide improvements in screening data quality, but the assay sensitivity and screening format are important considerations. Estimating the concentration of library member liberated during photocleavage using photocleavable fluorescein beads, the highest observed concentration in droplets was 90 μM. In conjunction with the sorting threshold, this establishes limitations for identifying weak inhibitors. While this estimate agreed with the potency of hits identified, additional droplet mass spectrometry experiments^33^ could evaluate bead photocleavage in droplets for compounds that are more representative of DEL members. However, even lacking this more precise level of library member-specific photocleavage efficiency data, DEL productivity is readily apparent from even brief droplet experiments. These data would expedite a pivot to alternate libraries for targets that may not elicit a detectable hit rate even at the highest UV intensity. Some assay formats, such as competition binding,^18^ require the screening compound to saturate target binding in the presence of a potent probe molecule and these screens may only be productive at high compound concentrations.

In this work, we successfully modulated photochemical compound release to realize dose–response activity-based DEL screening. We demonstrated its benefits in screening two exemplary enzyme drug targets. High-priority hits were identified from a drug-like DEL based on replicate identification during screening with high and low compound photocleavage. Dose–response DEL screening data also demonstrated the underlying principles for determining hit potency during primary screening, the resolution of which will only improve as sorting circuitry, DEL design, and assay complexity continue to evolve.

## Supporting Information Available

Experimental methods including DEL synthesis, DEL screening, hit deconvolution, and synthesis; supplementary tables and figures describing DEL synthesis, DEL building block and hit structures, DEL screening data, and activity assay data (PDF)

## Abbreviations

DEL: DNA-encoded library
ATX: autotaxin
FXa: factor Xa
HTS: high-throughput screening
qHTS: quantitative high-throughput screening
HBD: hydrogen bond donor
HBA: hydrogen bond acceptor
