# Supplementary material for: Dose–Response Activity-Based DNA-Encoded Library Screening
Source: ACS Med Chem Lett. 2023 Aug 21;14(9):1295–303. doi: 10.1021/acsmedchemlett.3c00159 (PMC10510511; doi:10.1021/acsmedchemlett.3c00159)
Supplement: Supplementary file 1 — ml3c00159_si_001.pdf [file ml3c00159_si_001.pdf]

Supporting Information: Dose-Response Activity-Based DNA-Encoded Library Screening

Patrick R. Fitzgerald,[†] Wesley G. Cochrane,[‡,§] and Brian M. Paegel[*,‡,¶]

†*Skaggs Doctoral Program in the Chemical and Biological Sciences, Scripps Research, La Jolla, California 92037, United States*

‡*Department of Pharmaceutical Sciences, University of California, Irvine, California 92697, United States*

¶*Departments of Chemistry & Biomedical Engineering, University of California, Irvine, California 92697, United States*

§*Present Address: The Salk Institute for Biological Studies, La Jolla, California 92037, United States*

E-mail: bpaegel@uci.edu

Contents

Materials and Methods **S-6**

Safety Statement S-6

Materials S-6

Oligonucleotides S-7

Buffers S-7

Autotaxin Preparation S-8

Factor Xa Preparation S-8

Solid-Phase DNA-Encoded Library Synthesis S-9

DEL Bifunctional Library Resin Synthesis and Characterization S-9

DEL Resin Barcoding S-10

DEL Ligation of Bead Barcoding (≈0002, ≈11XX, and ≈22XX) Oligonucleotides S-11

DNA-Encoded Solid-Phase Combinatorial Library Synthesis S-11

DEL Amino Acid Coupling, Fmoc Removal S-12

DEL Ligation of ≈13XX and ≈24XX Amino Acid Encoding Oligonucleotides S-12

DEL Carboxylic Acid Coupling S-12

DEL Ligation of ≈15XX and ≈26XX Carboxylic Acid Encoding Oligonucleotides S-13

DEL Ligation of ≈0B02 Oligonucleotide S-13

Solid-Phase DNA-Encoded Library QC: 10-µm Bead Analysis S-13

Solid-Phase DNA-Encoded Library QC: 160-µm Bead Analysis S-14

Resin Cleavage and MALDI-TOF MS Analysis S-15

Microfluidic Device Fabrication S-16

Microfluidic Device Operation S-17

Droplet-Scale Flow Injection Analysis of FXa Activity Assay Quality S-17

Droplet-Scale Flow Injection Analysis of ATX Activity Assay Quality S-18
FXa Library Screening in Droplets S-18
ATX Library Screening in Droplets S-19
Synthesis of Photocleavable Fluorescein Beads (PC-Gly-FAM Beads) S-20
Measurement of Fluorescein in Droplets Following Bead Photocleavage. S-22
Hit Bead Isolation S-23
Hit Bead Amplification and Preparation for Illumina High-Throughput SequencingS-24
DNA Sequencing and Data Processing S-24
Biochemical Assay for FXa Hit Validation S-25
Biochemical Assay for ATX Hit Validation S-25
Modeling ATX Competitive Inhibition S-26
Modeling ATX Noncompetitive Inhibition S-26
Modeling ATX Uncompetitive Inhibition S-27

Compound Synthesis and Characterization **S-28**

NMR Analysis S-28
LC-MS Analysis S-28
Semi-Preparative HPLC Purification S-28
Synthesis of 1a S-29
Synthesis of 1b S-30
Synthesis of 2a S-31
Synthesis of 3a S-32
Synthesis of 5a S-33
Synthesis of 12a S-35
Synthesis of 12b S-37
Synthesis of 13a S-39
Synthesis of 13b S-41
Synthesis of 16a S-43

Synthesis of 16b S-44
Synthesis of 17a S-45

Supporting Tables **S-47**

Table S1 . . . . . S-47
Table S2 . . . . . S-53
Table S3 . . . . . S-59
Table S4 . . . . . S-62
Table S5 . . . . . S-63
Table S6 . . . . . S-64
Table S7 . . . . . S-65
Table S8 . . . . . S-66
Table S9 . . . . . S-67
Table S10 . . . . . S-68
Table S11 . . . . . S-69

Supporting Figures **S-72**

Figure S1 . . . . . S-72
Figure S2 . . . . . S-73
Figure S3 . . . . . S-74
Figure S4 . . . . . S-78
Figure S5 . . . . . S-78
Figure S6 . . . . . S-78
Figure S7 . . . . . S-78
Figure S8 . . . . . S-82
Figure S9 . . . . . S-82
Figure S10 . . . . . S-82
Figure S11 . . . . . S-82

Figure S12 . . . . S-83
Figure S13 . . . . S-84
Figure S14 . . . . S-85
Figure S15 . . . . S-86
Figure S16 . . . . S-87
Figure S17 . . . . S-88
Figure S18 . . . . S-89
Figure S19 . . . . S-90
Figure S20 . . . . S-91
Figure S21 . . . . S-92
Figure S22 . . . . S-93
Figure S23 . . . . S-94
Figure S24 . . . . S-95
Figure S25 . . . . S-96
Figure S26 . . . . S-97

Supporting Schemes **S-98**

Scheme S1 . . . . S-98

Materials and Methods

Safety Statement

No unexpected or unusually high safety hazards were encountered.

Materials

All reagents were obtained from MilliporeSigma (St. Louis, MO) unless otherwise specified. 1,3-Bis[tris(hydroxymethyl)methylamino]propane (Bis-Tris), tris(hydroxymethyl)-amino-methane (Tris), 2-(N-morpholino)ethanesulfonic acid (MES), trifluoroacetic acid (TFA), triisopropylsilane (TIS), α-cyano-4-hydroxycinnamic acid (HCCA, Life Technologies, Carlsbad, CA), N,N′-diisopropylcarbodiimide (DIC, Acrōs Organics, Fair Lawn, NJ), N-α-Fmoc-N-ε-7-methoxycoumarin-4-acetyl-L-lysine (N-α-Fmoc-K(Mca)-OH), N-$_{\alpha}$-Fmoc-N$_{\omega}$-(2,2,4,6,7-penta- methyldihydrobenzofuran-5-sulfonyl)-L-arginine (Fmoc-R(Pbf)-OH, Thermo Fisher Scientific, Waltham MS), 4-{4-[1-(9-Fluorenylmethyloxycarbonylamino)ethyl]-2-methoxy-5-nitrophenoxy}butanoic acid (Fmoc-PC-OH, Advanced Chemtech, Louisville, KY), Taq DNA polymerase (Taq, New England Biolabs, Ipswich, MA), 2′-deoxyribonucleotide triphosphates (dNTP, set of dATP, dTTP, dGTP, dCTP, New England Biolabs, Ipswich, MA), Pico-Surf™ fluorosurfactant (5% solution in Novec-7500™, Sphere Fluidics, Cambridge, UK), Novec-7500™ (3M, Saint Paul, MN), factor Xa (R&D Systems, Minneapolis, MN), 5-FAM/QXL™-520 factor Xa substrate (AnaSpec, Fremont, CA), autotaxin (Echelon Biosciences, Salt Lake City, UT), FS-3 Autotaxin substrate (Echelon Biosciences, Salt Lake City, UT), SYBR-green (Invitrogen, Waltham, MA), Pluronic® F-127, 3-[(3-Cholamidopropyl)-dimethylammonio]-1-propanesulfonate hydrate (CHAPS, G-Biosciences), 1,4-dithiothreitol (DTT), copper (II) sulfate ($CuSO_4$), ascorbic acid (Acrōs Organic), tris(benzyltriazolylmethyl)-amine (TBTA), ethylenediaminetetraacetic acid (EDTA, BioRad), triethylammonium acetate (TEAA), 1-hydroxy-7-azabenzotriazole (HOAt, Accela ChemBio Inc., San Diego, CA), 2,4,6-

trimethyl-pyridine (TMP), ethyl 2-cyano-2-(hydroxyimino)acetate (Oxyma), dimethylformamide (DMF, Thermo Fisher Scientific), dichloromethane (DCM, Thermo Fisher Scientific), N,N-dimethylacetamide (DMA, Acrōs Organics), N,N-diisopropylethylamine (DIEA, Thermo Fisher Scientific), acetonitrile (ACN, Thermo Fisher Scientific), dimethylsulfoxide (DMSO, AMRESCO Inc., Solon, OH), magnesium chloride ($MgCl_2$), poly(dimethylsiloxane) (PDMS, Dow Corning, Midland, MI), sodium acetate, and calcium chloride, were used as provided. Solvents used in solid-phase synthesis were dried over molecular sieves (3 Å, 3.2 mm pellets).

Synthesis on mixed-scale resin was performed on TentaGel Rink Amide resin (160 µm, 0.43 mmol/g, Rapp-Polymere, Tübingen, Germany) and TentaGel amino functionalized resin (10 µm, 0.29 mmol/g, Rapp-Polymere, Tübingen, Germany). Hit synthesis was performed on Fmoc-Rink Amide MBHA resin (0.44 mmol/g, AnaSpec) or Sieber amide resin (0.57 mmol/g, Aaptec, Louisville, KY). N-Fmoc amino acid and carboxylic acid building blocks were selected from a collection of available sources (Combi-Blocks Inc., San Diego, CA; ChemImpex, Wood Dale, IL; and Enamine Ltd., Kyiv, Ukraine).

Oligonucleotides

Oligonucleotides (Integrated DNA Technologies, Inc. Coralville, IA) were purchased as desalted lyophilate and used without further purification. Oligonucleotide ligation substrates were 5′-phosphorylated (/5Phos/). Amino-modified headpiece DNA (NH_2-HDNA, /5Phos/GAGTCA/iSp9//iUniAmM//iSp9/TGACTCCC) was HPLC purified at the manufacturer and used without further purification.

Buffers

Bis-Tris propane wash buffer (BTPWB, 50 mM NaCl, 0.04% Tween-20, 10 mM Bis-Tris, pH 7.6), Bis-Tris propane breaking buffer (BB, 10 mM NaCl, 1% SDS, 1% Tween-20, 10 mM

Bis-Tris, pH 7.6), 10X Bis-Tris propane ligation buffer (10X-BTPLB, 500 mM NaCl, 100 mM $MgCl_2$, 10 mM ATP, 0.2% Tween-20, 100 mM Bis-Tris propane, pH 7.6), GC-PCR buffer (0.2 mM dATP, 0.2 mM dTTP, 0.2 mM dCTP, 0.2 mM dGTP, 50 mM KCl, 2.5 mM $MgCl_2$, 1M Betaine, 6% DMSO (v/v), 0.2X SYBR green, 0.05 U/µL Taq, 10 mM Tris, pH 8.0), ATX storage buffer (140 mM NaCl, 20% glycerol (v/v), 1 mM $MgCl_2$, 1 mM $CaCl_2$, 3 mM KCl, 0.025% CHAPS (w/v), 50 mM Tris, pH 8.0), ATX droplet assay buffer (140 mM NaCl, 18% sucrose (w/v), 1 mM $MgCl_2$, 1 mM $CaCl_2$, 3 mM KCl, 0.04% Pluronic F127 (w/v), 50 mM Tris, pH 8.0), ATX microwell-plate assay buffer (140 mM NaCl, 1 mM $MgCl_2$, 1 mM $CaCl_2$, 3 mM KCl, 0.04% Pluronic F127 (w/v), 50 mM Tris, pH 8.0), FXa storage buffer (150 mM NaCl, 5 mM $CaCl_2$, 0.025% Pluronic F127 (w/v), 25 mM MES, pH 6.0), FXa droplet assay buffer (2 mM $CaCl_2$, 100 mM NaCl, 18% sucrose (w/v), 0.025% Pluronic F127 (w/v), 20 mM Tris, pH 8), and crush and soak buffer (C&S, 200 mM NaCl, 10 mM Tris, pH 8.0) were prepared in deionized water.

Autotaxin Preparation

Active, lyophilized autotaxin was resuspended (1 µM in ATX storage buffer), divided into aliquots, flash frozen on dry ice/isopropanol, and stored at -80 °C.

Factor Xa Preparation

Activated, lyophilized Factor Xa was resuspended according to the manufacturer's instructions with the addition of nonionic surfactant (0.025% (w/v) Pluronic® F127), divided into aliquots (100 µg/mL in FXa storage buffer), flash frozen on dry ice/isopropanol and stored at -20 °C.

Solid-Phase DNA-Encoded Library Synthesis

DEL Bifunctional Library Resin Synthesis and Characterization

All fritted-syringe wash volumes were identical (5.0 mL). Quality-control (QC) TentaGel rink amide resin (160 µm, 0.40 mmol/g, 100 mg, Rapp-Polymere, Tübingen, Germany) was transferred to a fritted syringe (10 mL, Torviq, Tucson, AZ), swelled in solvent (DMF, 1 h, RT, 8 rpm), and washed (3 × DMF). Fmoc was removed (20% piperidine in DMF, 5 mL, 1 × 5 min, 1 × 15 min, RT, 8 rpm) and the resin was washed (3 × DMF, 3 × DCM, 3 × DMF). N-α-Fmoc-K(Mca)-OH (120 µmol) was activated with COMU/DIEA (120/240 µmol) in DMF (1 mL, 2 min, RT), added to the resin, incubated (30 min, 50 °C, 8 rpm, 2×), and the resin was washed (3 × DMF, 3 × DCM, 3 × DMF). Fmoc was removed (20% piperidine in DMF, 5 mL, 1 × 5 min, 1 × 15 min, RT, 8 rpm) and the resin was washed (3 × DMF, 3 × DCM, 3 × DMF). N-α-Fmoc-R(Pbf)-OH (120 µmol) was activated with COMU/DIEA (120/240 µmol) in DMF (1 mL, 2 min, RT), added to resin, and incubated (30 min, 50 °C, 8 rpm, 2×). The resin was washed (3 × DMF, 3 × DCM, 3 × DMF).

Synthesis resin (amino-functionalized, 10 µm dia., 0.29 mmol/g, 1 g, Rapp-Polymere, Tübingen, Germany) and the aforementioned QC resin (50 mg) were transferred to a syringe (25 mL, Torviq, Tucson, AZ) fitted with two additional frits (10-µm polyethylene, 20 mm dia., Biotage ISOSOLUTE, Charlotte, NC). All fritted-syringe wash volumes were identical (10.0 mL). Pooled resin was swelled in solvent (DMF, 16 h, RT, 8 rpm), and washed (3 × DCM, 3 × DMF). Subsequent amino acid coupling cycles consisted of: (1) Fmoc removal (20% piperidine in DMF, 1 × 5 min, 1 × 15 min, RT, 8 rpm); (2) N-α-Fmoc-amino acid (2.3 mmol) activation with DIC/Oxyma/DIEA (2.8/2.8/5.6 mmol, 2 min, RT); (3) addition of activated N-Fmoc-amino acid to resin and incubation (1 h, 50 °C, 8 rpm). The N-substituted glycine coupling cycle consisted of: (1) Fmoc removal (20% piperidine in DMF, 1 × 5 min, 1 × 15 min, RT, 8 rpm); (2) bromoacetic acid (2.8 mmol) activation with DIC (5.6 mmol, 2

min, RT); (3) coupling of activated bromoacetic acid to resin (1 h, 50 °C, 8 rpm); (4) bromine displacement (1 M propargylamine, DMF, 3h, 50 °C, 8 rpm). Unless specified otherwise, following each Fmoc removal and building block coupling step, resin was washed (3 × DMF, 3 × DCM, 3 × DMF). N-α-Fmoc-Gly-OH, bromoacetic acid/propargylamine, and N-α-Fmoc-Gly-OH were coupled sequentially as described above. Fmoc-PC-OH (1.4 mmol) was activated with DIC/Oxyma/TMP (2.1/1.4/1.4 mmol), added to resin, incubated (1 × 2 h, 1 × 1 h, 37 °C, 8 rpm), and the resin was washed (3 × DMF, 3 × DCM, 3 × DMF). Azido headpiece DNA (N_3-HDNA) was prepared and attached to bifunctional resin by CuACC as previously described.[1]

DEL Resin Barcoding

All library synthesis, library handling, and microfluidic droplet-based screening were performed in a UV-free room. A general protocol for DNA-encoded solid-phase synthesis (DESPS) has previously been described.[1,2] Oligonucleotides are indicated in bold with the "≈" designation. Numeric identifiers were described previously (Table S11).[3] Oligonucleotide paired (OP) stock solutions of complementary oligonucleotides (60 µM [+], 60 µM [-], 50 mM NaCl, 1 mM Bis-Tris pH 7.6) were heated (5 min, 60 °C) and cooled to ambient (5 min, RT) before each use. OP stocks bear the [±] designation, indicating "double-stranded." Resin washing proceeded through addition of solvent (0.2 mL) and vacuum filtration (multiscreen solvinert, 96 well, PTFE, 0.45 µm pore size, 0.5 mL/well, Millipore Sigma, Burlington MA). DEL barcoding and encoded combinatorial synthesis procedures are summarized in Scheme S1.

DEL Ligation of Bead Barcoding (≈0002, ≈11XX, and ≈22XX) Oligonucleotides

All wash volumes were identical (1.0 mL). Mixed-scale bifunctional HDNA library resin was split evenly into 12 × 1.5 mL conical tubes, washed (3 × BTPWB), and supernatant was removed. Resin was washed (1 × 1X-BTPLB) and resuspended (1X-BTPLB, 1 mL), and incubated while the encoding oligonucleotide ligation mixtures were prepared (~30 min, RT). To each tube, 10X-BTPLB (100 µL), ≈0002[±] (500 µM, 184 µL), T4 ligase (4 mg/mL, 17.3 µL), and H_2O (700 µL) were added. The tubes were incubated (4 h, RT, 8 rpm) and the resin was washed (3 × BTPWB, 2 × 1X-BTPLB, 300 µL).

The supernatant was removed and to each tube was added: 10X-BTPLB (100 µL), ≈11XX [±](150 µM, 613 µL), T4 ligase (4 mg/mL, 17.3 µL), H_2O (270 µL). The reaction mixture was incubated (4 h, RT, 8 rpm). The resins were washed (3 × BTPWB), pooled (BTPWB, 12 mL) and split into 12 x 1.5 mL conical tubes. Resin in tubes was washed (2 × 1X-BTPLB).

The supernatant was removed and to each tube was added: 10X-BTPLB (100 µL), ≈22XX [±](150 µM, 613 µL), T4 ligase (4 mg/mL, 17.3 µL), H_2O (270 µL). The reaction mixture was incubated (4 h, RT, 8 rpm). Resins were washed (3 × BTPWB) and resuspended in BTPWB (100 µL).

DNA-Encoded Solid-Phase Combinatorial Library Synthesis

Barcoded, mixed-scale library resin was retrieved, washed (3 × BTPWB, 3 × 1:1 DMF:BTPWB, 3 × DMF), resuspended (DMF, 0.1 mL), pooled into a reservoir, split into 6 × 96 wells (1.7 mg resin/well) in fresh pre-wetted (3 × DCM, 3 × DMF) filtration microplates, and washed (2 × DMF). Fmoc was removed (20% piperidine in DMF, 1 × 5 min, 1 × 15 min, RT, 600 rpm), the resin was washed (3 × DMF, 3 × DCM, 3 × DMA, 200 µL), resuspended (DMA, 100 µL), incubated (30 min, RT, 600 rpm), and washed (DMA, 200 µL) prior to

the first building block coupling. Library synthesis proceeded in eight steps: acylation, Fmoc removal, encoding oligonucleotide ligation, resin pooling, resin splitting, acylation, encoding oligonucleotide ligation, and resin pooling.

DEL Amino Acid Coupling, Fmoc Removal

The first building block coupling consisted of acylation with an N-Fmoc-protected amino acid. Resin was resuspended (DMA, 0.15 mL) with N-Fmoc amino acid/HOAt/DIC (6/6/8.6 µmol, respectively). Plates were covered with adhesive foil and incubated (1 h, 37 °C, 600 rpm). Resin was washed (3 × DMA, 3 × DCM, 3 × DMF). Fmoc was removed (20% piperidine in DMF, 0.15 mL, 1 × 5 min, 1 × 15 min, RT, 600 rpm), and resin was washed (3 × DMF, 3 × DCM, 3 × DMF).

DEL Ligation of ≈13XX and ≈24XX Amino Acid Encoding Oligonucleotides

Resin was retrieved, washed (2 × BTPWB, 1 × 1X-BTPLB), resuspended (1X-BTPLB, 0.1 mL), and incubated (30 min, RT, 600 rpm). An encoding oligonucleotide ligation mixture containing T4 DNA ligase (4 mg/mL, 392 µL), 10X-BTPLB (8.7 mL), H_2O (48.9 mL) was prepared and added to all plate wells (0.1 mL) along with DI H_2O (24.8 µL). OP stocks of ≈13XX [±] (150 µM, 12.6 µL) and ≈24XX[±] (150 µM, 12.6 µL) were then added to the appropriate wells, the plate was sealed with adhesive foil, and incubated (4 h, RT, 600 rpm). Resin was washed (3 × BTPWB, 3 × 1:1 DMF:BTPWB, 3 × DMF), resuspended (DMF, 0.1 mL) and incubated (16 h, RT, 600 rpm). Resin was pooled into a reservoir, split into 6 × 96 wells (1.7 mg resin/well) of fresh pre-wetted (3 × DCM, 3 × DMF) filtration microplates, and washed (3 × DMF, 2 × DMA) prior to coupling the second building block set.

DEL Carboxylic Acid Coupling

The second building block coupling consisted of acylation with a carboxylic acid. Resin was resuspended (DMA, 0.15 mL) with carboxylic acid/Oxyma/TMP/DIC (12/12/12/17.1

μmol, respectively). Plates were covered with adhesive foil and incubated (3 h, 37 °C, 600 rpm). Resin was washed (3 × DMF, 3 × DCM, 3 × DMF, 3 × 1:1 DMF:BTPWB, 3 × BTPWB), resuspended (BTPWB, 0.1 mL), and incubated (overnight, RT, 600 rpm).

DEL Ligation of ≈15XX and ≈26XX Carboxylic Acid Encoding Oligonucleotides

Resin was washed (2 × BTPWB, 1 × 1X-BTPLB), resuspended (1X-BTPLB, 0.1 mL) and incubated (30 min, RT, 600 rpm). An encoding oligonucleotide ligation mixture containing T4 DNA ligase (4 mg/mL, 392 μL), 10X-BTPLB (8.7 mL), H_2O (48.9 mL) was prepared and aliquoted into all plate wells (0.1 mL) along with DI H_2O (24.8 μL). OP stocks of ≈15XX[±] (150 μM, 12.6 μL) and ≈26XX[±] (150 μM, 12.6 μL) were then added to the appropriate wells, the plate was sealed with adhesive foil, and incubated (4 h, RT, 600 rpm). Resin was washed (3 × BTPWB, 3 × 1:1 DMF:BTPWB, 3 × DMF), resuspended (DMF, 0.1 mL), incubated (16 h, RT, 600 rpm), and pooled. Synthesis resin and QC resin were separated by filtration (150 μm Celtrics, Sysmex, Lincolnshire, IL).

DEL Ligation of ≈0B02 Oligonucleotide

Ligation of ≈0B02[±] was performed on aliquots of DEL directly prior to screening. An aliquot of the DEL screening resin (10 mg) was transferred to a 1.5 mL tube, washed (3× BTPWB), and incubated (16 h, RT, 8 rpm). Resin was washed (3 × 1X-BTPLB), resuspended (1X-BTPLB, 1 mL), incubated (1 h, RT, 8 rpm), and the supernatant was removed. To resin, each of the following were added: 10X-BTPLB (150 μL), T4 ligase (4 mg/mL, 26 μL), ≈0B02[±] (60 μM, 1150 μL), and H_2O (174 μL). Resin was incubated (4 h, RT, 8 rpm) and washed (3 × BTPWB).

Solid-Phase DNA-Encoded Library QC: 10-μm Bead Analysis

The 10-μm library screening beads were subjected to qPCR analysis to quantitate the number of DNA tags per bead. An aliquot of 10-μm resin (0.06 mg, previously ligated

with closing primer) was transferred to a 1.5-mL tube, washed (BTPWB, 3 × 0.5 mL), and resuspended (BTPWB, 0.5 mL). The 10-µm bead concentration was determined by hemocytometer and diluted (100 beads/µL in BTPWB). Analysis by qPCR proceeded as previously described.[1,2]

The qPCR matrix for 10-µm beads contained Taq DNA polymerase (0.05 U/µL), oligonucleotide primers 5′-GCCGCCGCCTTCGTCCTTCTCAGCGAC-3′ and 5′-GTGGCACAAC AACTGGCGGGCAAAC-3′ (0.3 µM each), SYBR Green (0.2X), and GC-PCR buffer (1X). Library beads (10-µm, 100 beads/µL in BTPWB, 1 µL) were added to separate amplification wells containing qPCR matrix (20 µL, n = 10). Supernatant for each resin sample (1 µL) was added to separate amplification wells (20 µL, n = 2). Template standard solutions (1 fmol, 100 amol, 10 amol, 1 amol, 100 zmol, 10 zmol, 1 zmol, 100 ymol, and 10 ymol, each in 1 µL BTPWB) were added to separate amplification reactions (20 µL). Reactions were thermally cycled (96 °C, 10 s; [95 °C, 8s; 72 °C, 24 s] × 32 cycles; 72 °C, 2 min); with fluorescence monitoring (FAM channel, CFX96 Real-Time System, Bio-Rad) and Cq quantitated (CFX Manager, Version 3.1, Bio-Rad, baseline subtracted). The number of amplifiable tags per bead was calculated by dividing the qPCR result by the number of beads per well (bead count was confirmed using a stereo zoom microscope).

Solid-Phase DNA-Encoded Library QC: 160-µm Bead Analysis

The 160-µm QC beads were used for sequence-structure matching analysis. The qPCR matrix for 160-µm beads consisted of Taq DNA polymerase (0.05 U/µL), oligonucleotide primers 5′-CGCCAGGGTTTTCCCAGTCACGACCAACCACCCAAACCACAAACCC AAACCCCAAACCCAACACACAACAACAGCCGCCGCCTTCGTCCTTCTCAGCGA C-3′ (0.02 µM), 5′-CGCCAGGGTTTTCCCAGTCACGAC-3′ (0.28 µM), and 5′-GTGGCAC AACAACTGGCGGGCAAAC-3′ (0.3 µM), SYBR Green (0.2X), and GC-PCR buffer (1X).

Single 160-µm beads (1 µL, BTPWB) were added to individual wells with qPCR matrix (20 µL). Template standard solutions (1 fmol, 100 amol, 10 amol, 1 amol, 100 zmol, 10 zmol, 1 zmol, 100 ymol, and 10 ymol, each in 1 µL BTPWB) were added to separate amplification reactions (20 µL). Reactions were thermally cycled (95 °C, 2 min; [95 °C, 20s; 62 °C, 15 s; 72 °C, 20 s] × 24 cycles; 72 °C, 2 min); with fluorescence monitoring (FAM channel, CFX96 Real-Time System, Bio-Rad) and quantitated (CFX Manager, Version 3.1, Bio-Rad, baseline subtracted). The number of amplifiable tags per bead was calculated in reference to the template standards.

Single 160-µm resin beads were retrieved via pipet from PCR plate wells and deposited into a 96-well microplate (MeOH, 0.1 mL). Each 160-µm library bead PCR product (15 µL) was purified by native PAGE (6%, 1× TBE, 12 W, 30 min). Gel slices containing 221-nt DNA products were excised and eluted (C&S, 0.1 mL, 16 h, RT, 8 rpm). Eluates were analyzed by Sanger sequencing using the primer 5′-CGCCAGGGTTTTCCCAGTCACGAC-3′. Sequencing reads were trimmed to remove all called bases prior to the opening primer sequence (5′-GCCGCCCAGTCCTGCTCGCTTCGCTAC-3′). Sequences were aligned to a degenerate reference sequence (5′-ATGGNNNNNNNNTCANNNNNNNNGTTNNNNNNNNCTANNNNNNNNTTCNNNNNNNNCGCNNNNNNNNGCCTCCCAAACNNNNNNNNGTT-3′) and the encoding regions (5′-NNNNNNNN-3′) were matched to the building block alphanumeric identifier lookup table to assign the synthesis history for each compound.

Resin Cleavage and MALDI-TOF MS Analysis

Resin cleavage and MALDI-TOF MS analysis have previously been described.[1,2] Briefly, individual 160-µm beads (MeOH, 0.1 mL) were dried *in vacuo* (60 °C). Cleavage cocktail (90% TFA, 5% TIS, 5% DCM, 10 µL) was added to dried single 160-µm bead samples,

incubated (2 h, RT, 100 rpm), and dried *in vacuo* (60 °C). Compound was resuspended (50% ACN, 50% 0.1% TFA in H_2O, 6 µL), and a diluted (1:10) aliquot (1 µL) was co-spotted onto a MALDI-TOF MS target plate with HCCA matrix solution, dried, and analyzed via MALDI-TOF MS (Microflex, Bruker Daltronics, Inc., Billerica, MA, Table S3).

Microfluidic Device Fabrication

Master wafers were fabricated using soft photolithography as previously described.[4,5] Channel structures were fabricated in PDMS using soft lithography.[6] Degassed PDMS prepolymer (44 g, 10:1 base:curing agent) was poured over the master, cured (1 h, 80 °C), cooled (10 min, RT), and peeled from the master. Individual chips were removed from the surrounding PDMS using a razor blade. Fluidic ports were punched with a biopsy punch (0.75 mm dia., World Precision Instruments, Inc., Sarasota, FL). PDMS molds and glass slides (Corning Inc., Corning, NY) were cleaned with Safe-Soap (Gold Biotechnologies Inc., St. Louis, MO), rinsed with DI water, cleaned with isopropyl alcohol, and dried with compressed N_2 prior to plasma cleaning (200 mTorr, 90 s, Harrick Scientific, Pleasantville, NY). Glass slides and PDMS molds were then immediately bonded[6] (16 h, 80 °C). Microfluidic devices were fitted with integrated waveguides.[7] Waveguide illumination intensity was calibrated by pumping (0.5 µL/min) a solution of DAPI (50 µM) through the waveguide calibration channel while varying the LED's current. Calibrant solution fluorescence emission was measured in the 520 nm channel (100 Hz). Afterward, the calibration channel was rinsed with water, dried with air, and filled with trimethylsiloxy-terminated PDMS (DMS-T22, Gelest, Inc., Tullytown, PA).

Microfluidic Device Operation

Droplet generation oil (OIL1) was Novec-7500™ Engineered Fluid (3M, Saint Paul, MN) containing Pico-Surf™ (2.5% w/w, Sphere Fluidics, Cambridge UK). The droplet generation oil was prepared by combining Novec-7500™ with an equivolume of Pico-Surf™ (5% w/v in Novec-7500™) and mixing by pipette. The spacing (OIL2) and flow focusing oil (OIL3) were neat Novec-7500™.

AQ1, AQ2, OIL1, OIL2, and OIL3 were loaded into syringes (1/1/1/10/10 mL respectively, BD medical, Franklin Lakes, NJ) and fitted with blunt-tip Luer-Lok needles (0.15 mM ID, 30 G, Fisnar, Germantown, WI) and connected to fluidic inputs via microbore Tygon tubing (0.01″ x 0.03″ IC $\times$ OD, Saint-Gobain, Valley Forge, PA). Displacement syringe pumps (Legato 100, KD Scientific, Holliston, MA) drove fluids from syringes through the circuit. AQ1 and AQ2 were driven into the circuit, followed by OIL1 to initiate droplet generation. OIL2 and OIL3 were flowed (16 and 6 µL/min, respectively) to backfill the circuit. Flow rates for inputs library (AQ1, 0.4 µL/min), target (AQ2, 0.4 µL/min), OIL1 (0.5 µL/min), OIL2 (16 µL/min), and OIL3 (6 µL/min) were held constant. Droplet generation, splitting, and defaulting were confirmed by imaging with a high-speed camera (Integrated Design Technologies, Pasadena, CA). Flow was equilibrated (25 min) prior to data acquisition and screening.

Droplet-Scale Flow Injection Analysis of FXa Activity Assay Quality

FXa droplet assay buffer was used for all AQ inputs. For probe+buffer experiments, AQ1 contained FXa substrate (6 µM) and 5,6-carboxy TAMRA (2 µM) while AQ2 contained only buffer. For probe + FXa experiments, AQ1 contained FXa substrate (6 µM) and internal standard 5,6-carboxy TAMRA (2 µM), while AQ 2 contained FXa (90 nM). For probe + FXa

+ inhibitor experiments, AQ1 contained FXa substrate (6 µM), 5,6-carboxy TAMRA (2 µM), and gabexate mesylate (30 µM) while AQ2 contained Factor Xa (90 nM). Droplet data were acquired for 10 min (∼30,000 droplets) for all flow injection analysis conditions.

Droplet-Scale Flow Injection Analysis of ATX Activity Assay Quality

ATX droplet assay buffer was used for all AQ inputs. For probe-only experiments, AQ1 contained FS-3 substrate (10 µM) while AQ2 contained only buffer. For probe + ATX experiments, AQ1 contained FS-3 substrate (10 µM) while AQ2 contained ATX (100 nM). Droplet data were acquired for 30 min (∼90,000 droplets) for all flow injection analysis conditions.

FXa Library Screening in Droplets

DEL screening for FXa inhibitors was performed in droplets as previously described,[4,8] with modifications. After ligating the closing primer, the library was prepared for droplet screening by exchanging into assay buffer, filtering through a 20 µm Celtrics filter, filtering through a previously described microfluidic device,[4] and counting a dilution with a hemocytometer to determine bead density. AQ1 input contained fluorogenic FXa peptide substrate (6 µM), internal standard 5(6)-TAMRA (2 µM), DEL beads (∼1500 beads/µL), and a PTFE-encapsulated magnet (to agitate beads periodically; 3 mm dia., V&P Scientific, Inc., San Diego, CA) in FXa droplet assay buffer. AQ2 contained FXa (3.8 ng/µL, ∼90 nM) in FXa droplet assay buffer. OIL1 was Novec™ 7500 containing Pico-Surf™(2.5% w/v). OIL2 and OIL3 were neat Novec™ 7500.

UV dosing was delivered through a custom fiber optic cable[7] controlled through Labview software (National Instruments, Austin, TX). Slight modifications were made to the previously described confocal LIF detection system and LabVIEW code.[3,4] Briefly, droplet

fluorescence emission was detected by photon counting PMTs (H7828, Hamamatsu, Middlesex, NJ) using a custom two-channel (520 and 570 nm) confocal LIF microscope with laser excitation (488 nm, 10 mW, OBIS-488 20LS, Coherent Inc., Santa Clara, CA). LabVIEW code written in house collected signal data and determines droplet sorting decisions. The PMT signals were digitized by a data acquisition board (DAQ, NI USB-6341, National Instruments), and binned into packets of counts (Δt = 0.1 ms). Median-filter smoothing (window width = 3) was applied to the signal in real-time. Droplet signal regions were identified by the 570-nm channel signal ($\text{counts}_n > 200$) and 520-nm channel signal maxima ($\text{max} = \text{counts}_{n-1}$ when $\text{counts}_n < \text{counts}_{n-1}$).

A dynamic sorting threshold was calculated in real-time to identify "hit droplets" with reduced 520-nm channel fluorescence. The dynamic threshold was calculated as µ - 4.5σ, where µ and σ were the mean and standard deviation of the last 1000 droplets' fluorescence values, respectively. Hit droplets were excluded from the fluorescence values used to calculate population mean and standard deviation. When a hit droplet was detected (droplet fluorescence < µ – 4.5σ counts), LabVIEW output a TTL pulse from the DAQ board to a waveform generator (Agilent 33210A, Agilent Technologies, Santa Clara, CA), triggering a square wave pulse output (0–7.5 V, 10 kHz, 300 cycles) that was amplified (gain = 100 V/V, TREK Model 2210 high-voltage power amplifier, TREK Inc., Lockport, NY) and conducted through luer-lok needle-fitted and microbore tygon tubing filled with liquid metal (gallium:indium:tin alloy 62:22:16 (w%), Alfa Aeser) into a microfabricated electrode channel (V_{AC}).

ATX Library Screening in Droplets

The ATX droplet screening assay was performed similarly to the FXa assay, but with ATX assay reagents. This was a modification of previously described protocols.[4] AQ1 input contained fluorogenic ATX substrate FS-3[9] (10 µM), internal standard 5(6)-TAMRA (5 µM),

DEL beads (~1500 beads/µL), and a PTFE-encapsulated magnet in ATX droplet assay buffer. AQ2 contained ATX (100 nM) in ATX droplet assay buffer. OIL1 was Novec™ 7500 containing 2.5% Pico-Surf™. OIL2 and OIL3 were neat Novec™ 7500. The dynamic threshold was calculated as µ - 6σ.

Synthesis of Photocleavable Fluorescein Beads (PC-Gly-FAM Beads)

Photocleavable glycine-fluorescein (PC-Gly-FAM) beads were synthesized on a photolabile linker to mimic DEL synthesis procedures. All fritted-syringe wash volumes were identical (5.0 mL). Quality-control (QC) TentaGel rink amide resin (200 µm, 0.325 mmol/g, 200 mg, Rapp-Polymere) was transferred to a fritted syringe (25 mL, Torviq), swelled in solvent (DMF, 1 h, RT, 8 rpm), and washed (3 × DMF). Fmoc was removed (20% piperidine in DMF, 5 mL, 1 × 5 min, 1 × 15 min, RT, 8 rpm) and the resin was washed (3 × DMF, 3 × DCM, 3 × DMA). N-α-Fmoc-K(Mca)-OH (325 µmol) was activated with DIC/HOAt/DIEA (325/325/650 µmol) in DMA (2.1 mL, 2 min, RT), added to the resin, incubated (2 h, 50 °C, 8 rpm), and the resin was washed (3 × DMA, 3 × DCM, 3 × DMA). Unreacted amines on the resin were capped with acetic anhydride (5 mL, 20% in DMA, 30 min, 50 °C, 8 rpm), and the resin was washed (3 × DMA, 3 × DCM, 3 × DMA). All future acetic anhydride capping and resin washing proceeded identically. Fmoc was removed (20% piperidine in DMF, 5 mL, 1 × 5 min, 1 × 15 min, RT, 8 rpm) and the resin was washed (3 × DMF, 3 × DCM, 3 × DMF). N-α-Fmoc-R(Pbf)-OH (650 µmol) was activated with DIC/HOAt/DIEA (650/650/1300 µmol) in DMA (3.9 mL, 2 min, RT), added to resin, incubated (1 h, 50 °C, 8 rpm), and the resin was washed (3 × DMF, 3 × DCM, 3 × DMF).

Synthesis resin (amino-functionalized, 10 µm dia., 0.29 mmol/g, 200 mg, Rapp-Polymere) and the aforementioned QC resin were transferred to a syringe (25 mL, Torviq) fitted with two additional frits (10-µm polyethylene, 20 mm dia., Biotage ISOSOLUTE). All fritted-syringe wash volumes were identical (10.0 mL). Pooled resin was swelled in sol-

vent (DMF, 16 h, RT, 8 rpm), and washed (3 × DMF, 3 × DCM, 3 × DMF). Fmoc was removed (20% 4-methylpiperidine in DMF, 1 × 5 min, 1 × 15 min, RT, 8 rpm), and resin was washed (3 × DMF, 3 × DCM, 3 × DMF). N-α-Fmoc-Gly-OH (1.2 mmol) was activated with DIC/HOAt/DIEA (1.2/1.2/2.4 mmol) in DMA (8.2 mL, 2 min, RT), added to resin, and incubated (1 h, 50 °C, 8 rpm, 2 ×). Unreacted amines on the resin were capped with acetic anhydride and the resin was washed. Fmoc was removed (20% 4-methylpiperidine in DMF, 1 × 5 min, 1 × 15 min, RT, 8 rpm), and resin was washed (3 × DMF, 3 × DCM, 3 × DMF).

N-Fmoc-L-propargylglycine-OH (1.2 mmol) was activated with DIC/HOAt/DIEA (1.2/1.2/2.4 mmol) in DMA (8.2 mL, 2 min, RT), added to resin, and incubated (1 h, 50 °C, 8 rpm, 2 ×). Unreacted amines on the resin were capped with acetic anhydride, and the resin was washed. Fmoc was removed (20% 4-methylpiperidine in DMF, 1 × 5 min, 1 × 15 min, RT, 8 rpm), and resin was washed (3 × DMF, 3 × DCM, 3 × DMF). N-α-Fmoc-Gly-OH (1.2 mmol) was activated with DIC/HOAt/DIEA (1.2/1.2/2.4 mmol) in DMA (8.2 mL, 2 min, RT), added to resin, and incubated (1 h, 50 °C, 8 rpm, 2 ×). Unreacted amines on the resin were capped with acetic anhydride and the resin was washed.

Dual-scale resin (33 µmol of 200 µm beads, 60 µmol of 10 µm beads) was allocated for continued synthesis. Fmoc was removed (20% 4-methylpiperidine in DMF, 1 × 5 min, 1 × 15 min, RT, 8 rpm), and resin was washed (3 × DMF, 3 × DCM, 3 × DMF). All subsequent synthesis steps occured in a UV-free room. Fmoc-PC-OH (310 µmol) was activated with DIC/Oxyma/TMP (465/310/310 µmol) in DMA (8.2 mL, 2 min, RT), added to resin, incubated (1 $times$ 3 h, 1 × 16 h, 50 °C, 8 rpm), and the resin was washed (3 × DMF, 3 × DCM, 3 × DMF).

N-α-Fmoc-glycine and 5-CarboxyFluorescein (5-FAM) were coupled to photolinker fol-

lowing library synthesis conditions. Pooled dual-scale resin (16 µmol of 200 µm beads, and 30 µmol of 10 µm beads) was transferred to a new 25 mL syringe with additional frits for continued synthesis. Fmoc was removed (20% 4-methylpiperidine in DMF, 1 × 5 min, 1 × 15 min, RT, 8 rpm), and resin was washed (3 × DCM, 3 × DMF). N-α-Fmoc-Gly-OH (160 µmol) was activated with DIC/HOAt (228/160 µmol) in DMA (4 mL, 2 min, RT), added to resin, and incubated (1 h, 37 °C, 8 rpm). The resin was washed (3 × DCM, 3 × DMF). Fmoc was removed (20% 4-methylpiperidine in DMF, 1 × 5 min, 1 × 15 min, RT, 8 rpm), and resin was washed (3 × DCM, 3 × DMF). 5-FAM (Cayman Chemicals) was activated with DIC/Oxyma/TMP (320/320/400 µmol) in DMA (4 mL, 2 min, RT), added to resin, and incubated (3 h, 50 °C, 8 rpm). Resin was washed (3 × DCM, 3 × DMF).

Resin (< 1 mg) was removed for MADLI-TOF MS analysis. Resin was washed (3 × DCM), and dried over a vacuum manifold. Beads were incubated in cleavage cocktail (300 µL 95/2.5/2.5 TFA/TIS/H_2O, 1h, 8 rpm), and the solution was isolated from the resin. The cleavage solution was dried *in vacuo*, resuspended (50 µL ACN/0.1% TFA H_2O), and diluted (1:100 in 50 % ACN/50% 0.1% TFA H_2O). The diluted cleavage product (1 µL) was was co-spotted onto a MALDI-TOF MS target plate with HCCA matrix solution, dried, and analyzed via MALDI-TOF MS (Microflex, Bruker Daltronics, Inc., Billerica, MA, Figure S14).

Measurement of Fluorescein in Droplets Following Bead Photocleavage.

The same microfluidic device used for DEL screeing was used to assess the concentration of fluorescein-glycine-NH_2 after photocleavage. AQ1-A input contained PC-Gly-FAM beads and a PTFE-encapsulated magnet in FXa droplet assay buffer. AQ1-B input contained 500 µM 5-FAM in FXa droplet assay buffer. AQ2 contained 500 µM 5(6)-CarboxyTamra in FXa assay buffer as an internal standard. OIL1 was Novec™ 7500 containing 2.5% Pico-Surf™. OIL2 and OIL3 were neat Novec™ 7500. Input flow rates recapitulated DEL screening

conditions. Droplets were generated to contain beads and internal standard, then the chip was backfilled with OIL2 and OIL3. Droplets were exposed to 4%, 10%, 20%, and 100% UV doses for $\sim$ 20 min at each dose with intervening 0% UV doses. All droplets were detected in the PMT2 channel which recorded TAMRA emission. Beaded droplets with cleaved fluorescein-glycine-NH_2 were detected in PMT1. Droplet data were filtered to exclude droplets without beads (PMT1 $<$ 10 RFU). Droplet data were further filtered to include only singly beaded droplets by selecting the most prevalent population (Figure S4). Following bead photocleavage assessment, AQ1-A was replaced with AQ1-B to generate droplets of known [5-FAM]. The flow rate of AQ1-B was varied from 600, 400, 200, and 100 nL/min while AQ2 was adjusted to maintain a constant 800 nL/min combined aqueous flow rate. This provided droplets of [5-FAM] = 325, 250, 125, and 62.5 µM, which were used to generate a linear standard curve and quantitate fluorescein-glycine-NH_2 photocleaved from beads into droplets.

Hit Bead Isolation

Hit beads were isolated from the droplet/oil matrix by filtration on a Costar Spin-X filter (0.45 µm, cellulose acetate, Corning). Filtration steps were performed by applying solution to the filter and centrifuging on a benchtop centrifuge (2690 $\times$g, 45 s) until no liquid remained. Filters were pre-equilibrated with perfluorooctanol:MeOH:tween 80 (17:83:0.03). Hit droplet/carrier oil matrix was applied to the filter and centrifuged to remove liquid. Hit collection tubes were washed with perfluorooctanol:MeOH:Tween 80 (17:83:0.03; 2 x 300 µL), MeOH:Tween 80 (100:0.04 ; 2 $\times$ 300 µL), BTPWB (2 $\times$ 300 µL) with intervening centrifugation steps (2690 $\times$g, 45 s). Hit beads were recovered from the filter by repeatedly pipetting and removing BTPWB (4 $\times$ 200 µL). Collected bead solutions were centrifuged, and the majority of supernatant was removed before transferring the beads in solution (10 µL BTPWB) to a 0.2 mL PCR tube.

Hit Bead Amplification and Preparation for Illumina High-Throughput Sequencing

Sequences from hit beads were amplified while sequencing barcodes and Illumina adapters (P5, P7) were introduced by PCR. The same forward primer (5′-AATGATACGGCGAC CACCGAGATCTACACTCTTTCCCTACACGACGCTCTTCCGATCTGCCGCCGCCT TCGTCCTTCTCAGCGAC-3′) was used for all amplification reactions and installed the P5 adapter for Illumina sequencing. Individual hit bead collections corresponding to a particular screening day and UV dose were amplified in individual reactions with a unique reverse primer (5′-CAAGCAGAAGACGGCATACGAGATXXXXXXGTGACTGGAGT TCAGACGTGTGCTCTTCCGATCTGTGGCACAACAACTGGCGGGCAAAC-3′). The reverse primer installed the P7 adapter and a unique barcode (5′-XXXXXX-3′) to identify each screening set during pooled sequencing (Table S5).

PCR mix consisted of isolated hit beads (∼100–700 in 10 µL BTPWB) and GC-PCR buffer (40 µL). Template standard solutions (1 fmol, 100 amol, 10 amol, 1 amol, each in 10 µL BTPWB) were added to separate amplification reaction wells (40 µL). Wells containing PCR mix (40 µL) and hit bead collections (10 µL, ∼100-700 beads) were thermally cycled (96 °C, 120 s; [95 °C, 20 s; 63 °C, 15 s;69 °C, 20 s] × 32; 72 °C, 120 s) with fluorescence monitoring (FAM channel, CFX96 Real-Time System, Bio-Rad), and the reaction was manually stopped when the amplification signal from the hit bead-containing wells began to plateau.

DNA Sequencing and Data Processing

Barcoded PCR amplification products were pooled, resolved by native PAGE (6%, 19:1 acrylamide/bisacrylamide, 1 × TBE, 200 V, 30 min), and visualized with SYBR Gold staining (Life Technologies, Inc.). Gel slices that contained 264-bp DNA product were

excised, eluted (C&S buffer, 100 µL, 100 rpm, ON), and pooled for Illumina sequencing at the UCI genomics high throughput facility (MiSeq v2 Nano or MiSeq v2 Micro, paired end). DNA sequence pattern matching, aggregation by bead barcode, and unique molecular identifier (UMI) filtering were previously described.[3–5] Sequences with a UMI count ≤ 5 were discarded. UV doses associated with hit sequences were filtered using the sequencing barcode (Table S5) introduced during library amplification. Of the remaining sequences, compound replicates (*k* class) were determined by taking the sum of the sequences having the same synthesis encoding region but distinct bead barcodes.

Biochemical Assay for FXa Hit Validation

Compound stocks (50–100 mM in DMSO-d6) were serially diluted (1:3.16 in FXA microwell-plate assay buffer). Compound dilutions (2.5 µL) were added to FXa (90 nM, 5 µL) in 384-well black-bottom microplates (Corning Scientific, Corning, NY) in triplicate and incubated (30 min, 25 °C). 5-FAM/QXL™-520 Factor Xa substrate (12 µM, 2.5 µL) was added and reaction progress was monitored immediately (λ_{ex} = 483 nm, λ_{em} = 530 nm, 25 °C, CLARIOStar Plus, BMG LABTECH, Ortenberg, Germany). The percent activity was taken as the slope of the fluorescence increase over time (1–10 min) relative to that of a vehicle control. IC_{50} values were determined by fitting the data to a 4 parameter logistic model using nonlinear regression.

Biochemical Assay for ATX Hit Validation

Compound stocks (25–50 mM in DMSO-d6) were serially diluted (1:3.16 in ATX microwell-plate assay buffer). Compound dilutions (2.5 µL) were added to ATX (100 nM, 5 µL) in 384-well black-bottom microplates (Corning Scientific, Corning, NY) in triplicate and incubated (30 min, 25 °C). FS-3 (20 µM, 2.5 µL) was added and reaction progress was monitored immediately (λ_{ex} = 483 nm, λ_{em} = 530 nm, 25 °C, CLARIOStar Plus, BMG LABTECH). The percent activity was taken as the slope of the fluorescence increase over

time (1–10 min) relative to that of a vehicle control. IC_{50} values were determined by fitting the data to a 4 parameter logistic model using nonlinear regression.

Modeling ATX Competitive Inhibition

ATX inhibition was modeled using the Michaelis-Menten steady-state approximation for competitive inhibition:

$$v = \frac{V_{max}[S]}{K_M(1 + \frac{[I]}{K_i}) + [S]} \tag{1}$$

where v is the observed reaction velocity, V_{max} is the maximum reaction velocity, $[S]$ is the substrate concentration, K_M is the Michaelis constant, $[I]$ is the inhibitor concentration and K_i is the inhibition constant. Rearranging to express relative velocity (v/V_{max}):

$$\frac{v}{V_{max}} = \frac{[S]}{K_M(1 + \frac{[I]}{K_i}) + [S]}. \tag{2}$$

Fixing $[S]$ and K_M at 5 µM and 1.1 µM,[10] respectively, relative velocity was calculated for a range of $[I]$ (0.1–100 µM) and a set of K_i values (0.1, 0.3, 1, 3, 10, 30, and 100 µM). Relative velocity was normalized to the maximum possible ([I]=0) to give ATX activity (%).

Modeling ATX Noncompetitive Inhibition

ATX inhibition was modeled using the Michaelis-Menten steady-state approximation for noncompetitive inhibition:

$$v = \frac{V_{max}}{(1 + [I]/K_i)}[S]. \tag{3}$$

Rearranging to express relative velocity (v/V_{max}):

$$\frac{v}{V_{max}} = \frac{[S]}{(K_M + [S])(1 + [I]/K_i)}. \tag{4}$$

Fixing $[S]$ and K_M at 5 µM and 1.1 µM,[10] respectively, relative velocity was calculated for a range of $[I]$ (0.1–100 µM) and a set of K_i values (0.1, 0.3, 1, 3, 10, 30, and 100 µM). Relative velocity was normalized to the maximum possible ([I]=0) to give ATX activity (%).

Modeling ATX Uncompetitive Inhibition

ATX inhibition was modeled using the Michaelis-Menten steady-state approximation for noncompetitive inhibition:

$$v = \frac{\frac{V_{max}}{(1+[I]/K_i)}[S]}{\frac{K_M}{(1+[I]/K_i)} + [S]}. \tag{5}$$

Rearranging to express relative velocity (v/V_{max}):

$$\frac{v}{V_{max}} = \frac{\frac{1}{(1+[I]/K_i)}[S]}{\frac{K_M}{(1+[I]/K_i)} + [S]}. \tag{6}$$

Fixing $[S]$ and K_M at 5 µM and 1.1 µM,[10] respectively, relative velocity was calculated for a range of $[I]$ (0.1–100 µM) and a set of K_i values (0.1, 0.3, 1, 3, 10, 30, and 100 µM). Relative velocity was normalized to the maximum possible ([I]=0) to give ATX activity (%).

Compound Synthesis and Characterization

All compounds were characterized by NMR and verified $> 95\%$ purity by LC-MS.

NMR Analysis

^{1}H NMR and ^{13}C NMR spectra (Ascend 400, Bruker) are reported relative to TMS using residual solvent signals as an internal reference.

LC-MS Analysis

Reaction products were separated with gradient elution (mobile phase A 0.05% formic acid in H_2O; mobile phase B MeOH; 5–95% B, 8 min), at constant flow rate (0.4 mL/min) using a C18 column (Gemini NX-C18, 3 µm, 110 Å, 50 × 2 mm, Phenomenex, Torrance, CA), ionized via heated electrospray, and mass analyzed using a single quadrupole mass spectrometer (ISQ EM, Thermo Scientific, Waltham MS). Samples were analyzed with absorbance detection (λ = 214, 280, 365, 488 nm) and total ion current (positive ionization mode, 150–2000 m/z).

Semi-Preparative HPLC Purification

Crude reaction mixtures were purified by HPLC using gradient elution (mobile phase A 0.1% TFA in H_2O; mobile phase B MeOH; from between 10–30% B to 75% B over 16 min) at constant flow rate (5 mL/min) using a C18 column (Pursuit 5 µm, 150 mm x 10.0 mm, 200 Å, Agilent). Fractions containing the intended product were pooled, frozen, and dried by lyophilization. Resulting compound purity was determined by LC-MS analysis.

Synthesis of 1a: (R)-N-(1-amino-3-(benzo[b]thiophen-3-yl)-1-oxopropan-2-yl)-4-(2-oxopyrrolidin-1-yl)benzamide

Rink amide MBHA resin (121.1 mg, 48.4 µmol sites, Anaspec) was added to a fritted syringe (6 mL, Torviq) and swelled in DMF (3 mL, 3 h, RT, 8 rpm). Fmoc was removed (3 mL 20% 4-methylpiperidine v/v in DMF, 10 min, RT, 8 rpm, 2×), and the resin was washed (2 × DMF, 2 × DCM, 2 × DMF, 2 mL/wash). Fmoc-3-benzothienyl-D-alanine (106.5 mg, 240 µmol, 5 eq, 120 mM) was activated with DIC (41.4 µL, 265 µmol, 5.4 eq, 130 mM), HOAt (33.4 mg, 245 µmol, 5.1 eq, 120 mM), and DIEA (92 µL, 524 µmol, 10.8 eq, 262 mM) dissolved in DMF (2 mL). Activated amino acid was added to resin and incubated (3 h, RT, 8 rpm). The resin was then washed (2 × DMF, 2 × DCM, 2 × DMF, 2 mL/wash). Fmoc was removed (3 mL 20% 4-methylpiperidine v/v in DMF, 10 min, RT, 8 rpm, 2×), and the resin was washed (2 × DMF, 2 × DCM, 2 × DMF, 2 mL/ wash). 4-(2-Oxo-pyrrolidin-1-yl)-benzoic acid (48.9 mg, 238 µmol, 4.9 eq, 120 mM) was activated with DIC (41.4 µL, 265 µmol, 5.4 eq, 130 mM), HOAt (34.9 mg, 256 µmol, 5.3 eq, 130 mM), and DIEA (92 µL, 524 µmol, 10.8 eq, 262 mM) dissolved in DMF (2 mL). Activated carboxylic acid was added to resin and incubated (3 h, RT, 8 rpm). The resin was then washed (2 × DMF, 2 × DCM, 2 × DMF, 3 × DCM; 2 mL/ wash) and dried *in vacuo*. Dried resin was suspended in cleavage solution (95/2.5/2.5 TFA/TIS/H2O, 4.2 mL) and incubated (4 h, RT, 8 rpm). Cleavage solution was expelled and collected. Resin was washed with 10 mL DCM and collected. Collected solution was evaporated *in vacuo*. Crude product was dissolved (2.5 mL, 1:2:2 DMSO:MeCN:0.1% TFA/water) and purified by semi-preparative reverse phase HPLC. Fractions containing the intended product (as determined by MS analysis) were pooled, frozen, and evaporated by lyophilization, yielding a white solid (13.1 mg, 32.1 µmol, 66% yield).

^{1}H NMR (DMSO-d6, 400 MHz): δH 8.48 (d, 1H, J = 8.4 Hz), 7.99 (d, 1H, J = 7.8 Hz), 7.91 (d, 1H, J = 7.9 Hz), 7.80 (m, 2H), 7.68 (m, 2H), 7.61 (s, 1H), 7.48 (s, 1H), 7.39 (t, 1H, J = 7.4 Hz), 7.32 (t, 1H, J = 7.4 Hz), 7.15 (s, 1H), 4.76 (m, 1H), 3.81 (t, 2H, J = 7.1 Hz), 3.28 (m, 2H), 2.47 (m, 2H), 2.02 (qt, 2H, J = 7.5 Hz).

^{13}C NMR(DMSO-d6, 100 MHz): δC 174.3, 173.3, 165.6, 142.0, 139.5, 138.7, 132.7, 128.8, 128.0, 124.2, 123.7, 122.9, 121.9, 118.1, 52.8, 48.0, 32.4, 30.4, 17.3.

Synthesis of 1b: (R)-N-(1-amino-3-(benzo[b]thiophen-3-yl)-1-oxopropan-2-yl)-4-morpholinobenzamide

Sieber amide resin (96.4 mg, 58.8 µmol sites, Anaspec) was added to a fritted syringe (6 mL, Torviq) and swelled in DMF (3 mL, 16 h, RT, 8 rpm). Fmoc was removed (3 mL 20% 4-methylpiperidine v/v in DMF, 10 min, RT, 8 rpm, 2×), and the resin was washed (2 × DMF, 2 × DCM, 2 × DMF, 2 mL/wash). Fmoc-3-benzothienyl-D-alanine (133.6 mg, 300 µmol, 5.1 eq, 150 mM) was activated with DIC (50 µL, 320 µmol, 5.4 eq, 160 mM), HOAt (41.0 mg, 300 µmol, 5.1 eq, 150 mM), and DIEA (110 µL, 630 µmol, 10.7 eq, 320 mM) dissolved in DMA (2 mL). Activated amino acid was added to resin, incubated (5 h, RT, 8 rpm), and the resin was washed (2 × DMF, 2 × DCM, 2 × DMF, 2 mL/wash). Fmoc was removed (3 mL 20% 4-methylpiperidine v/v in DMF, 10 min, RT, 8 rpm, 2×), and the resin was washed (2 × DMF, 2 × DCM, 2 × DMF, 2 mL/ wash). 4-(4-Morpholinyl)benzoic acid (62.0 mg, 300 µmol, 5.1 eq, 190 mM) was activated with DIC (50 µL, 320 µmol, 5.4 eq, 200 mM), HOAt (41.0 mg, 300 µmol, 5.1 eq, 190 mM), and DIEA (110 µL, 630 µmol, 10.7 eq, 390 mM) dissolved in DMA (1.6 mL). Activated carboxylic acid was added to resin and incubated (2.5 h, RT, 8 rpm). The resin was then washed (2 × DMF, 2 × DCM, 2 × DMF, 3 × DCM; 2 mL/ wash) and dried *in vacuo*. Dried resin was suspended in cleavage solution (4 mL, 2.5% TFA in DCM) and incubated (2 h, RT, 8 rpm). Cleavage solution was expelled

and collected. Resin was washed with 10 mL DCM and collected. Collected solution was evaporated *in vacuo*. Crude product was dissolved (6 mL, 3:1:2 DMSO:MeOH:0.1% TFA/water) and purified by semi-preparative reverse phase HPLC. Fractions containing the intended product (as determined by MS analysis) were pooled, frozen, and evaporated by lyophilization, yielding a white solid (20.4 mg, 50 µmol, 85% yield).

^{1}H NMR (DMSO-d6, 400 MHz): δH 8.24 (d, 1H, J = 8.2 Hz), 7.97 (d, 1H, J = 7.8 Hz), 7.89 (d, 1H, J = 7.8 Hz), 7.68 (m, 2H), 7.55 (s, 1H), 7.46 (s, 1H), 7.38 (t, 1H, J = 7.0 Hz), 7.31 (t, 1H, J = 7.0 Hz), 7.11 (s, 1H), 6.89 (m, 2H), 4.74 (m, 1H), 3.68 (t, 4H, J = 4.8 Hz), 3.26 (m, 2H), 3.14 (t, 4H, J = 4.8 Hz).

^{13}C NMR(DMSO-d6, 100 MHz): δC 173.6, 165.9, 153.0, 139.5, 138.7, 132,8, 128.7, 124.2, 123.0, 123.6, 122.8, 121.9, 113.2, 65.9, 52.7, 47.4, 30.5.

Synthesis of 2a: (R)-N-(1-amino-3-(1H-indol-3-yl)-1-oxopropan-2-yl)-4-(2-oxopyrrolidin-1-yl)benzamide

Sieber amide resin (100.5 mg, 61.3 µmol sites, Anaspec) was added to a fritted syringe (6 mL, Torviq) and swelled in DMF (3 mL, 16 h, RT, 8 rpm). Fmoc was removed (3 mL 20% 4-methylpiperidine v/v in DMF, 10 min, RT, 8 rpm, 2×), and the resin was washed (2 × DMF, 2 × DCM, 2 × DMF, 2 mL/wash). Fmoc-D-tryptophan (136.5 mg, 320 µmol, 5.2 eq, 160 mM) was activated with DIC (50 µL, 320 µmol, 5.2 eq, 160 mM), HOAt (43.4 mg, 320 µmol, 5.2 eq, 160 mM), and DIEA (110 µL, 630 µmol, 10.3 eq, 320 mM) dissolved in DMA (2 mL). Activated amino acid was added to resin and incubated (5 h, RT, 8 rpm). The resin was then washed (2 × DMF, 2 × DCM, 2 × DMF, 2 mL/wash). Fmoc was removed (3 mL 20% 4-methylpiperidine v/v in DMF, 10 min, RT, 8 rpm, 2×), and the resin was washed (2 × DMF, 2 × DCM, 2 × DMF, 2 mL/ wash). 4-(2-Oxo-pyrrolidin-1-yl)-benzoic acid (61.8 mg, 300 µmol, 4.9 eq, 190 mM) was activated with DIC (50 µL, 320 µmol, 5.2 eq,

200 mM), HOAt (44.2 mg, 320 µmol, 5.2 eq, 200 mM), and DIEA (110 µL, 630 µmol, 10.3 eq, 390 mM) dissolved in DMA (1.6 mL). Activated carboxylic acid was added to resin and incubated (2.5 h, RT, 8 rpm). The resin was then washed (2 × DMF, 2 × DCM, 2 × DMF, 3 × DCM; 2 mL/ wash) and dried *in vacuo*. Dried resin was suspended in cleavage solution (4 mL, 2.5% TFA in DCM) and incubated (2 h, RT, 8 rpm). Cleavage solution was expelled and collected. Resin was washed with 10 mL DCM and collected. Collected solution was evaporated *in vacuo*. Crude product was dissolved (3 mL, 1:1 DMSO:0.1% TFA/water) and purified by semi-preparative reverse phase HPLC. Fractions containing the intended product (as determined by MS analysis) were pooled, frozen, and evaporated by lyophilization, yielding a white solid (20.4 mg, 50 µmol, 85% yield).

^{1}H NMR (DMSO-d6, 400 MHz): δH 10.73 (s, 1H), 7.80 (m, 2H), 7.68 (m, 2H), 7.64 (s, 1H), 7.53 (s, 1H), 7.26 (d, 1H, J = 8.2 Hz), 7.16 (d, 1H, J = 2.2 Hz), 7.06 (s, 1H), 7.01 (t, 1H, J = 7.0 Hz), 6.93 (t, 1H, J = 7.0 Hz), 4.64 (m, 1H), 3.80 (t, 2H, J = 7.1 Hz), 3.14 (m, 2H), 2.46 (m, 2H), 2.01 (qt, 2H, J = 7.5 Hz).

^{13}C NMR(DMSO-d6, 100 MHz): δC 174.3, 173.9, 165.5, 142.0, 136.1, 129.0, 128.1, 127.3, 123.6, 120.9, 118.5, 118.2, 118.1, 111.3, 110.7, 54.1, 48.0, 32.5, 27.6, 17.3.

Synthesis of 3a: 1-(1-(6-chloroimidazo[1,2-a]pyridine-2-carbonyl)piperidin-4-yl)pyrrolidine-2-carboxamide

Sieber amide resin (100.5 mg 61.3 µmol sites, Anaspec) was added to a fritted syringe (6 mL, Torviq) and swelled in DMF (3 mL, 16 h, RT, 8 rpm). Fmoc was removed (3 mL 20% 4-methylpiperidine v/v in DMF, 10 min, RT, 8 rpm, 2×), and the resin was washed (2 × DMF, 2 × DCM, 2 × DMF, 2 mL/wash). Fmoc-4-piperidino-L-proline acid (136.5 mg, 310 µmol, 5.1 eq, 160 mM) was activated with DIC (50 µL, 320 µmol, 5.2 eq, 160 mM), HOAt (42.0 mg, 310 µmol, 5.1 eq, 160 mM), and DIEA (110 µL, 630 µmol, 10.3 eq, 320 mM)

dissolved in DMA (2 mL). Activated amino acid was added to resin and incubated (5 h, RT, 8 rpm). The resin was then washed (2 × DMF, 2 × DCM, 2 × DMF, 2 mL/wash). Fmoc was removed (3 mL 20% 4-methylpiperidine v/v in DMF, 10 min, RT, 8 rpm, 2×), and the resin was washed (2 × DMF, 2 × DCM, 2 × DMF, 2 mL/ wash). 6-Chloroimidazo[1,2-a]pyridine-2-carboxylic acid (60.8 mg, 310 µmol, 5.1 eq, 190 mM) was activated with DIC (50 µL, 320 µmol, 5.2 eq, 200 mM), HOAt (43.1 mg, 320 µmol, 5.2 eq, 200 mM), and DIEA (110 µL, 630 µmol, 10.3 eq, 390 mM) dissolved in DMA (1.6 mL). Activated carboxylic acid was added to resin and incubated (2.5 h, RT, 8 rpm). The resin was then washed (2 × DMF, 2 × DCM, 2 × DMF, 3 × DCM; 2 mL/ wash) and dried *in vacuo*. Dried resin was suspended in cleavage solution (4 mL, 2.5% TFA in DCM) and incubated (2 h, RT, 8 rpm). Cleavage solution was expelled and collected. Resin was washed with 10 mL DCM and collected. Resin was washed with 10 mL 50/50 MeOH/water and collected. Collected solution was evaporated *in vacuo*. Crude product was dissolved (3 mL, 1:1 DMSO:0.1% TFA/water) and purified by semi-preparative reverse phase HPLC. Fractions containing the intended product (as determined by MS analysis) were pooled, frozen, and evaporated by lyophilization, yielding a white solid (4.2 mg, 11.2 µmol, 20% yield).

^{1}H NMR (DMSO-d6, 400 MHz): δH 8.87 (s, 1H), 8.31 (s, 1H), 8.08 (s, 1H), 7.68 (d, 1H, J = 9.6 Hz), 7.40 (dd, 1H, J = 11.6, 2.0 Hz), 5.31 (br, 1H), 4.61 (br, 1H), 4.26 (br, 1H), 3.28 (br, 2H), 3.09 (m, 2H), 2.75 (m, 1H), 2.40 (m, 1 H), 2.00 (m, 4H), 1.26 (m, 2H), 1.51 (m,1H).

^{13}C NMR(DMSO-d6, 100 MHz): δC 170.1, 161.8, 141.7, 140.6, 127.2, 125.3, 120.1, 118.4, 117.6, 64.3, 61.7, 52.6, 44.2, 30.0, 23.3, 22.3.

Synthesis of 5a: N-(3-carbamoylbenzyl)-5-chlorothiophene-2-carboxamide

Rink amide MBHA resin (133.9 mg 58 µmol sites, Anaspec) was added to a fritted syringe (6 mL, Torviq) and swelled in DMF (3 mL, 3 h, RT, 8 rpm). Fmoc was removed (3 mL 20%

4-methylpiperidine v/v in DMF, 10 min, RT, 8 rpm, 2×), and the resin was washed (2 × DMF, 2 × DCM, 2 × DMF, 2 mL/wash). Fmoc-(3-aminomethyl) benzoic acid (100.8 mg, 270 µmol, 4.7 eq, 135 mM) was activated with DIC (41.4 µL, 265 µmol, 4.6 eq, 130 mM), HOAt (36.9 mg, 271 µmol, 4.7 eq, 140 mM), and DIEA (92 µL, 524 µmol, 9 eq, 260 mM) dissolved in DMF (2 mL). Activated amino acid was added to resin and incubated (3 h, RT, 8 rpm). The resin was then washed (2 × DMF, 2 × DCM, 2 × DMF, 2 mL/wash). Fmoc was removed (3 mL 20% 4-methylpiperidine v/v in DMF, 10 min, RT, 8 rpm, 2×), and the resin was washed (2 × DMF, 2 × DCM, 2 × DMF, 2 mL/ wash). 5-Chlorothiophene-2-carboxylic acid (43.3 mg, 270 µmol, 4.7 eq, 135 mM) was activated with DIC (41.4 µL, 265 µmol, 4.6 eq, 130 mM), HOAt (36.9 mg, 270 µmol, 4.7 eq, 135 mM), and DIEA (92 µL, 524 µmol, 9 eq, 260 mM) dissolved in DMF (2 mL). Activated carboxylic acid was added to resin and incubated (3 h, RT, 8 rpm). The resin was then washed (2 × DMF, 2 × DCM, 2 × DMF, 3 × DCM; 2 mL/ wash) and dried *in vacuo*. Dried resin was suspended in cleavage solution (95/2.5/2.5 TFA/TIS/H2O, 4.2 mL) and incubated (4 h, RT, 8 rpm). Cleavage solution was expelled and collected. Resin was washed with 10 mL DCM and collected. Collected solution was evaporated *in vacuo*. Crude product was dissolved (3 mL, 1:1:1 DMSO:MeOH:0.1% TFA/water) and purified by semi-preparative reverse phase HPLC. Fractions containing the intended product (as determined by MS analysis) were pooled, frozen, and evaporated by lyophilization, yielding a white solid (7.6 mg, 25.8 µmol, 45% yield).

^{1}H NMR (DMSO-d6, 400 MHz): δH 9.12 (m, 1H), 7.97 (s, 1H), 7.76 (d, 1H, J = 7.4 Hz), 7.69 (d, 1H, J = 4.1 Hz), 7.421 (t, 1H, J = 13.9 Hz), 7.42 (d, 1H, J = 12.9 Hz), 7.35 (s, 1H), 7.20 (d, 1H, J = 4.0 Hz), 4.48 (d, 2H, J = 5.9 Hz).

^{13}C NMR (DMSO-d6, 100 MHz): δC 167.8, 160.2, 139.3, 138.9, 134.4, 133.0, 130.1, 128.2, 128.13, 128.11, 126.6, 125.9, 42.4.

Synthesis of 12a: 2-benzyl-N-(3-carbamoylbenzyl)thiazole-4-carboxamide

Rink amide MBHA resin (151.4 mg, 61 µmol sites, Anaspec) was added to a fritted syringe (6 mL) and swelled in DMF (3 mL, 3 h, RT, 8 rpm). Solution was expelled and Fmoc was deprotected (20% 4-methylpiperidine, 2 × 3 mL, 10 min, RT, 8 rpm). The solution was expelled, and the resin was washed (2 × DMF, 2 × DCM, 2 × DMF, 2 mL/ wash). 3-(Fmoc-aminomethyl)benzoic acid (68.9 mg, 184 µmol, 3 eq, 88 mM) was activated with DIC (30 µL, 195 µmol, 3.3 eq, 93 mM), HOAt (34.9 mg, 256 µmol, 4.2 eq, 121 mM), and DIEA (65 µL, 373 µmol, 6.1 eq, 177 mM) dissolved in DMF (2 mL). Activated amino acid was added to resin and incubated (2 h, RT, 8 rpm). The resin was then washed (2 × DMF, 2 × DCM, 2 × DMF, 2 mL/ wash). Solution was expelled and Fmoc was deprotected (20% 4-methylpiperidine, 2 × 3 mL, 10 min, RT, 8 rpm). The resin was washed (2 × DMF, 2 × DCM, 2 × DMF, 2 mL/ wash). 2-benzyl-1,3-thiazole-4-carboxylic acid (39.6 mg, 186 µmol, 3 eq, 85 mM) was activated with DIC (30 µL, 195 µmol, 3.3 eq, 93 mM), HOAt (34.6 mg, 254 µmol, 4.2 eq, 121 mM), and DIEA (65 µL, 373 µmol, 6.1 eq, 177 mM) dissolved in DMF (2 mL). Activated carboxylic acid was added to resin and incubated (3 h, RT, 8 rpm). The resin was then washed (2 × DMF, 2 × DCM, 2 × DMF, 3 × DCM; 2 mL/ wash) and dried *in vacuo*. Dried resin was suspended in cleavage solution (95/2.5/2.5 TFA/TIS/H_2O, 2 mL) and incubated (4 h, RT, 8 rpm). Cleavage solution was expelled and collected. Resin was washed with DCM (10 mL) and collected. Collected solution was evaporated *in vacuo*. Crude product was dissolved (2 mL, 1:1 MeOH:0.1% TFA in H_2O and purified by semi-preparative reversed-phase HPLC. Fractions containing the intended product (as determined by MS analysis) were pooled, frozen, and evaporated by lyophilization, yielding a yellow solid (8.8 mg, 25.1 µmol, 40% yield).

^{1}H NMR (DMSO-d6, 400 MHz): δH 9.00 (t, 1H, J = 6.28 Hz), 8.14 (s, 1H), 7.97 (br, 1H), 7.84 (m, 1H), 7.74 (m, 1H), 7.46 (m, 1H), 7.37 (m, 6H), 7.28 (m, 1H), 7.37 (m, 6H), 7.28 (m, 1H),

4.49 (d, 2H, J = 6.32 Hz), 4.38 (s, 2H).

^{13}C NMR (DMSO-d6, 100 MHz): δC 170. 7, 167.9, 160.6, 149.6, 139.8, 137.8, 134.3, 130.2, 129.1, 128.8, 127.1, 126.8, 125.7, 124.2, 42.2, 38.5.

Synthesis of 12b: 3-(((1R,2R)-2-phenylcyclopropane-1-carboxamido)methyl)benzamide

Rink amide MBHA resin (151.2 mg 60 µmol sites, Anaspec) was added to a fritted syringe (6 mL) and swelled in DMF (3 mL, 3 h, RT, 8 rpm). Solution was expelled and Fmoc was deprotected (20% 4-methylpiperidine, 2 × 3 mL, 10 min, RT, 8 rpm). The solution was expelled, and the resin was washed (2 × DMF, 2 × DCM, 2 × DMF, 2 mL/ wash). 3-(Fmoc-aminomethyl)benzoic acid (68.6 mg, 184 µmol, 3 eq, 88 mM) was activated with DIC (30 µL, 195 µmol, 3.3 eq, 93 mM), HOAt (35.0 mg, 256 µmol, 4.2 eq, 121 mM), and DIEA (65 µL, 373 µmol, 6.1 eq, 177 mM) dissolved in DMF (2 mL). Activated amino acid was added to resin and incubated (2 h, RT, 8 rpm). The resin was then washed (2 × DMF, 2 × DCM, 2 × DMF, 2 mL/ wash). Solution was expelled and Fmoc was deprotected (20% 4-methylpiperidine, 2 × 3 mL, 10 min, RT, 8 rpm). The resin was washed (2 × DMF, 2 × DCM, 2 × DMF, 2 mL/ wash). *trans*-2-Phenyl-1-cyclopropanecarboxylic Acid (31.1 mg, 192 µmol, 3.2 eq, 91 mM) was activated with DIC (30 µL, 195 µmol, 3.3 eq, 93 mM), HOAt (32.9 mg, 242 µmol, 4 eq, 115 mM), and DIEA (65 µL, 373 µmol, 6.1 eq, 177 mM) dissolved in DMF (2 mL). Activated amino acid was added to resin and incubated (3 h, RT, 8 rpm). The resin was then washed (2 × DMF, 2 × DCM, 2 × DMF, 3× DCM; 2 mL/ wash) and dried *in vacuo*. Dried resin was suspended in cleavage solution (95/2.5/2.5 TFA/TIS/H_2O, 2 mL) and incubated (4 h, RT, 8 rpm). Cleavage solution was expelled and collected. Resin was washed with DCM (10 mL) and collected. Collected solution was evaporated *in vacuo*. Crude product was dissolved (2 mL, 1:1 MeOH:0.1% TFA in H_2O and purified by semi-preparative reversed-phase HPLC. Fractions containing the intended product (as confirmed by MS analysis) were pooled, frozen, and evaporated by lyophilization, yielding a white powder (6.5 mg, 22 µmol, 37% yield).

^{1}H NMR (DMSO-d6, 400 MHz): δH 8.67 (t, 1H, J = 5.7 Hz), 7.97 (s, 1H), 7.78 (s, 1H), 7.75 (t, 1H, J = 4.3 Hz), 7.40 (2, 2H, J = 4.6 Hz), 7.36 (s, 1H), 7.27 (m, 2H), 7.18 (d, 1H, J = 7.0 Hz), 7.13 (d, 2H, J = 7.8 Hz), 4.34 (m, 2H), 2.28 (m, 1H), 1.93 (m, 1H), 1.39 (m, 1H), 1.24 (m, 1H).

^{13}C NMR (DMSO-d6, 100 MHz): δC 171.0, 167.8, 141.1, 139.6, 134.4, 130.2, 130.1, 128.4, 126.7, 126.6, 126.0, 125. 8, 42.3, 25.8, 24.0, 15.3.

Synthesis of 13a: N-(2-(2-amino-2-oxoethoxy)-10,11-dihydro-5H-dibenzo-[a,d][7]annulen-5-yl)-8-methylimidazo[1,2-a]pyridine-2-carboxamide

Sieber amide resin (140.4 mg, 80 umol sites, Anaspec) was added to a fritted syringe (6 mL) and swelled in DMF (3 mL, 2 h, RT, 8 rpm). Solution was expelled and Fmoc was deprotected (20% 4-methylpiperidine, 2 × 3 mL, 10 min, RT, 8 rpm). The solution was expelled, and the resin was washed (2 × DMF, 2 × DCM, 2 × DMF, 2 mL/ wash). Fmoc suberol (123.4 mg, 240 µmol, 3 eq, 110 mM) was activated with DIC (37.2 µL, 240 µmol, 3 eq, 110 mM), HOAt (32.5 mg, 240 µmol, 3 eq, 110 mM), and DIEA (83.7 µL, 480 µmol, 6 eq, 220 mM) dissolved in DMF (2 mL). Activated amino acid was added to resin and incubated (2 h, RT, 8 rpm). The resin was then washed (2 × DMF, 2 × DCM, 2 × DMF, 2 mL/ wash). Solution was expelled and Fmoc was deprotected (20% 4-methylpiperidine, 2 × 3 mL, 10 min, RT, 8 rpm). The resin was washed (2 × DMF, 2 × DCM, 2 × DMF, 2 mL/ wash). 8-Methyl-imidazo[1,2-a]pyridine-2-carboxylic acid (42.3 mg, 240 µmol, 3 eq, 110 mM) was activated with DIC (37.2 µL, 240 µmol, 3 eq, 110 mM), HOAt (34.1 mg, 240 µmol, 3 eq, 110 mM), and DIEA (83.7 µL, 480 µmol, 6 eq, 220 mM) dissolved in DMF (2 mL). Activated carboxylic acid was added to resin and incubated (3 h, RT, 8 rpm). The resin was then washed (2 × DMF, 2 × DCM, 2 × DMF, 3× DCM; 2 mL/ wash) and dried *in vacuo*. Dried resin was suspended in cleavage solution (1% TFA in DCM, 3.5 mL) and incubated (1 h, RT, 8 rpm). Cleavage solution was expelled and collected. Resin was washed with DCM (10 mL) and collected. Collected solution was evaporated *in vacuo*, leaving a clear oil. Crude product was dissolved in DCM (10 mL), loaded to celite, dried *in vacuo*, and purified on silica (RediSep Rf Gold Silica Gel column, spherical, 20-40 µm, 60 Å, Teledyne ISCO, Thousand Oaks, CA) by automated flash chromatography(CombiflashRf+, Teledyne ISCO, Thousand Oaks, CA) with gradient elution (0-20% B, A = DCM; B = 90:10 MeOH:14.8M NH_3OH in H_2O) yielding a white solid (9.2 mg, 21 µmol, 26% yield).

^{1}H NMR (DMSO-d6, 400 MHz): δH 8.60 (d, 1H, J = 8.5 Hz), 8.36 (m, 2H), 7.46 (s, 1H), 7.37 (m, 3H), 7.12 (m, 4H), 6.84 (m, 1H), 6.76 (m, 1H), 6.71 (dd, 1H, J=3.7), 6.38 (d, 1H, J = 8.4 Hz), 4.34 (s, 2H), 3.27 (m, 2H), 3.07 (m, 2H), 1.19 (s, 3H).

^{13}C NMR(DMSO-d6, 100 MHz): δC 171.0, 165.3, 161.9, 158.2, 145.4, 141.2, 140.2, 139.8, 139.4, 132.6, 131,2, 128.6, 128.0, 127.1, 126.4, 125.9, 125.5, 117.4, 116.3, 114.3, 113.0, 67.8, 56.2, 33.4, 32.9, 17.5.

Synthesis of 13b: N-(2-(2-amino-2-oxoethoxy)-10,11-dihydro-5H-dibenzo-[a,d][7]annulen-5-yl)-5-chlorofuran-2-carboxamide

Sieber amide resin (147.6 mg, 84 µmol sites) was added to a fritted syringe (6 mL) and swelled in DMF (3 mL, 2 h, RT, 8 rpm). Solution was expelled and Fmoc was deprotected (20% 4-methylpiperidine, 2 × 3 mL, 10 min, RT, 8 rpm). The solution was expelled, and the resin was washed (2 × DMF, 2 × DCM, 2 × DMF, 2 mL/ wash). Fmoc suberol (212.0 mg, 420 µmol, 5 eq, 140 mM) was activated with DIC (70 µL, 455 µmol, 5.4 eq, 150 mM), HOAt (62.4 mg, 460 µmol, 5.4 eq, 150 mM), and DIEA (155 µL, 890 µmol, 10.6 eq, 300 mM) dissolved in DMF (3 mL). Activated amino acid was added to resin and incubated (2 h, RT, 8 rpm). The resin was then washed (2 × DMF, 2 × DCM, 2 × DMF, 2 mL/ wash). Solution was expelled and Fmoc was deprotected (20% 4-methylpiperidine, 2 × 3 mL, 10 min, RT, 8 rpm). The resin was washed (2 × DMF, 2 × DCM, 2 × DMF, 2 mL/ wash). 5-chloro-2-furoic acid (61.4 mg, 420 µmol, 5 eq, 140 mM) was activated with DIC (70 µL, 455 µmol, 5.4 eq, 150 mM), HOAt (62.2 mg, 460 µmol, 5.4 eq, 150 mM), and DIEA (155 µL, 890 µmol, 10.6 eq, 300 mM) dissolved in DMF (2 mL). Activated carboxylic acid was added to resin and incubated (3 h, RT, 8 rpm). The resin was then washed (2 × DMF, 2 × DCM, 2 × DMF, 3× DCM; 2 mL/ wash) and dried *in vacuo*. Dried resin was suspended in cleavage solution (1% TFA in DCM, 5 mL) and incubated (1 h, RT, 8 rpm). Cleavage solution was expelled and collected. Resin was washed with DCM (10 mL) and collected. Collected solution was evaporated *in vacuo*, leaving a brown oil. Crude product was dissolved in DCM (10 mL), loaded to celite, dried *in vacuo*, and purified on silica (RediSep Rf Gold Silica Gel column, spherical, 20-40 µm, 60 Å) by automated flash chromatography(CombiflashRf+) with gradient elution (0-20% B, A = DCM; B = MeOH), yielding a white solid (8.6 mg, 21 µmol, 25% yield).

^{1}H NMR (DMSO-d6, 400 MHz): δH 9.27 (d, 1H, J = 7.7 Hz), 7.44 (m, 1H), 7.40 (m, 2H), 7.34 (m, 2H), 7.17 (m, 3H), 6.76 (m, 2H), 6.64 (d, 1H, J = 3.6 Hz), 6.36 (d, 1H, J = 7.7 Hz), 4.38 (s,

2H), 3.30 (m, 2H), 3.06 (m, 2H).

^{13}C NMR(DMSO-d6, 100 MHz): δC 169.9, 157.0, 155.7, 147.0, 140.4, 139.0, 138.9, 137.4, 131.3, 129.8, 129.6, 128.1, 127.4 125.7, 116.08, 116.05, 111.8, 109.0, 66.7, 55.0. 32.2, 31.7.

Synthesis of 16a: 2-(3-(1-(4-chloro-2-(trifluoromethyl)benzoyl)piperidin-4-yl)-2-oxo-2,3-dihydro-1H-benzo[d]imidazol-1-yl)acetamide

Rink amide MBHA resin (151.2 mg 60 µmol sites, Anaspec) was added to a fritted syringe (6 mL) and swelled in DMF (3 mL, 3 h, RT, 8 rpm). Solution was expelled and Fmoc was deprotected (20% 4-methylpiperidine, 2 × 3 mL, 10 min, RT, 8 rpm). The solution was expelled, and the resin was washed (2 × DMF, 2 × DCM, 2 × DMF, 2 mL/ wash). Fmoc-4-(3-carboxymethyl-2-keto-1-benzimidazolyl)-piperidine (90.5 mg, 182 µmol, 3 eq, 86 mM) was activated with DIC (30 µL, 195 µmol, 3.3 eq, 95 mM), HOAt (26.3 mg, 193 µmol, 3.2 eq, 97 mM), and DIEA (70 µL, 400 µmol, 6.7 eq, 190 mM) dissolved in DMF (2 mL). Activated amino acid was added to resin and incubated (2 h, RT, 8 rpm). The resin was then washed (2 × DMF, 2 × DCM, 2 × DMF, 2 mL/ wash). Solution was expelled and Fmoc was deprotected (20% 4-methylpiperidine, 2 × 3 mL, 10 min, RT, 8 rpm). The resin was washed (2 × DMF, 2 × DCM, 2 × DMF, 2 mL/ wash). 4-Chloro-2-(trifluoromethyl)benzoic acid (40.3 mg, 180 µmol, 3 eq, 86 mM) was activated with DIC (30 µL, 195 µmol, 3.3 eq, 97 mM), HOAt (27.1 mg, 200 µmol, 3.3 eq, 95 mM), and DIEA (70 µL, 400 µmol, 6.7 eq, 190 mM) dissolved in DMF (2 mL). Activated carboxylic acid was added to resin and incubated (3 h, RT, 8 rpm). The resin was then washed (2 × DMF, 2 × DCM, 2 × DMF, 3× DCM; 2 mL/ wash) and dried *in vacuo*. Dried resin was suspended in cleavage solution (95/2.5/2.5 TFA/TIS/H_2O, 2 mL) and incubated (4 h, RT, 8 rpm). Cleavage solution was expelled and collected. Resin was washed with DCM (10 mL) and collected. Collected solution was evaporated *in vacuo*, leaving a clear oil. Crude product was dissolved (2 mL, 1:1 MeOH:0.1% TFA in H_2O and purified by semi-preparative reversed-phase HPLC. Fractions containing product (as confirmed by MS analysis) were pooled, frozen, and evaporated by lyophilization, yielding a white powder (11.5 mg, 24 µmol, 40% yield).

^{1}H NMR (DMSO-d6, 400 MHz): δH 7.95 (m, 1H), 7.88 (m, 1H), 7.65 (s, 1H), 7.52 (m, 1H), 7.25 (m, 1H), 7.14 (m, 1H), 7.04 (m, 3H), 4.68 (m, 1H). 4.54 (m, 1H), 4.40 (m, 2H), 3.27 (m, 2H), 2.95 (m, 1H), 2.28 (m, 2H), 1.84 (m, 1H), 1.64 (m, 1H).

^{13}C NMR(DMSO-d6, 100 MHz): δC 168.7, 165.1, 153.0, 134.1, 133.2, 129.6, 127.9, 127.0, 124.3, 120.8, 108.2, 50.2, 46.2, 42.9, 28.4.

Synthesis of 16b: 2-(3-(1-(1-(cyclohexylmethyl)-1H-pyrazole-4-carbonyl)-piperidin-4-yl)-2-oxo-2,3-dihydro-1H-benzo[d]imidazol-1-yl)acetamide

Rink amide MBHA resin (148.3 mg 59 µmol sites, Anaspec) was added to a fritted syringe (6 mL) and swelled in DMF (3 mL, 3 h, RT, 8 rpm). Solution was expelled and Fmoc was deprotected (20% 4-methylpiperidine, 2 × 3 mL, 10 min, RT, 8 rpm). The solution was expelled, and the resin was washed (2 × DMF, 2 × DCM, 2 × DMF, 2 mL/ wash). Fmoc-4-(3-carboxymethyl-2-keto-1-benzimidazolyl)-piperidine (89.7 mg, 180 µmol, 3 eq, 130 mM) was activated with DIC (30 µL, 195 µmol, 3.3 eq, 140 mM), HOAt (25.2 mg, 185 µmol, 3.1 eq, 132 mM), and DIEA (65 µL, 373 µmol, 6.3 eq, 266 mM) dissolved in DMF (1.4 mL). Activated amino acid was added to resin and incubated (2 h, RT, 8 rpm). The resin was then washed (2 × DMF, 2 × DCM, 2 × DMF, 2 mL/ wash). Solution was expelled and Fmoc was deprotected (20% 4-methylpiperidine, 2 × 3 mL, 10 min, RT, 8 rpm). The resin was washed (2 × DMF, 2 × DCM, 2 × DMF, 2 mL/ wash). 1-(cyclohexylmethyl)-1H-pyrazole-4-carboxylic acid (37.2 mg, 178 µmol, 3 eq, 127 mM) was activated with DIC (30 µL, 195 µmol, 3.3 eq, 140 mM), HOAt (25.2 mg, 185 µmol, 3.1 eq, 132 mM), and DIEA (65 µL, 373 µmol, 6.3 eq, 266 mM) dissolved in DMF (1.4 mL). Activated carboxylic acid was added to resin and incubated (3 h, RT, 8 rpm). The resin was then washed (2 × DMF, 2 × DCM, 2 × DMF, 3× DCM; 2 mL/ wash) and dried *in vacuo*. Dried resin was suspended in cleavage solution (95/2.5/2.5 TFA/TIS/H_2O, 2 mL) and incubated (4 h, RT, 8 rpm).

Cleavage solution was expelled and collected. Resin was washed with DCM (10 mL) and collected. Collected solution was evaporated *in vacuo*, leaving a clear oil. Crude product was dissolved (2 mL, 1:1 MeOH:0.1% TFA in H_2O and purified by semi-preparative reversed-phase HPLC. Fractions containing product (as confirmed by MS analysis) were pooled, frozen, and evaporated by lyophilization, yielding a white powder (8.5 mg, 18 µmol, 30% yield).

^{1}H NMR (DMSO-d6, 400 MHz): δH 8.10 (s, 1H), 7.71 (s, 1H), 7.64 (s, 1H), 7.32 (m, 1H), 7.22 (s, 1H), 7.03 (m, 3H), 4.51 (m, 2H), 4.41 (m, 3H), 3.97 (d, 2H, J =7.2 Hz), 3.08 (br, 2H), 2.29 (m, 2H), 1.80 (m, 3H), 1.64 (m, 3H), 1.49 (d, 2H), 1.19 (m, 3H), 0.94 (m, 2H).

^{13}C NMR(DMSO-d6, 100 MHz): δC 168.6, 162.7, 153.0, 139.2, 129.6, 128.1, 120.8, 120.6, 116.0, 108.7, 108.2, 57.3, 50.6, 42.8, 38.0, 29.8, 29.0, 26.7, 25.9, 25.1.

Synthesis of 17a: 2-(1-(cyclohexylmethyl)-1H-pyrazole-4-carbonyl)-1,2,3,4-tetrahydroisoquinoline-6-carboxamide

Rink amide MBHA resin (151.0 mg 60 µmol sites, Anaspec) was added to a fritted syringe (6 mL) and swelled in DMF (3 mL, 3 h, RT, 8 rpm). Solution was expelled and Fmoc was deprotected (20% 4-methylpiperidine, 2 × 3 mL, 10 min, RT, 8 rpm). The solution was expelled, and the resin was washed (2 × DMF, 2 × DCM, 2 × DMF, 2 mL/ wash). 2-[(9H-fluoren-9-yl)methoxy]carbonyl-1,2,3,4-tetrahydroisoquinoline-6-carboxylic acid (71.9 mg, 180 µmol, 3 eq, 85 mM) was activated with DIC (30 µL, 195 µmol, 3.3 eq, 93 mM), HOAt (26.9 mg, 197 µmol, 3.3 eq, 94 mM), and DIEA (70 µL, 400 µmol, 6.7 eq, 190 mM) dissolved in DMF (2 mL). Activated amino acid was added to resin and incubated (2 h, RT, 8 rpm). The resin was then washed (2 × DMF, 2 × DCM, 2 × DMF, 2 mL/ wash). Solution was expelled and Fmoc was deprotected (20% 4-methylpiperidine, 2 × 3 mL, 10

min, RT, 8 rpm). The resin was washed (2 × DMF, 2 × DCM, 2 × DMF, 2 mL/ wash). 1-(cyclohexylmethyl)-1H-pyrazole-4-carboxylic acid (37.3 mg, 179 µmol, 3 eq, 85 mM) was activated with DIC (30 µL, 195 µmol, 3.3 eq, 93 mM), HOAt (26.3 mg, 193 µmol, 3.1 eq, 132 mM), and DIEA (70 µL, 400 µmol, 6.7 eq, 190 mM) dissolved in DMF (2 mL). Activated carboxylic acid was added to resin and incubated (3 h, RT, 8 rpm). The resin was then washed (2 × DMF, 2 × DCM, 2 × DMF, 3× DCM; 2 mL/ wash) and dried *in vacuo*. Dried resin was suspended in cleavage solution (95/2.5/2.5 TFA/TIS/H_2O, 2 mL) and incubated (4 h, RT, 8 rpm). Cleavage solution was expelled and collected. Resin was washed with DCM (10 mL) and collected. Collected solution was evaporated *in vacuo*, leaving a clear oil. Crude product was dissolved (2 mL, 1:1 MeOH:0.1% TFA in H_2O and purified by semi-preparative reversed-phase HPLC (15% – 75% B, 16 min). Fractions containing product (as confirmed by MS analysis) were pooled, frozen, and evaporated by lyophilization, yielding a white powder (8.2 mg). Product was dissolved in DMSO (0.5 mL) and 0.1% TFA water (1.5 mL) and purified again by semi-preparative reversed-phase HPLC (10% – 55% B, 16 min). Fractions containing product (as determined by MS analysis) were pooled, frozen, and evaporated by lyophilization, yielding a white powder (3.8 mg, 10.4 µmol, 17% yield).

^{1}H NMR (DMSO-d6, 400 MHz): δH 8.15 (s, 1H), 7.92 (s, 1H), 7.77 (s, 1H), 7.71 (m, 2H), 7.30 (m, 2H), 4.78 (s, 2H), 3.98 (d, 2H, J = 7.3 Hz), 3.82 (t, 2H, J = 5.5 Hz), 2.92 (m, 2H), 1.83 (m, 1H), 1.64 (m, 3H), 1.49 (m, 2H), 1.15 (m, 3H), 0.94 (m, 2H).

Supporting Tables

Table S1. Library Amino acid SMILES

Amino Acid	SMILES
1	C1CN(CCC1C(=O)O)C(=O)OCC2C3=CC=CC=C3C4=CC=CC=C24
2	C1CN(CCN1CC(=O)O)C(=O)OCC2C3=CC=CC=C3C4=CC=CC=C24
3	C1C[C@H](N(C1)C(=O)OCC2C3=CC=CC=C3C4=CC=CC=C24)C(=O)O
4	C1C[C@@H](N(C1)C(=O)OCC2C3=CC=CC=C3C4=CC=CC=C24)C(=O)O
5	CC(C)[C@@H](C(=O)O)N(C)C(=O)OCC1C2=CC=CC=C2C3=CC=CC=C13
6	CC(C)[C@H](C(=O)O)N(C)C(=O)OCC1C2=CC=CC=C2C3=CC=CC=C13
7	CC(C)C[C@@H](C(=O)O)N(C)C(=O)OCC1C2=CC=CC=C2C3=CC=CC=C13
8	CC(C)C[C@H](C(=O)O)N(C)C(=O)OCC1C2=CC=CC=C2C3=CC=CC=C13
9	C[C@@H](C(=O)O)N(C)C(=O)OCC1C2=CC=CC=C2C3=CC=CC=C13
10	C[C@H](C(=O)O)N(C)C(=O)OCC1C2=CC=CC=C2C3=CC=CC=C13
11	C1CN([C@@H]1C(=O)O)C(=O)OCC2C3=CC=CC=C3C4=CC=CC=C24
12	C1C(CN1C(=O)OCC2C3=CC=CC=C3C4=CC=CC=C24)C(=O)O
13	C1C[C@H](CN(C1)C(=O)OCC2C3=CC=CC=C3C4=CC=CC=C24)C(=O)O
14	C1C[C@@H](CN(C1)C(=O)OCC2C3=CC=CC=C3C4=CC=CC=C24)C(=O)O
15	C1CCN([C@H](C1)C(=O)O)C(=O)OCC2C3=CC=CC=C3C4=CC=CC=C24
16	C1C(N(C2=CC=CC=C21)C(=O)OCC3C4=CC=CC=C4C5=CC=CC=C35)C(=O)O
17	C1CN(CCC1(C2=CC=CC=C2)C(=O)O)C(=O)OCC3C4=CC=CC=C4C5=CC=CC=C35
18	CC(C)C[C@@H]([C@H](CC(=O)O)O)NC(=O)OCC1C2=CC=CC=C2C3=CC=CC=C13
19	OC(C[C@H](C)[C@H](CC1=CC=CC=C1)NC(OCC2C(C=CC=C3)=C3C4=C2C=CC=C4)=O)=O
20	C1[C@H](N(CC2=CC=CC=C21)C(=O)OCC3C4=CC=CC=C4C5=CC=CC=C35)C(=O)O
21	C1[C@@H](N(CC2=CC=CC=C21)C(=O)OCC3C4=CC=CC=C4C5=CC=CC=C35)C(=O)O
22	C1CCC(CC1)C[C@H](C(=O)O)NC(=O)OCC2C3=CC=CC=C3C4=CC=CC=C24
23	C[C@H](NC(OCC1C2=CC=CC=C2C3=CC=CC=C13)=O)[C@@H](O)CC(O)=O
24	C1CN(CC(N1)C(=O)O)C(=O)OCC2C3=CC=CC=C3C4=CC=CC=C24
25	C1=CC=C2C(=C1)C(C3=CC=CC=C32)COC(=O)N[C@H](CC(=O)O)C4=CC=C(C=C4)C(F)(F)F
26	C1CN(CCC12C(=O)N(CN2C3=CC=CC=C3)CC(=O)O)C(=O)OCC4C5=CC=CC=C5C6=CC=CC=C46
27	CC1(OC2=C(O1)C=C(C=C2)C[C@@H](C(=O)O)NC(=O)OCC3C4=CC=CC=C4C5=CC=CC=C35)C
28	O=C(N(CC1=C(O)C=C(OC)C=C1)CC(O)=O)OCC2C(C=CC=C3)=C3C4=C2C=CC=C4
29	C1=CC=C(C=C1)CN2C=C(N=C2)C[C@@H](C(=O)O)NC(=O)OCC3C4=CC=CC=C4C5=CC=CC=C35
30	CC1=CC=C(C=C1)S(=O)(=O)N2C=C(N=C2)C[C@@H](C(=O)O)NC(=O)OCC3C4=CC=CC=C4C5=CC=CC=C35
31	C1CC(C1)(C(=O)O)NC(=O)OCC2C3=CC=CC=C3C4=CC=CC=C24
32	C1CC[C@H]([C@H](C1)C(=O)O)NC(=O)OCC2C3=CC=CC=C3C4=CC=CC=C24
33	C1=CC=C(C=C1)COP(=O)(O)OC[C@@H](C(=O)O)NC(=O)OCC2C3=CC=CC=C3C4=CC=CC=C24
34	C1CSCCC1(C(=O)O)NC(=O)OCC2C3=CC=CC=C3C4=CC=CC=C24
35	C1=CC=C(C=C1)S(=O)(=O)N[C@@H](CNC(=O)OCC2C3=CC=CC=C3C4=CC=CC=C24)C(=O)O
36	C1C[C@H](N(C1)C(=O)OCC2C3=CC=CC=C3C4=CC=CC=C24)CC(=O)O
37	C1C(CC2=CC=CC=C21)[C@H](C(=O)O)NC(=O)OCC3C4=CC=CC=C4C5=CC=CC=C35
38	C1C2=CC=CC=C2C(N1C(=O)OCC3C4=CC=CC=C4C5=CC=CC=C35)C(=O)O
39	C1[C@@H](N(CC2=C1C=CC(=C2)O)C(=O)OCC3C4=CC=CC=C4C5=CC=CC=C35)C(=O)O
40	C1C[C@@](CN(C1)C(=O)OCC2C3=CC=CC=C3C4=CC=CC=C24)(CC5=CC=CC=C5)C(=O)O
41	CC(C)([C@H](C(=O)O)NC(=O)OCC1C2=CC=CC=C2C3=CC=CC=C13)O
42	C1[C@@H](CN([C@@H]1C(=O)O)C(=O)C2=CC=CC=C2)NC(=O)OCC3C4=CC=CC=C4C5=CC=CC=C35
43	C1=CC=C(C=C1)[C@@H](C(=O)O)NC(=O)OCC2C3=CC=CC=C3C4=CC=CC=C24
44	C[C@H]1C(=O)N(CCN1C(=O)OCC2C3=CC=CC=C3C4=CC=CC=C24)CC(=O)O

Table S1. Continued from previous page.

Amino Acid	SMILES
45	C1CC2(CCC1(C(=O)O)NC(=O)OCC3C4=CC=CC=C4C5=CC=CC=C35)OCCO2
46	C1[C@H](N(CC1=O)C(=O)OCC2C3=CC=CC=C3C4=CC=CC=C24)C(=O)O
47	C1CCC(C1)[C@H](C(=O)O)NC(=O)OCC2C3=CC=CC=C3C4=CC=CC=C24
48	C1[C@@H](C=C[C@@H]1NC(=O)OCC2C3=CC=CC=C3C4=CC=CC=C24)C(=O)O
49	C1=CC=C(C=C1)C2=NC(C(=O)N(C3=CC=CC=C32)CC(=O)O)NC(=O)OCC4C5=CC=CC=C5C6=CC=CC=C46
50	C1CN(C(=O)CN1C(=O)OCC2C3=CC=CC=C3C4=CC=CC=C24)CC(=O)O
51	C1CSC[C@H](N1C(=O)OCC2C3=CC=CC=C3C4=CC=CC=C24)C(=O)O
52	C1CN(CCC1(C2=CC3=CC=CC=C3C=C2)C(=O)O)C(=O)OCC4C5=CC=CC=C5C6=CC=CC=C46
53	C1C[C@H](N([C@H]1C2=CC=CC=C2)C(=O)OCC3C4=CC=CC=C4C5=CC=CC=C35)C(=O)O
54	C1COCCC1(CC(=O)O)NC(=O)OCC2C3=CC=CC=C3C4=CC=CC=C24
55	CC(C)C[C@@H](C(=O)O)N1CC[C@@H](C1=O)NC(=O)OCC2C3=CC=CC=C3C4=CC=CC=C24
56	C[N+](C)(CCCC[C@H](NC(OCC1C2=CC=CC=C2C3=CC=CC=C13)=O)C(O)=O)C
57	O=C(N[C@@H]1C(N([C@H](C(O)=O)CS2)[C@]2([H])CC1)=O)OCC3C(C=CC=C4)=C4C5=C3C=CC=C5
58	CC(C)(C)[C@@H](C(=O)O)NC(=O)OCC1C2=CC=CC=C2C3=CC=CC=C13
59	CC1=CC=C(C=C1)S(=O)(=O)NC(=NCCC[C@@H](C(=O)O)NC(=O)OCC2C3=CC=CC=C3C4=CC=CC=C24)N
60	CS(=O)CC[C@@H](C(=O)O)NC(=O)OCC1C2=CC=CC=C2C3=CC=CC=C13
61	C1[C@H](N(CS1)C(=O)OCC2C3=CC=CC=C3C4=CC=CC=C24)C(=O)O
62	C1=CC=C(C=C1)COC2=CC=C(C=C2)C[C@@H](C(=O)O)NC(=O)OCC3C4=CC=CC=C4C5=CC=CC=C35
63	C1CCN([C@@H](C1)C(=O)O)C(=O)OCC2C3=CC=CC=C3C4=CC=CC=C24
64	C1CCC(C1)(C(=O)O)NC(=O)OCC2C3=CC=CC=C3C4=CC=CC=C24
65	C1[C@H](N(CC2=C1C3=CC=CC=C3N2)C(=O)OCC4C5=CC=CC=C5C6=CC=CC=C46)C(=O)O
66	C1=CC=C2C=C(C=CC2=C1)C[C@@H](C(=O)O)NC(=O)OCC3C4=CC=CC=C4C5=CC=CC=C35
67	C1CC1C[C@@H](C(=O)O)NC(=O)OCC2C3=CC=CC=C3C4=CC=CC=C24
68	C1=CC=C2C(=C1)C(C3=CC=CC=C32)COC(=O)NCC4=CC(=CC=C4)C(=O)O
69	C1=CC=C2C(=C1)C(C3=CC=CC=C32)COC(=O)N[C@@H](CC4=CC=CS4)C(=O)O
70	C1=CC=C(C=C1)C2=CC=C(C=C2)C[C@@H](C(=O)O)NC(=O)OCC3C4=CC=CC=C4C5=CC=CC=C35
71	CC(=O)NCSC(C)(C)[C@@H](C(=O)O)NC(=O)OCC1C2=CC=CC=C2C3=CC=CC=C13
72	C1CC(CCC1CC(=O)O)NC(=O)OCC2C3=CC=CC=C3C4=CC=CC=C24
73	C1CCN(C(=O)[C@H](C1)NC(=O)OCC2C3=CC=CC=C3C4=CC=CC=C24)CC(=O)O
74	C1CN(C(C(=O)N1)CC(=O)O)C(=O)OCC2C3=CC=CC=C3C4=CC=CC=C24
75	C1CC(CCC1C2=CC=CC=C2)(C(=O)O)NC(=O)OCC3C4=CC=CC=C4C5=CC=CC=C35
76	C1CC2=CC=CC=C2N(C(=O)C1NC(=O)OCC3C4=CC=CC=C4C5=CC=CC=C35)CC(=O)O
77	C1=CC=C(C(=C1)CNC(=O)OCC2C3=CC=CC=C3C4=CC=CC=C24)C5=CC=CC=C5C(=O)O
78	C1CC(CCC1=O)(C(=O)O)NC(=O)OCC2C3=CC=CC=C3C4=CC=CC=C24
79	C1[C@H](CN([C@@H]1C(=O)O)C(=O)OCC2C3=CC=CC=C3C4=CC=CC=C24)F
80	CC(C)([C@H](C(=O)O)NC(=O)OCC1C2=CC=CC=C2C3=CC=CC=C13)SCC4=CC=CC=C4
81	C1=CC=C(C=C1)[C@H]([C@H](C(=O)O)NC(=O)OCC2C3=CC=CC=C3C4=CC=CC=C24)O
82	C1[C@@H]2[C@H]1C(N(C2)C(=O)OCC3C4=CC=CC=C4C5=CC=CC=C35)C(=O)O
83	C1[C@@H](C(=O)N(C2=CC=CC=C2S1)CC(=O)O)NC(=O)OCC3C4=CC=CC=C4C5=CC=CC=C35
84	C1=CC=C2C(=C1)C(C3=CC=CC=C32)COC(=O)N[C@@H](CC4=CC=C(C=C4)CP(=O)(O)O)C(=O)O
85	C1=CC=C2C(=C1)C(C3=CC=CC=C32)COC(=O)N[C@@H](CC4=CC(=C(C=C4)F)F)C(=O)O
86	C1=CC=C2C(=C1)C(C3=CC=CC=C32)COC(=O)N[C@@H](CC4=CSC=N4)C(=O)O
87	C[C@H]([C@@H](C(=O)O)NC(=O)OCC1C2=CC=CC=C2C3=CC=CC=C13)OP(=O)(O)OCC4=CC=CC=C4
88	C1=CC=C2C(=C1)C(C3=CC=CC=C32)COC(=O)N[C@H](CC4=CN=CC=C4)C(=O)O

Table S1. Continued from previous page.

Amino Acid	SMILES
89	C1COCC(N1C(=O)OCC2C3=CC=CC=C3C4=CC=CC=C24)C(=O)O
90	C1=CC=C(C=C1)COC(=O)C[C@@H](C(=O)O)NC(=O)OCC2C3=CC=CC=C3C4=CC=CC=C24
91	C[C@@H]1[C@H](N(C(O1)(C)C)C(=O)[C@H](C(C)C)NC(=O)OCC2C3=CC=CC=C3C4=CC=CC=C24)C(=O)O
92	C1=CC=C2C(=C1)C(C3=CC=CC=C32)COC(=O)N[C@@H](CC4=CC=C(C=C4)OP(=O)(O)O)C(=O)O
93	C1=CC=C2C(=C1)C(C3=CC=CC=C32)COC(=O)N[C@@H](CC(=O)O)C4=CC=CC=C4C(F)(F)F
94	C1=CC=C(C=C1)[C@H](C(=O)O)NC(=O)OCC2C3=CC=CC=C3C4=CC=CC=C24
95	C1=CC=C2C(=C1)C(C3=CC=CC=C32)COC(=O)N[C@@H](CC(=O)O)C4=C(C=C(C=C4)Cl)Cl
96	C1=CC=C2C(=C1)C(C3=CC=CC=C32)COC(=O)N[C@@H](CC(=O)O)C4=CC(=CC=C4)O
97	COC1=NC=C(C=C1)[C@@H](CC(=O)O)NC(=O)OCC2C3=CC=CC=C3C4=CC=CC=C24
98	C1=CC=C2C(=C1)C(C3=CC=CC=C32)COC(=O)N[C@H](CC(=O)O)C4=CC=CO4
99	C1=CC=C2C(=C1)C(C3=CC=CC=C32)COC(=O)N[C@@H](CC4=CC=C(C=C4)OCC5=C(C=CC=C5Cl)Cl)C(=O)O
100	C1=CC=C2C(=C1)C(C3=CC=CC=C32)COC(=O)NC(CC4=CC(=C(C=C4)O)F)C(=O)O
101	C1CC2=C(C=CC(=C2)OCC(=O)O)C(C3=CC=CC=C31)NC(=O)OCC4C5=CC=CC=C5C6=CC=CC=C46
102	C1CN(CCC1NC(=O)OCC2C3=CC=CC=C3C4=CC=CC=C24)CC(=O)O
103	C1CCC(CC1)C2(CCN(CC2)C(=O)OCC3C4=CC=CC=C4C5=CC=CC=C35)C(=O)O
104	C1=CC=C(C(=C1)CC(=O)O)CNC(=O)OCC2C3=CC=CC=C3C4=CC=CC=C24
105	COP(=O)(OC)OC1=CC=C(C=C1)C[C@@H](C(=O)O)NC(=O)OCC2C3=CC=CC=C3C4=CC=CC=C24
106	C1CCC(CC1)C2=NC(C(=O)N(C3=CC=CC=C32)CC(=O)O)NC(=O)OCC4C5=CC=CC=C5C6=CC=CC=C46
107	O=C(N1[C@@H](C(O)=O)C(C)(C)SC1)OCC2C(C=CC=C3)=C3C4=C2C=CC=C4
108	C1CN(CCC1N2C3=CC=CC=C3N(C2=O)CC(=O)O)C(=O)OCC4C5=CC=CC=C5C6=CC=CC=C46
109	O=C(OCC1C2=C(C3=C1C=CC=C3)C=CC=C2)NCCN4C(C(C=CC=C5)=C5N(CC(O)=O)C4=O)=O
110	O=C(OCC1C2=C(C3=C1C=CC=C3)C=CC=C2)N[C@H]4C[C@@H](C(N[C@@H](CC(O)=O)C5=O)=O)N5C4
111	C1C[C@@H](C(=O)N(C1)CC(=O)O)NC(=O)OCC2C3=CC=CC=C3C4=CC=CC=C24
112	C1CN(CCN1C2=CC3=C(C=C2)N=CN(C3=O)CC(=O)O)C(=O)OCC4C5=CC=CC=C5C6=CC=CC=C46
113	C1CC2=C3C(=CC=C2)C[C@H](N3C(=O)[C@H]1NC(=O)OCC4C5=CC=CC=C5C6=CC=CC=C46)C(=O)O
114	O=C(OCC1C2=C(C3=C1C=CC=C3)C=CC=C2)N[C@H](C(O)=O)CC4=CC(C=CN5)=C5C=C4
115	C1C[C@H]2C[C@@H]1[C@@H]([C@@H]2NC(=O)OCC3C4=CC=CC=C4C5=CC=CC=C35)C(=O)O
116	C1=CC=C2C(=C1)C(C3=CC=CC=C32)COC(=O)N[C@@H](CC4=NNN=N4)C(=O)O
117	C1CN([C@@H](C[C@@H]1O)C(=O)O)C(=O)OCC2C3=CC=CC=C3C4=CC=CC=C24
118	C1[C@@H](C(=O)N(CC2=CC=CC=C21)CC(=O)O)NC(=O)OCC3C4=CC=CC=C4C5=CC=CC=C35
119	C1[C@@H]([C@H](N1C(=O)OCC2C3=CC=CC=C3C4=CC=CC=C24)C(=O)O)C5=CC=CC=C5
120	C1[C@@H](N(CS1)C(=O)OCC2C3=CC=CC=C3C4=CC=CC=C24)C(=O)O
121	C1C2=CC=CC=C2CC1(C(=O)O)NC(=O)OCC3C4=CC=CC=C4C5=CC=CC=C35
122	C1CN([C@H](C[C@H]1O)C(=O)O)C(=O)OCC2C3=CC=CC=C3C4=CC=CC=C24
123	C1=CC=C2C(=C1)C(C3=CC=CC=C32)COC(=O)N[C@H](CC4=CSC5=CC=CC=C54)C(=O)O
124	C1[C@H](CN([C@@H]1C(=O)O)C(=O)OCC2C3=CC=CC=C3C4=CC=CC=C24)O
125	C1=CC=C2C(=C1)C(C3=CC=CC=C32)COC(=O)N[C@H](CC(=O)NC4C5=CC=CC=C5OC6=CC=CC=C46)C(=O)O
126	C[C@@H]1[C@H](N(C(O1)(C)C)C(=O)[C@H](CC2=CC=CC=C2)NC(=O)OCC3C4=CC=CC=C4C5=CC=CC=C35)C(=O)O
127	C1=CC=C(C(=C1)C[C@@H](C(=O)O)NC(=O)OCC2C3=CC=CC=C3C4=CC=CC=C24)C#N
128	C1=CC=C2C(=C1)C(C3=CC=CC=C32)COC(=O)N[C@@H](CC4=CNC5=CC=CC=C54)CC(=O)O
129	C1CC1C[C@H](C(=O)O)NC(=O)OCC2C3=CC=CC=C3C4=CC=CC=C24
130	C1CC[C@H]2[C@@H](C1)C[C@H](N2C(=O)OCC3C4=CC=CC=C4C5=CC=CC=C35)C(=O)O
131	C1CC1(CNC(=O)OCC2C3=CC=CC=C3C4=CC=CC=C24)C(=O)O
132	C1[C@H](N(CC2=C1C=CC(=C2)O)C(=O)OCC3C4=CC=CC=C4C5=CC=CC=C35)C(=O)O

Table S1. Continued from previous page.

Amino Acid	SMILES
133	CC1=CCC(CC1)(C(=O)O)NC(=O)OCC2C3=CC=CC=C3C4=CC=CC=C24
134	C1CCC(CC1)[C@@H]2C[C@H](N(C2)C(=O)OCC3C4=CC=CC=C4C5=CC=CC=C35)C(=O)O
135	C1=CC=C(C=C1)COCN2C=NC=C2C[C@@H](C(=O)O)NC(=O)OCC3C4=CC=CC=C4C5=CC=CC=C35
136	O=C(N[C@@H](CSS(O)(=O)=O)C(O)=O)OCC1C2=C(C3=C1C=CC=C3)C=CC=C2
137	C1=CC=C2C(=C1)C=CC=C2[C@H](CC(=O)O)NC(=O)OCC3C4=CC=CC=C4C5=CC=CC=C35
138	C1C(CC2=CC=CC=C21)[C@@H](C(=O)O)NC(=O)OCC3C4=CC=CC=C4C5=CC=CC=C35
139	O=C(O)[C@@H]1CCCN1C2CCN(C(OCC3C4=C(C5=C3C=CC=C5)C=CC=C4)=O)CC2
140	C[C@@H]([C@H](C(=O)O)NC(=O)OCC1C2=CC=CC=C2C3=CC=CC=C13)O
141	C[C@H]([C@@H](C(=O)O)NC(=O)OCC1C2=CC=CC=C2C3=CC=CC=C13)O
142	C1=CC=C2C(=C1)C(C3=CC=CC=C32)COC(=O)N[C@@H](CC4=CN=CN4)C(=O)O
143	C1=CC=C2C(=C1)C(C3=CC=CC=C32)COC(=O)N[C@H](CC4=CN=CN4)C(=O)O
144	C1=CC=C2C(=C1)C(C3=CC=CC=C32)COC(=O)N[C@@H](CC4=CNC5=CC=CC=C54)C(=O)O
145	C1=CC=C2C(=C1)C(C3=CC=CC=C32)COC(=O)N[C@H](CC4=CNC5=CC=CC=C54)C(=O)O
146	COC1=CC(=CC(=C1)[C@H](CC(=O)O)NC(=O)OCC2C3=CC=CC=C3C4=CC=CC=C24)OC
147	ClC1=CC(C[C@@H](C(O)=O)NC(OCC2C(C=CC=C3)=C3C4=C2C=CC=C4)=O)=CC=N1
148	C1=CC=C2C(=C1)C(C3=CC=CC=C32)COC(=O)NCC4=CC=CC(=C4)CC(=O)O
149	C1=CC=C2C(=C1)C(C3=CC=CC=C32)COC(=O)N[C@@H](CC4=CC=C(C=C4)Cl)C(=O)O
150	C1=CC=C2C(=C1)C(C3=CC=CC=C32)COC(=O)N[C@H](CO)C(=O)O
151	C1=CC=C2C(=C1)C(C3=CC=CC=C32)COC(=O)N[C@@H](CO)C(=O)O
152	C1=CC=C2C(=C1)C(C3=CC=CC=C32)COC(=O)N[C@H](CC4=CC=C(C=C4)O)C(=O)O
153	C1=CC=C2C(=C1)C(C3=CC=CC=C32)COC(=O)N[C@@H](CC4=CC=C(C=C4)O)C(=O)O
154	C1CC(CC2=CC=CC=C21)(C(=O)O)NC(=O)OCC3C4=CC=CC=C4C5=CC=CC=C35
155	C1=CC=C2C(=C1)C(C3=CC=CC=C32)COC(=O)N[C@@H](CS(=O)(=O)O)C(=O)O
156	CC1(N([C@@H](CO1)C(=O)O)C(=O)CNC(=O)OCC2C3=CC=CC=C3C4=CC=CC=C24)C
157	CC1(N([C@@H](CO1)C(=O)O)C(=O)[C@H](CC2=CC=CC=C2)NC(=O)OCC3C4=CC=CC=C4C5=CC=CC=C35)C
158	C1=CC=C2C(=C1)C(C3=CC=CC=C32)COC(=O)N[C@@H](CC4=CN=CC=C4)C(=O)O
159	C1CCC(CC1)C[C@@H]([C@H](CC(=O)O)O)NC(=O)OCC2C3=CC=CC=C3C4=CC=CC=C24
160	CC[C@H](C)[C@@H]([C@H](CC(=O)O)O)NC(=O)OCC1C2=CC=CC=C2C3=CC=CC=C13
161	C1=CC=C(C=C1)C=C(C(=O)O)NC(=O)OCC2C3=CC=CC=C3C4=CC=CC=C24
162	CC=C(C(=O)O)NC(=O)OCC1C2=CC=CC=C2C3=CC=CC=C13
163	C1=CC=C2C(=C1)C(C3=CC=CC=C32)COC(=O)N[C@@H](CCC4=CC=C(C=C4)O)C(=O)O
164	C1[C@H](CN([C@@H]1C(=O)O)C(=O)OCC2C3=CC=CC=C3C4=CC=CC=C24)OCC5=CC=CC=C5
165	C1=CC=C2C(=C1)C(C3=CC=CC=C32)COC(=O)NCC(C(=O)O)O
166	C1=CC=C2C(=C1)C(C3=CC=CC=C32)COC(=O)N[C@@H](CC4=CC=C(C=C4)N)C(=O)O
167	CC1=CC(=CC(=C1C[C@@H](C(=O)O)NC(=O)OCC2C3=CC=CC=C3C4=CC=CC=C24)C)O
168	C1=CC=C2C(=C1)C(C3=CC=CC=C32)COC(=O)N[C@H](CC4=CC(=C(C=C4)Cl)Cl)CC(=O)O
169	C1=CC=C2C(=C1)C(C3=CC=CC=C32)COC(=O)NCC4=CC=C(C=C4)C(=O)O
170	C1=CC=C2C(=C1)C(C3=CC=CC=C32)COC(=O)N[C@@H](CC4=CC(=C(C=C4)Cl)Cl)CC(=O)O
171	O=C([C@@H]1CC[C@@H](NC(OCC2C3=CC=CC=C3C4=CC=CC=C24)=O)CC1)O
172	C1=CC=C2C(=C1)C(C3=CC=CC=C32)COC(=O)N[C@H](CC4=CC(=C(C=C4)Cl)Cl)C(=O)O
173	OC(CC(C1)OCCN1C(OCC1c(cccc2)c2-c2c1cccc2)=O)=O
174	OC(c(cc1)ccc1N(CC1)CCC1NC(OCC1c(cccc2)c2-c2c1cccc2)=O)=O
175	OC(C(c1cccs1)NC(OCC1c(cccc2)c2-c2c1cccc2)=O)=O
176	OC(c1c(CNC(OCC2c(cccc3)c3-c3c2cccc3)=O)c(cccc2)c2s1)=O

Table S1. Continued from previous page.

Amino Acid	SMILES
177	OC(CN(C1CCCC1)C(OCC1c(cccc2)c2-c2c1cccc2)=O)=O
178	Cc1c(C(O)=O)sc(C(CC2)CCN2C(OCC2c(cccc3)c3-c3c2cccc3)=O)n1
179	Cc1cccc(CN(CC(O)=O)C(OCC2c(cccc3)c3-c3c2cccc3)=O)c1
180	OC(CC(C(CC1)CCN1C(OCC1c(cccc2)c2-c2c1cccc2)=O)c(cc1)ccc1F)=O
181	Cc(oc(CNC(OCC1c(cccc2)c2-c2c1cccc2)=O)c1)c1C(O)=O
182	OC(C1=NOC(C2)(CN2C(OCC2c(cccc3)c3-c3c2cccc3)=O)C1)=O
183	Cc1c(C(O)=O)sc(CCNC(OCC2c(cccc3)c3-c3c2cccc3)=O)n1
184	OC(CN(Cc(cc1)ccc1Cl)C(OCC1c(cccc2)c2-c2c1cccc2)=O)=O
185	OC(C(CC1)(CCN1C(OCC1c(cccc2)c2-c2c1cccc2)=O)n1nccc1)=O
186	OC(c1cccc(S(N(CC2)CCN2C(OCC2c(cccc3)c3-c3c2cccc3)=O)(=O)=O)c1)=O
187	OC(c1cc(CCN(C2)C(OCC3c(cccc4)c4-c4c3cccc4)=O)c2cc1)=O
188	OC(c(cc1)cc(C2)c1OCCN2C(OCC1c(cccc2)c2-c2c1cccc2)=O)=O
189	OC(C(CC1)(c2c1cccc2)NC(OCC1c(cccc2)c2-c2c1cccc2)=O)=O
190	COC1=CC(=C(C=C1)CN(CC(=O)O)C(=O)OCC2C3=CC=CC=C3C4=CC=CC=C24)O
191	OC(CN([C@@H](CC1)CS1(=O)=O)C(OCC1c(cccc2)c2-c2c1cccc2)=O)=O
192	O=C(O)[C@H](NC(OCC1C2=C(C3=C1C=CC=C3)C=CC=C2)=O)CC4=CC=CS4

Table S2. Library Carboxylic Acid SMILES

Carboxylic acid	SMILES
1	OC(c(cc1)cnc1OCC(F)(F)F)=O
2	OC(c(cc1)cc(CCC2)c1C2=O)=O
3	OC(c1cc(-c2ccncc2)nc2ccccc12)=O
4	OC(c1cc(-c2cnccc2)nc2ccccc12)=O
5	OC([C@H](C1)[C@@H]1c1ccccc1)=O
6	OC(C1(CC(C2)C3)CC3CC2C1)=O
7	OC(C1CC1)=O
8	OC(c(cc1)cc2c1OCC2)=O
9	OC(c1csc(-c2ccncc2)n1)=O
10	OC(c1c(C(F)(F)F)ccnc1)=O
11	OC(c1c[nH]c(cc2)c1cc2Cl)=O
12	OC(c1nccnc1Cl)=O
13	Cc1nc2ccncc2cc1C(O)=O
14	CC(C)(CC(O)=O)n1cccc1
15	OC(c1cc2nsnc2cc1)=O
16	OC(c(s1)ccc1Cl)=O
17	Cc1cc(C(O)=O)c(C(F)(F)F)o1
18	OC(C=C(C(F)(F)F)C(F)(F)F)=O
19	OC(C(CC1)CCN1C(c1ccccc1)=O)=O
20	OC(c1cc(CCCC2)c2cc1)=O
21	Cc(cc1C)cc2c1OC(C(O)=O)=CC2=O
22	OC(c(cccc1)c1Nc1cccc(F)c1)=O
23	OC(c1ccc(-c2ncccc2)s1)=O
24	OC(C(C1)CN1C(c1ccccc1)c1ccccc1)=O
25	OC(c(cc1)cc2c1OCCO2)=O
26	OC(c1cc(scc2)c2s1)=O
27	OC(c1c(C(F)(F)F)n(-c2ccccc2)nc1)=O
28	NC(c(cc1)ccc1C(O)=O)=O
29	CN(CC1)CCC1C(O)=O
30	OC(c1csc(-c2cnccc2)n1)=O
31	N#Cc(cc1)ncc1C(O)=O
32	OC(c1cc(cc(cc2)Cl)c2o1)=O
33	OC(CCc1ccncc1)=O
34	OC(C1(CCC1)C(F)(F)F)=O
35	OC(c1cnc(ccc(C(F)(F)F)c2)c2c1O)=O
36	Cc(cc1)cc2c1OC(C(O)=O)=CC2=O
37	CC(C)(C)C(O)=O
38	CS(c1ccc(/C=C/C(O)=O)cc1)(=O)=O
39	OC(C1=CCc2c1cccc2)=O
40	OC(c1cccc(O2)c1OC2(F)F)=O
41	CCOc1cccc2c1oc(C(O)=O)c2
42	OC(c1cc(-c2ncccc2)nc2ccccc12)=O
43	CC(C)Oc(ccc(C(O)=O)c1)c1OC
44	OC(C1=CC=C(C(F)(F)F)NC1=O)=O

Table S2. Continued from previous page.

Carboxylic acid	SMILES
45	Cc1cccn2c1nc(C(O)=O)c2
46	OC(/C=C/c(cc1)cc2c1OCC2)=O
47	CC(C(C(C)=O)c(cccc1)c1C(O)=O)=O
48	OC(c1ccc(CN2CCCCC2)o1)=O
49	Cc(cc1)cc2c1OC=C(C(O)=O)C2=O
50	Nc1nc(ccc(CC(O)=O)c2)c2s1
51	OC(c1cnc(CCN2CCCC2)cc1)=O
52	OC(C1CCN(Cc2cccc(F)c2)CC1)=O
53	OC(c(cc1)cc(C(c2c3cccc2)=O)c1C3=O)=O
54	OC(Cc1c[nH]c2c1ccc(F)c2)=O
55	OC(c1cc2cccnc2nc1C(F)(F)F)=O
56	CC(C)([C@@H](CC(O)=O)C1)[C@@H]1C(C)=O
57	CC(C)[C@H](CC1)CC[C@@H]1C(O)=O
58	Cc1nc(C(O)=O)c(C(F)(F)F)o1
59	OC(c1cc(cc(C(F)(F)F)cc2)c2s1)=O
60	OC(c(cc1)cc2c1OCO2)=O
61	OC(Cc(cc1)cc2c1OCO2)=O
62	OC(c(cc1)ccc1O)=O
63	Cc1nc(C(O)=O)cs1
64	OC(c(cc1)ccc1-c1ncccc1)=O
65	Nc(nccc1)c1C(O)=O
66	OC([C@@H](CC1)OC1=O)=O
67	OB(c(cccc1)c1C(O)=O)O
68	OB(c1ccc(C(O)=O)c2ccccc12)O
69	OB(c1cccc(/C=C/C(O)=O)c1)O
70	OB(c1csc(C(O)=O)c1)O
71	OB(c(cc1)ccc1C(O)=O)O
72	OB(c1cccc(C(O)=O)c1)O
73	NC(c(cc1)ccc1C(O)=O)=N
74	CCN(C=C1C(O)=O)c(cc(C)cc2)c2C1=O
75	OC(c1ncccc1)=O
76	CCC(O)=O
77	COc(cc(cc1)N)c1C(O)=O
78	OC(c1cnco1)=O
79	CC1(COC1)C(O)=O
80	OC(C1(CC1)c1cccs1)=O
81	OC([C@H](CC1)OC1=O)=O
82	OC([C@H](CS1)NC1=O)=O
83	OC(c1cccc2nccnc12)=O
84	OC(c1cc(-c2ccccc2)n[nH]1)=O
85	OC(c1cccc(Cl)n1)=O
86	Cc1nocc1C(O)=O
87	OC(c1nsnc1)=O

Table S2. Continued from previous page.

Carboxylic acid	SMILES
88	OC(c1cc(c(OCc2ccccc2)ccc2)c2[nH]1)=O
89	OC(c1noc(-c2ccccc2)c1)=O
90	OC(C1CCOCC1)=O
91	OC(c(cc1)cc2c1[nH]nc2)=O
92	OC(/C=C/c(cc1)cc(O)c1O)=O
93	OC(CCc(ccc(Cl)c1)c1Cl)=O
94	OC(c1ccno1)=O
95	OC(c1cccs1)=O
96	OC([C@@H]1OCCC1)=O
97	OC([C@H]1OCCC1)=O
98	CCOCC(O)=O
99	OC(c(o1)ccc1Cl)=O
100	OC(c1nc2ccccc2nc1)=O
101	OC(c1ccns1)=O
102	OC(c1cc(O)ccc1)=O
103	OC(c(cc1)cc2c1ocn2)=O
104	CO[C@H](C(O)=O)c1ccccc1
105	OC(CCN1CCOCC1)=O
106	OC(c1csc(Cc2ccccc2)n1)=O
107	OC(c(nc1)cn1-c1ccccc1)=O
108	OC(c(cc1)ncc1O)=O
109	OC(c1ccc(CN2CCCC2)o1)=O
110	CCc1c(C(O)=O)snn1
111	CCn(c(C(O)=O)c1)c2c1cccc2
112	Cc1c(C(O)=O)c(-c2ccccc2)no1
113	OC(c1cc(O)cs1)=O
114	Cn(cc1)nc1C(O)=O
115	OC(c(scc1)c1-n1cccc1)=O
116	Cn(cc1)c(cc2)c1cc2C(O)=O
117	CC(C)c1c(C(O)=O)ncs1
118	CN(CC1)CCC1=CC(O)=O
119	OC(CON=C1CCCCCC1)=O
120	OC(c1cc(-c2ccccc2)no1)=O
121	OC(c1cn(CCCC2)c2n1)=O
122	OC(C=C1CCOCC1)=O
123	OC(c1c(CCCC2)c2ncc1)=O
124	OC([C@@H](CCC1)CN1C(c(cc1)ccc1F)=O)=O
125	Cc1cc(C(O)=O)c(C)n1C1CC1
126	Cc1nc(cc(cc2)C(O)=O)c2n1C1CC1
127	CN(C)C(c1cccc(C(O)=O)c1)=O
128	OC(c1nn(CCCC2)c2c1)=O
129	OC(CN(c1c(CCC2)scc1)C2=O)=O
130	OC(c1cc(-n2nccc2)ccc1)=O

Table S2. Continued from previous page.

Carboxylic acid	SMILES
131	Cc1nc(-c2ccc(CC(O)=O)cc2)cs1
132	OC(c(cc1)ccc1N(CCC1)C1=O)=O
133	N#Cc(cccc1)c1N(C1)CC1C(O)=O
134	CN(C)c1cc(C(O)=O)ncc1
135	Cc(scc1)c1C(O)=O
136	OC(c1nccc(C(F)(F)F)c1)=O
137	OC(c1cc(N2CC=CC2)ccc1)=O
138	Cc1nc(ccc(C(O)=O)c2)c2s1
139	OC(c1cccnc1SC1CCCC1)=O
140	Cn1ncc(C(O)=O)c1C(F)(F)F
141	Cc(cc1C(O)=O)nn1-c1ccccc1
142	OC(Cc1cc(CCC2)c2cc1)=O
143	CC(C)(C)n1ncc(C(O)=O)c1
144	COc1ccc(C(O)=O)c(Cl)n1
145	[O-]C(c1nc(cccc2)c2s1)=O.[K+]
146	OC(c(cc1)ccc1-c1cscc1)=O
147	OC(c(cc1)ccc1N1CCCCC1)=O
148	OC(c(cccc1)c1OC1CCCC1)=O
149	OC(c(cc1)cc2c1scn2)=O
150	OC(c1ccc(-c2cccs2)s1)=O
151	OC(c(cc1)cc(F)c1N1CCCC1)=O
152	OC(c(cc1)cc(F)c1N1CCOCC1)=O
153	OC(c1cccc(Oc2nccs2)c1)=O
154	Cc1c(C(O)=O)n(cccc2)c2n1
155	OC(c(cc1)cnc1Oc(cccc1)c1F)=O
156	OC(c1cn(CC2CCCCC2)nc1)=O
157	Cn1nc(C(F)F)c(C(O)=O)c1
158	OC(c1csc2c1cccc2)=O
159	OC(c(cc1)c(C(F)(F)F)cc1Cl)=O
160	CC(C)(C1)[C@H]1C(O)=O
161	CC(C)Sc1ncccc1C(O)=O
162	OC(CN(C(CS1)=O)C1=S)=O
163	Cc(ccc(C(O)=O)c1)c1N
164	OC(Cc1csc(-c2ccccc2)n1)=O
165	OC(Cc1cscc1)=O
166	OC(c1ccc(C(F)(F)F)nc1Cl)=O
167	OC(c(cc1)ccc1-n1nccc1)=O
168	OC(CSC(N1CCCC1)=S)=O
169	Cc1c(C(O)=O)n(ccs2)c2n1
170	OC(c1noc(C2CC2)c1)=O
171	OC(C1(CC1)c1ccccc1)=O
172	OC(c1cncs1)=O
173	OC(c1cccc(N(CCC2)C2=O)c1)=O

Table S2. Continued from previous page.

Carboxylic acid	SMILES
174	OC(c1ccc(Oc2ccccc2)o1)=O
175	OC(CSc1ccncc1)=O
176	Cc1c(CC(O)=O)sc(S)n1
177	Cc1c(C(O)=O)sc2c1cccc2
178	OC(c1cnc2n1CCCC2)=O
179	OC(c1cscn1)=O
180	OC(Cn1c(cccc2)c2nc1C(F)(F)F)=O
181	OC(C1(CCCC1)c1ccccc1)=O
182	Cc(ccc(F)c1)c1C(O)=O
183	CN(C)c(cc1)ncc1C(O)=O
184	Cc(onc1)c1C(O)=O
185	Cc(c(F)c1C(O)=O)ccc1F
186	CN(CCC1)[C@H]1C(O)=O
187	CC(C)c1nc(C)c(C(O)=O)s1
188	OC(c1cc2ccnn2cc1)=O
189	CN(C)c(cc1)cc(F)c1C(O)=O
190	OC(c1ccc(C2SCCS2)cc1)=O
191	Cc(nc(cc1)-c2cccs2)c1C(O)=O
192	OC(c1cn(cc(cc2)Cl)c2n1)=O
193	Cc1c(C(O)=O)sc(Cc(cc2)ccc2F)n1
194	OC(CSc1nncs1)=O
195	OC([C@H](CSC1)N1C(C1CCCC1)=O)=O
196	OC(c(cccc1)c1C(c1cccs1)=O)=O
197	OC(C1(CCOCC1)c1ccccc1)=O
198	CSc1nc(C(O)=O)c2n1cccc2
199	OC(c(cc1)ccc1N(C(CS1)=O)C1=S)=O
200	OC(C(C1)CC1=O)=O
201	OC(c1c(-n2cccc2)sc2c1CCCC2)=O
202	CSc1nccn1-c1cc(C(O)=O)ccc1
203	OC(c(cc1)cc2c1occ2)=O
204	OC(c1c(CCCC2)c2ccc1)=O
205	OC(c1ccc(CN2CCOCC2)cc1)=O
206	OC(c1cccn2c1ncc2)=O
207	OC(C1=CCCCO1)=O
208	CC(C)[C@H](C(O)=O)O
209	CC(C1(CC1)C(O)=O)=O
210	C[C@H](C(O)=O)N(Cc1c2cccc1)C2=O
211	CC[C@@H](C(O)=O)N(C)C
212	C[C@@H](C(O)=O)c1ccccc1
213	CC(C)[C@@H](C(O)=O)N(Cc1c2cccc1)C2=O
214	CN(CCC1)[C@@H]1C(O)=O
215	CC(C)[C@H](C(O)=O)n1cccc1
216	C[C@@H](c1ccccc1)N(C[C@@H](C1)C(O)=O)C1=O

Table S2. Continued from previous page.

Carboxylic acid	SMILES
217	CC(N(CSC1)[C@@H]1C(O)=O)=O
218	OC(c(sc(N1CCCC1)n1)c1Cl)=O
219	O[C@](C1)(C[C@H]1C(O)=O)C(F)(F)F
220	C[C@@H](C(O)=O)N(C)C
221	C[C@](C(F)(F)F)(C(O)=O)O
222	C[C@H](C(O)=O)Oc(cc1)ccc1O
223	COc(cc1)cnc1C(O)=O
224	OC(c1cc(C(N2CCCCC2)=O)ccc1)=O
225	Cc1nc(cc(cc2)C(O)=O)c2n1C
226	Cc1cc(C(O)=O)c(-n2cccc2)s1
227	OC(c1noc(-c2cccs2)c1)=O
228	OC(CN1c(cccc2)c2SCC1=O)=O
229	CC(CC1)=NN1c(cc1)ccc1C(O)=O
230	OC(c1cc(N2CCCC2)ccc1)=O
231	OC(c(cc1)ccc1S(C1CCCC1)(=O)=O)=O
232	OC(c(cc1)ccc1Oc(cc1)ccc1F)=O
233	OC(c(n(ccs1)c1n1)c1Cl)=O
234	OC(c1c(-c2ccccc2)onc1)=O
235	Cc1c(CC(O)=O)c(C)nn1-c1ccccc1
236	CN(C)c(c1ccccc1nc1)c1C(O)=O
237	O[C@@H](C(O)=O)c(cccc1)c1Cl
238	Cc(nc(C1CC1)nc1)c1C(O)=O
239	OC(C(C1)Cc2c1cccc2)=O
240	Cc(n(-c1ccccc1)nc1)c1C(O)=O
241	OC(C1(CCCC1)c(cc1)ccc1F)=O
242	OC(CN(C1)Cc2c1cccc2)=O
243	OC(c1csc(C(F)(F)F)n1)=O
244	Cc1cc(C)nn1-c1cccc(C(O)=O)c1
245	OC(c1cccn1Cc1cccs1)=O
246	OC(c1ccc(C2SCCCS2)cc1)=O
247	OC(CN(CCS1)C1=O)=O
248	OC(C1=CCCC1)=O
249	OC(c1cc(CCCC2)c2s1)=O
250	OC(c1csc(-c2ccco2)n1)=O
251	CC(C)c1c(C(O)=O)nco1
252	OC(c(cc1)ccc1-n1cccc1)=O
253	OC(Cc1csc(-n2cccc2)n1)=O
254	OC(c(cccc1)c1Oc1cnccc1)=O
255	OC(c1cc(Cn2nccc2)ccc1)=O
256	OC(c1n[nH]c2c1CCCC2)=O
257	OC(c(cccc1)c1C(N1CCCCC1)=O)=O
258	OC(c(cc1)cc2c1nc(C1CC1)o2)=O
259	Cc1nn(C)c(C(O)=O)c1

Table S2. Continued from previous page.

Carboxylic acid	SMILES
260	OC(C=C1CCCC1)=O
261	OC(c(cc1)ccc1-c1csnn1)=O
262	OC(C=C1CCCCC1)=O
263	OC(CN1CCCCC1)=O
264	N=C1SC=CN1CC(O)=O
265	COc(ccnc1)c1C(O)=O
266	OC(c(cc1)ccc1N1CCOCC1)=O
267	OC(c(cccc1)c1N(CCC1)C1=O)=O
268	OC(CC1=CSC2=NCCN12)=O
269	OC(c1csc(C2CCCCC2)n1)=O
270	CC(N(C)C=C1C(O)=O)=CC1=O
271	OC(c1ccc(CN(CC2)c3c2cccc3)cc1)=O
272	CC(C)Oc(cc1)ncc1C(O)=O
273	CC(C)c1c(C(O)=O)c(C)no1
274	OC(C1=Cc(cccc2)c2OC1=O)=O
275	Cn1c(SCC(O)=O)ncc1
276	OC(C1=COCC1)=O
277	Cc1ccc(C)n1-c(cc1)ccc1C(O)=O
278	OC(c(cc1)ccc1Oc1ncccc1)=O
279	CC(C)n(cc1)c2c1ccc(C(O)=O)c2
280	OC(c1c(C2CC2)nns1)=O
281	CC(CC1)CCN1c(cc1)ccc1C(O)=O
282	OC(c1cn(C2CCCC2)nc1)=O
283	OC([C@H]1N=C(c2ccccc2)SC1)=O
284	OC(c1cc(OC2CCCCC2)ncc1)=O
285	OC(c(cccc1)c1N1CCCC1)=O
286	Cn(c(C1CC1)c1)nc1C(O)=O
287	OC(c1csc2c1CCCC2)=O
288	CC(C)=CC(O)=O

Table S3. Library synthesis validation. *indicates a synthesis truncate was observed. Mismatches are colored red.

Compound ID	Structure	Observed m/z (expected m/z)
QC-1	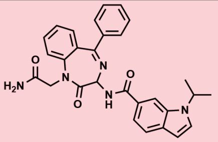	1340.52(1483.64 [M+H]$^+$)
QC-2	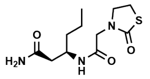	1287.55 (1287.54 [M+Na]$^+$)
QC-3	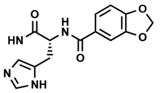	1292.54 (1292.53 [M+H]$^+$)
QC-4	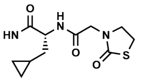	1261.60 (1261.52 [M+H]$^+$)
QC-5	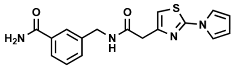	1330.63 (1330.52 [M+H]$^+$)
QC-6	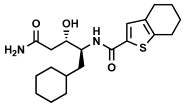	1171.56* (1368.62 [M+H]$^+$)
QC-7	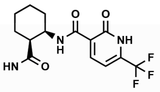	1321.80 (1321.54 [M+H]$^+$)
QC-8	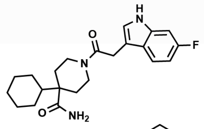	1182.73* (1375.54 [M+H]$^+$)
QC-9	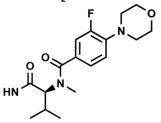	1327.83 (1327.60 [M+H]$^+$))
QC-10	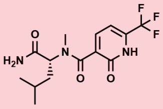	1196.64* (1323.55 [M+H]$^+$)

Table S3. Continued from previous page.

Compound ID	Structure	Observed m/z (expected m/z)
QC-11	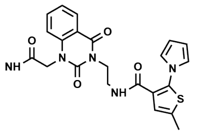	1473.38 (1473.59 [M+MeOH+H]$^+$)
QC-12	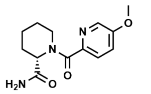	1253.78 (1253.55 [M+H]$^+$)
QC-13	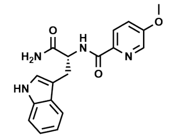	1328.78 (1328.56 [M+H]$^+$)
QC-14	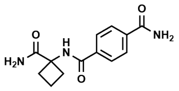	1251.76 (1251.54 [M+H]$^+$)
QC-15	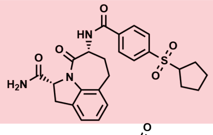	1356.87 (1471.59 [M+H]$^+$)
QC-16	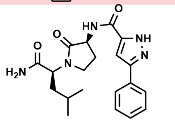	1373.91 (1373. 62 [M+H]$^+$)
QC-17	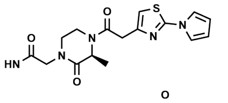	1351.84 (1351.55 [M+H]$^+$)
QC-18	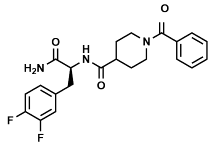	1405.87 (1405.60 [M+H]$^+$)
QC-19	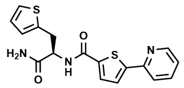	1347.54 (1347.48 [M+H]$^+$)
QC-20	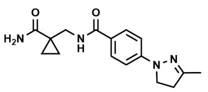	1288.76(1290.58 [M+H]$^+$)

Table S3. Continued from previous page.

Compound ID	Structure	Observed m/z (expected m/z)
QC-21	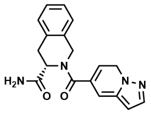	1310.75 (1311.56 [M+H]+)
QC-20	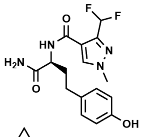	1342.85 (1342.56 [M+H]+)
QC-23	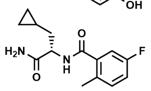	1254.81 (1254.55 [M+H]+)

Table S4. Summary of FXa droplet screening.

Screen	UV Dose	Time (min)	Droplets	Beads	Eq	Hits	Hit Rate (%)
1	100	112	351,857	89,600	1.6	352	0.10
2	100	95	314,418	76,000	1.4	198	0.06
3	100	113	406,800	90,400	1.6	363	0.09
4	100	132	481,193	105,600	1.9	354	0.07
5	100	90	251,524	54,000	1.0	116	0.05
6	100	162	486,446	97,200	1.8	474	0.10
1	30	149	461,593	119,200	2.2	124	0.03
2	30	123	398,959	98,400	1.8	109	0.03
3	30	209	603,148	167,200	3.0	352	0.06
4	30	171	481,193	136,800	2.5	113	0.02
5	30	203	585,248	121,800	2.2	354	0.06
6	30	125	366,451	75,000	1.4	289	0.08
1	0	14	43,714	11,200	0.2	12	0.03
2	0	32	104,391	25,600	0.5	10	0.01
3	0	32	101,205	25,600	0.5	24	0.02
5	0	33	94,198	19,800	0.4	5	0.01
6	0	30	90,568	18,000	0.3	10	0.01

Eq	Hit Rate (%)
9.3	0.08 ± 0.01
13.0	0.05 ± 0.01
1.8	0.020 ± 0.004

Table S5. Sequencing barcoding primers.

Primer	Sequence
**F001**	AATGATACGGCGACCACCGAGATCTACACTCTTTCCCTACACGACGCTCTTCCGATCTGCCGCCGCCTTCGTCCTTCTCAGCGAC
**R001**	CAAGCAGAAGACGGCATACGAGAT**ATTGGC**GTGACTGGAGTTCAGACGTGTGCTCTTCCGATCTGTGGCACAACAACTGGCGGGCAAAC
**R002**	CAAGCAGAAGACGGCATACGAGAT**GATCTG**GTGACTGGAGTTCAGACGTGTGCTCTTCCGATCTGTGGCACAACAACTGGCGGGCAAAC
**R003**	CAAGCAGAAGACGGCATACGAGAT**TCAAGT**GTGACTGGAGTTCAGACGTGTGCTCTTCCGATCTGTGGCACAACAACTGGCGGGCAAAC
**R004**	CAAGCAGAAGACGGCATACGAGAT**CTGATC**GTGACTGGAGTTCAGACGTGTGCTCTTCCGATCTGTGGCACAACAACTGGCGGGCAAAC
**R005**	CAAGCAGAAGACGGCATACGAGAT**AAGCTA**GTGACTGGAGTTCAGACGTGTGCTCTTCCGATCTGTGGCACAACAACTGGCGGGCAAAC
**R006**	CAAGCAGAAGACGGCATACGAGAT**GTAGCC**GTGACTGGAGTTCAGACGTGTGCTCTTCCGATCTGTGGCACAACAACTGGCGGGCAAAC
**R007**	CAAGCAGAAGACGGCATACGAGAT**TACAAG**GTGACTGGAGTTCAGACGTGTGCTCTTCCGATCTGTGGCACAACAACTGGCGGGCAAAC
**R008**	CAAGCAGAAGACGGCATACGAGAT**TTGACT**GTGACTGGAGTTCAGACGTGTGCTCTTCCGATCTGTGGCACAACAACTGGCGGGCAAAC
**R009**	CAAGCAGAAGACGGCATACGAGAT**GGAACT**GTGACTGGAGTTCAGACGTGTGCTCTTCCGATCTGTGGCACAACAACTGGCGGGCAAAC
**R010**	CAAGCAGAAGACGGCATACGAGAT**TGACAT**GTGACTGGAGTTCAGACGTGTGCTCTTCCGATCTGTGGCACAACAACTGGCGGGCAAAC
**R011**	CAAGCAGAAGACGGCATACGAGAT**GGACGG**GTGACTGGAGTTCAGACGTGTGCTCTTCCGATCTGTGGCACAACAACTGGCGGGCAAAC
**R012**	CAAGCAGAAGACGGCATACGAGAT**GCGGAC**GTGACTGGAGTTCAGACGTGTGCTCTTCCGATCTGTGGCACAACAACTGGCGGGCAAAC

Table S6. Comparison of FXa AA hits and known inhibitors.

Series Rank	Hit Series	Representative Hit	Known Inhibitor	Reference
1	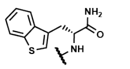	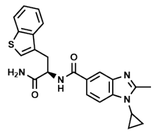	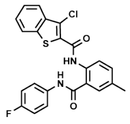	**Chou 2003**[11]
2	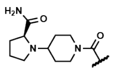	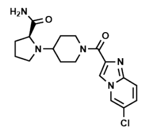	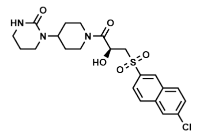	**Fujimoto 2010**[12]
4	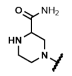	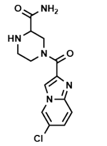	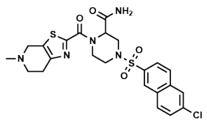	**Haginoya 2004**[13]

Table S7. Comparison of FXa COOH hits and known inhibitors.

Series Rank	Hit Series	Representative Hit	Known Inhibitor	Reference
**1**	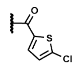	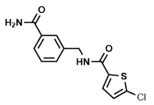	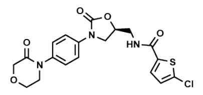 **Rivaroxiban**	**Roehrig 2005[14]**
**2**	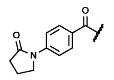	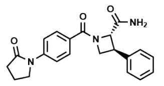	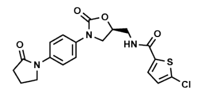	**Roehrig 2005[14]**
			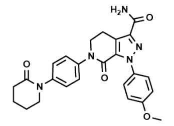 **Apaxiban**	**Pinto 2007[15]**
**3**	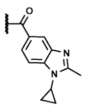	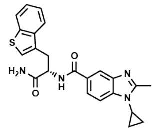	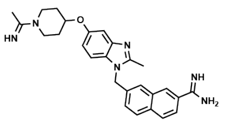	**Zhao 2000[16]**
**5**	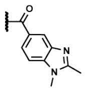	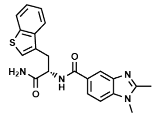		

Table S8. Summary of ATX droplet screening.

Screen	UV Dose	Time (min)	Droplets	Beads	Eq	Hits	Hit Rate (%)
1	100	125	350,000	100,000	1.8	918	0.26
2	100	80	210,000	64,000	1.2	586	0.28
4	100	105	320,000	63,000	1.1	697	0.22
5	100	90	260000	54,000	1	539	0.21
3	20	125	330000	100,000	1.8	319	0.10
4	20	210	640000	126,000	2.3	474	0.07
5	20	165	470000	99,000	1.8	369	0.08

Eq	Hit Rate (%)
5.1	0.24 ± 0.02
5.9	0.08 ± 0.01

Table S9. Comparison of ATX AA hits and known inhibitors.

Series Rank	Hit Series	Representative Hit	Known Inhibitor	Reference
1	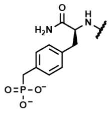	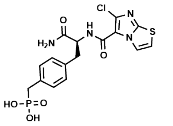	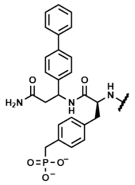	Cuozzo 2020[17]
3	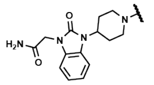	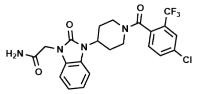	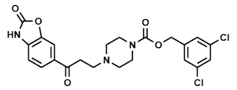	Gierse 2010[18]
5	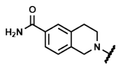	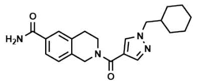	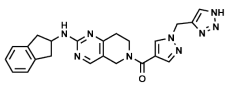	Beauchamp 2012[19]
			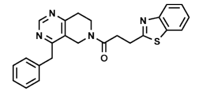	Long 2012[20]
6	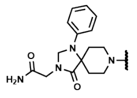	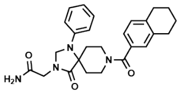	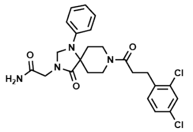	Cochrane 2019[4]
			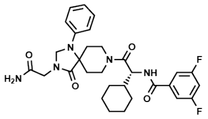	Cuozzo 2020[17]
10	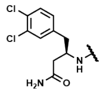	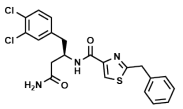	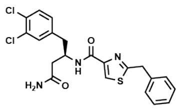	Cochrane 2019[4]

Table S10. Comparison of ATX COOH hits and known inhibitors.

Series Rank	Hit Series	Representative Hit	Known Inhibitor	Reference
**1**	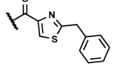	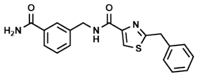	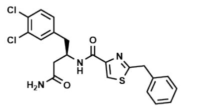	**Cochrane 2019[4]**
**2**	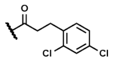	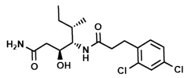	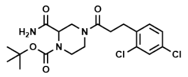	**Cochrane 2019[4]**
**3**	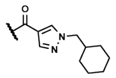	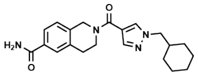	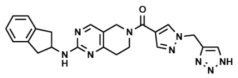	**Gierse 2006[18]**

Table S11. Oligonucleotide decoding table.

Overhang ID	Overhang [+]	Overhang [-]
≈X1XX [+]	/5Phos/ATGG	/5Phos/TGA
≈X2XX[+]	/5Phos/TCA	/5Phos/AAC
≈X3XX[+]	/5Phos/GTT	/5Phos/TAG
≈X4XX[+]	/5Phos/CTA	/5Phos/GAA
≈X5XX[+]	/5Phos/TTC	/5Phos/GCG
≈X6XX[+]	/5Phos/CGC	/5Phos/AGGC

PCR Primer ID	PCR Primer [+]	PCR Primer [-]
≈0002	/5Phos/GCCGCCGCCTTCGTCCTTCT CAGCGAC	/5Phos/CCATGTCGCTGAGAA GGACGAAGGCGGCGGCGG
≈0B02	/5Phos/GCCTCCCAAACNNNNNNNNG TTTGCCCGCCAGTTGTTGTGCCAC	GTTTGGG

Table S11. Continued from previous page.

Code 1 ID	Code 1 [+]	Code 1 [-]	Code 2 ID	Code 2 [+]	Code 2 [-]
≈1X01	TGGAAAGT	ACTTTCCA	≈2X01	CCTCCTAA	TTAGGAGG
≈1X02	ACGGAGCA	TGCTCCGT	≈2X02	AACCTCAA	TTGAGGTT
≈1X03	TTGGAGTT	AACTCCAA	≈2X03	AATCCCAT	ATGGGATT
≈1X04	AAGGAGGT	ACCTCCTT	≈2X04	AACCCTAC	GTAGGGTT
≈1X05	AGAAAGCA	TGCTTTCT	≈2X05	ATCCTCTC	GAGAGGAT
≈1X06	ACAGAACT	AGTTCTGT	≈2X06	CATTTCAA	TTGAAATG
≈1X07	TAAGGAGT	ACTCCTTA	≈2X07	CGCCTTCA	TGAAGGCG
≈1X08	ATGGGAGT	ACTCCCAT	≈2X08	CGTTCCTG	CAGGAACG
≈1X09	TGAAGGAA	TTCCTTCA	≈2X09	TTCTTCAT	ATGAAGAA
≈1X10	TTGAGGAT	ATCCTCAA	≈2X10	TCCTCTTA	TAAGAGGA
≈1X11	AAAGAAAC	GTTTCTTT	≈2X11	AACCTTCG	CGAAGGTT
≈1X12	AAAGGACA	TGTCCTTT	≈2X12	AACTCCCG	CGGGAGTT
≈1X13	AAAGGGAT	ATCCCTTT	≈2X13	AACTCTTT	AAAGAGTT
≈1X14	AAGAAACC	GGTTTCTT	≈2X14	AATCCTCA	TGAGGATT
≈1X15	AAGAGGCA	TGCCTCTT	≈2X15	AATCTCCC	GGGAGATT
≈1X16	ACAAAGAC	GTCTTTGT	≈2X16	AATCTTGT	ACAAGATT
≈1X17	ACAAGAAA	TTTCTTGT	≈2X17	AATTCCGA	TCGGAATT
≈1X18	ACAAGGCT	AGCCTTGT	≈2X18	ACCCTCCT	AGGAGGGT
≈1X19	ACAGGGTA	TACCCTGT	≈2X19	ACCCTTGA	TCAAGGGT
≈1X20	ACGAAAGA	TCTTTCGT	≈2X20	ACCTCCAA	TTGGAGGT
≈1X21	ACGAGATT	AATCTCGT	≈2X21	ACCTCTCC	GGAGAGGT
≈1X22	ACGAGGGC	GCCCTCGT	≈2X22	ACCTTCGC	GCGAAGGT
≈1X23	ACGGAATC	GATTCCGT	≈2X23	ACTCCCGC	GCGGGAGT
≈1X24	ACGGGAAG	CTTCCCGT	≈2X24	ACTCCTTT	AAAGGAGT
≈1X25	AGAAGACC	GGTCTTCT	≈2X25	ACTTCCCT	AGGGAAGT
≈1X26	AGGAAGGG	CCCTTCCT	≈2X26	AGCCCTCT	AGAGGGCT
≈1X27	AGGGAAAT	ATTTCCCT	≈2X27	AGCTCCTC	GAGGAGCT
≈1X28	ATAAGGGA	TCCCTTAT	≈2X28	AGTCTCTA	TAGAGACT
≈1X29	ATAGAGCC	GGCTCTAT	≈2X29	AGTTCTGT	ACAGAACT
≈1X30	CAAAGACT	AGTCTTTG	≈2X30	AGTTTCAT	ATGAAACT
≈1X31	CAAAGGAC	GTCCTTTG	≈2X31	ATCCCTTA	TAAGGGAT
≈1X32	CAAGAAGA	TCTTCTTG	≈2X32	ATCTCCGT	ACGGAGAT
≈1X33	CAAGAGTC	GACTCTTG	≈2X33	ATCTTCCA	TGGAAGAT
≈1X34	CAGAAGGA	TCCTTCTG	≈2X34	ATTCTCGG	CCGAGAAT
≈1X35	CAGAGAAA	TTTCTCTG	≈2X35	ATTTCCAC	GTGGAAAT
≈1X36	CAGGGACG	CGTCCCTG	≈2X36	CACCCTGA	TCAGGGTG
≈1X37	CCGAAACT	AGTTTCGG	≈2X37	CACCTCGC	GCGAGGTG
≈1X38	CCGAGGAG	CTCCTCGG	≈2X38	CACCTTAT	ATAAGGTG
≈1X39	CCGGAGGG	CCCTCCGG	≈2X39	CACTCCAT	ATGGAGTG
≈1X40	CGAGAACC	GGTTCTCG	≈2X40	CATCCCTA	TAGGGATG
≈1X41	CGAGGAGG	CCTCCTCG	≈2X41	CCCTCCGG	CCGGAGGG
≈1X42	CGAGGGCA	TGCCCTCG	≈2X42	CCCTTCTA	TAGAAGGG
≈1X43	CGGGAATA	TATTCCCG	≈2X43	CCCTTTCG	CGAAAGGG
≈1X44	CGGGAGCT	AGCTCCCG	≈2X44	CCTCTCAT	ATGAGAGG

Table S11. Continued from previous page.

≈1X45	CTGAAGCC	GGCTTCAG	≈2X45	CCTTTCCC	GGGAAAGG
≈1X46	CTGGAAAC	GTTTCCAG	≈2X46	CGCTCCCA	TGGGAGCG
≈1X47	GAGAGGGT	ACCCTCTC	≈2X47	CGTCCCAC	GTGGGACG
≈1X48	GAGGAACA	TGTTCCTC	≈2X48	CGTCCTGG	CCAGGACG
≈1X49	GAGGGAAT	ATTCCCTC	≈2X49	CGTCTCCG	CGGAGACG
≈1X50	GCAAAGGG	CCCTTTGC	≈2X50	CTCCCTCG	CGAGGGAG
≈1X51	GCAGAGAA	TTCTCTGC	≈2X51	CTTCCCGT	ACGGGAAG
≈1X52	GCAGGACC	GGTCCTGC	≈2X52	GACTCCGC	GCGGAGTC
≈1X53	GCGGAAGT	ACTTCCGC	≈2X53	GCCCTCGG	CCGAGGGC
≈1X54	GCGGGATA	TATCCCGC	≈2X54	GCCCTTCC	GGAAGGGC
≈1X55	GGAAGAGA	TCTCTTCC	≈2X55	GCCTCCTT	AAGGAGGC
≈1X56	GGAGAGGT	ACCTCTCC	≈2X56	GCTCCCTG	CAGGGAGC
≈1X57	GGAGGATT	AATCCTCC	≈2X57	GGCCCTAA	TTAGGGCC
≈1X58	GGAGGGAC	GTCCCTCC	≈2X58	GGCTCTCG	CGAGAGCC
≈1X59	GGGAAACG	CGTTTCCC	≈2X59	GGCTTCCC	GGGAAGCC
≈1X60	GGGAAGAA	TTCTTCCC	≈2X60	GGTCCCGA	TCGGGACC

Supporting Figures

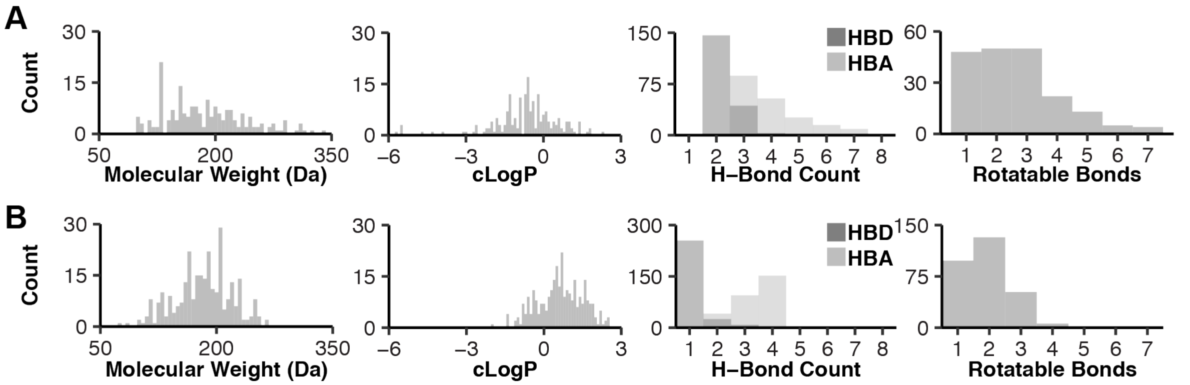

Figure S1. DEL building block properties. (A) Amino acids building blocks were modified *in silico* to replace N-Fmoc with an N-H group, and the acid was converted to a primary amide. Properties for the 192 amino acids in the library were then calculated. (B) Carboxylic acid building blocks were modified *in silico* to convert the carboxylic acid to a primary amide. Properties for the 288 carboxylic acids in the library were calculated.

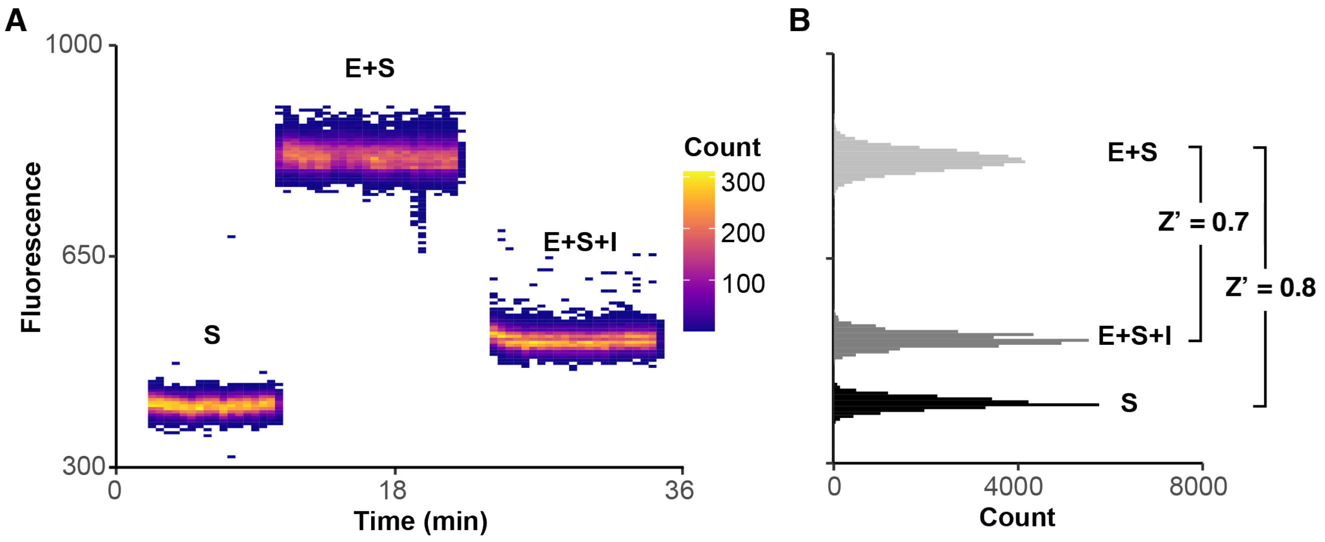

Figure S2. Droplet-scale FXa activity assay validation. (A) Droplets were generated with fluorogenic peptide substrate only (S, 3 µM), with substrate (3 µM) and FXa (E, 60 nM), and finally with substrate (3 µM), factor Xa (60 nM), and the FXa inhibitor gabexate mesylate (I, 15 µM) Data is binned by time (30 s) and fluorescence (5 RFU). (B) Droplet data were further reduced to yield three histograms, to visualize the cumulative separation in assay signals. Assay quality, Z′, is indicated.

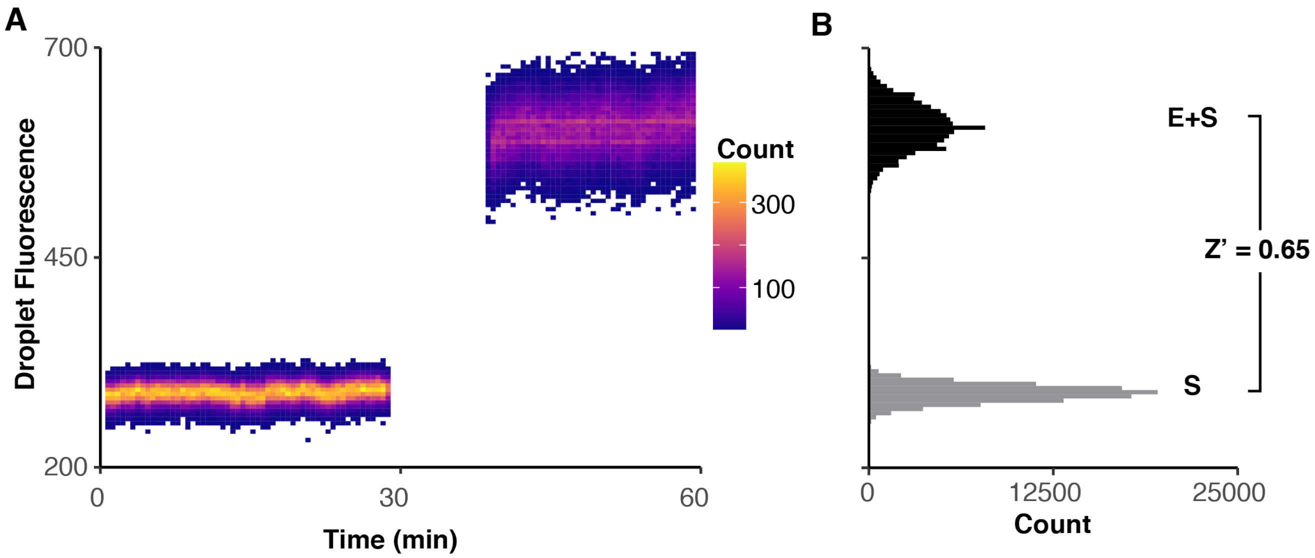

Figure S3. Droplet-scale ATX activity assay validation. Droplets were generated with fluorogenic FS-3 substrate only (S, 5 µM), and with substrate (5 µM) and ATX (E, 50 nM). Data were binned by time (30 s) and fluorescence (5 RFU). (B) Further data reduction yielded two histograms (binwidth = 5) to visualize the cumulative separation in assay signal. Assay quality, Z′, is indicated.

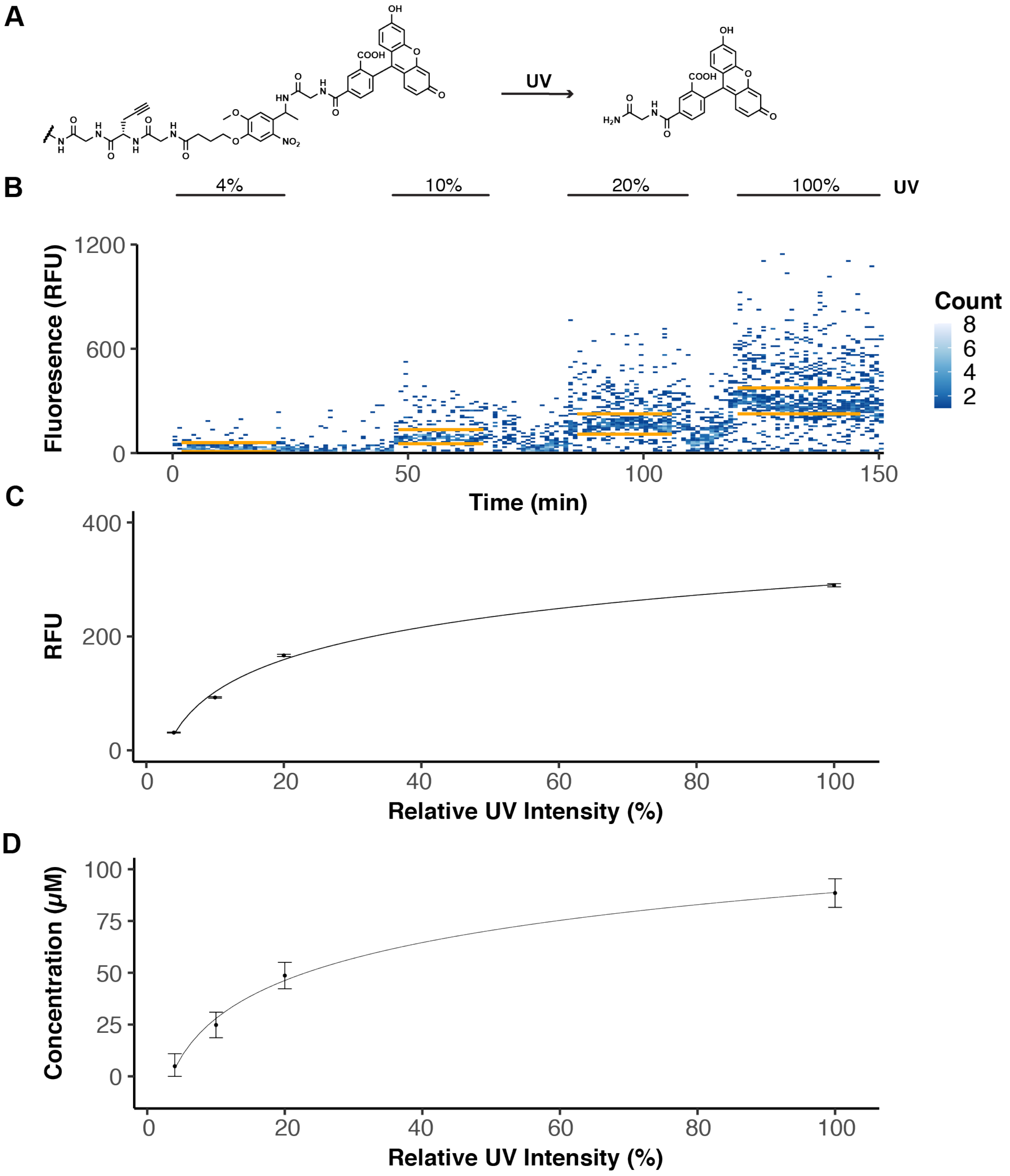

Figure S4. Quantification of fluorescein-glycine NH_2 photocleaved from PC-Gly-FAM beads into droplets. **(A)** The chemical structure of the linker attached to PC-Gly-FAM beads is depicted. Beads are encapsulated in droplets, and fluorescein-glycine NH_2 (Gly-FAM) is liberated in solution following UV exposure. **(B)** Gly-FAM signal from bead-containing droplets was detected after irradiating droplets with the indicated UV intensities. Singly-beaded droplets at each dose are indicated by orange bars. **(C)** The mean and standard error of droplet fluorescence from singly-beaded droplets was calculated and plotted for each UV dose. Data were fit to a logarithmic equation of the form $y = a \times \log(x)+k$. **(D)** Fluorescence units were converted to concentration by using a standard curve generated after measuring droplets containing 5-FAM at known concentrations.

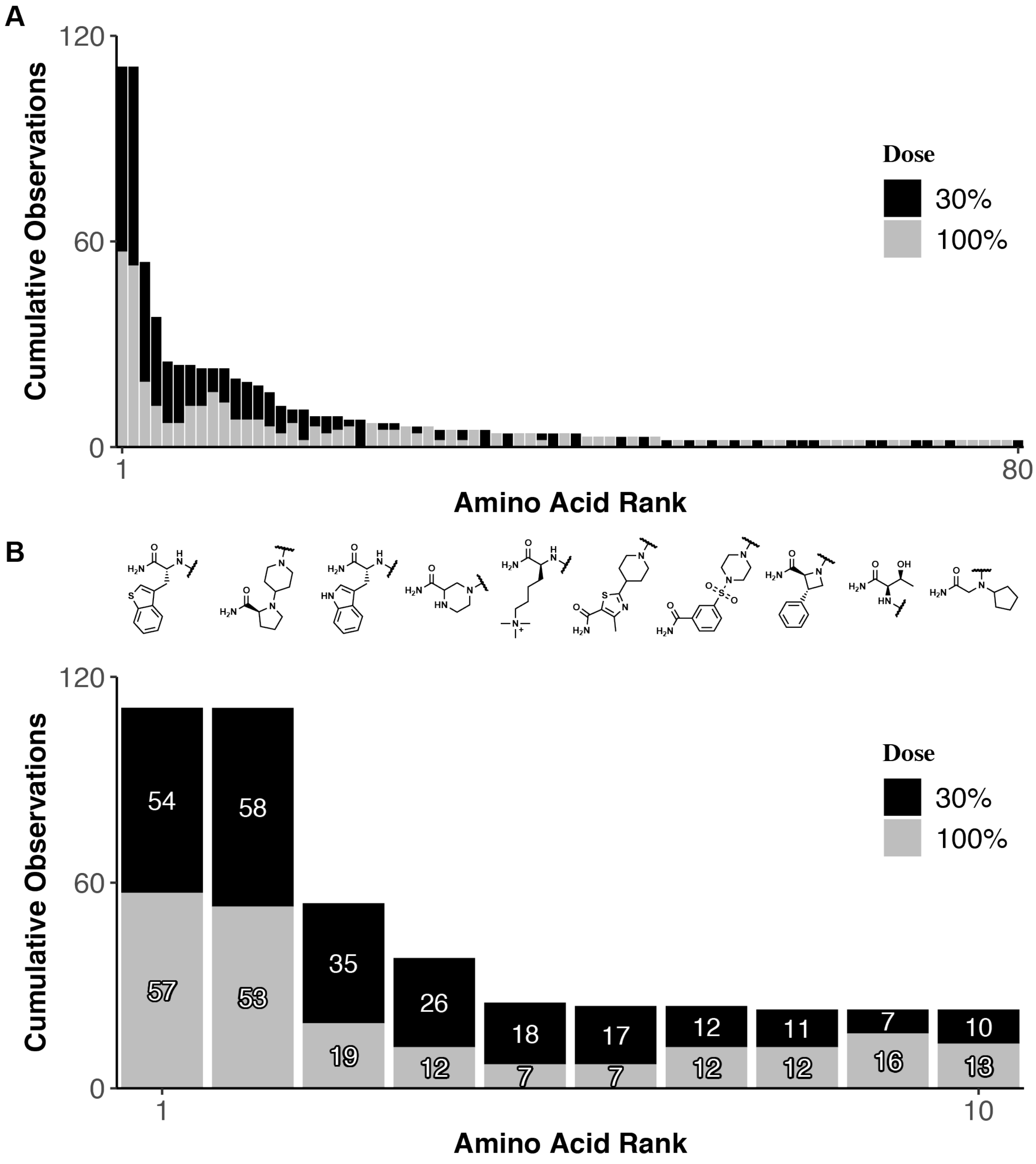

Figure S5. FXA amino acid hit BB enrichment. Hit beads were filtered to include only $k > 1$ hits. (A) Hits were aggregated by amino acid and UV intensity. (B) The top 10 hit structures are indicated.

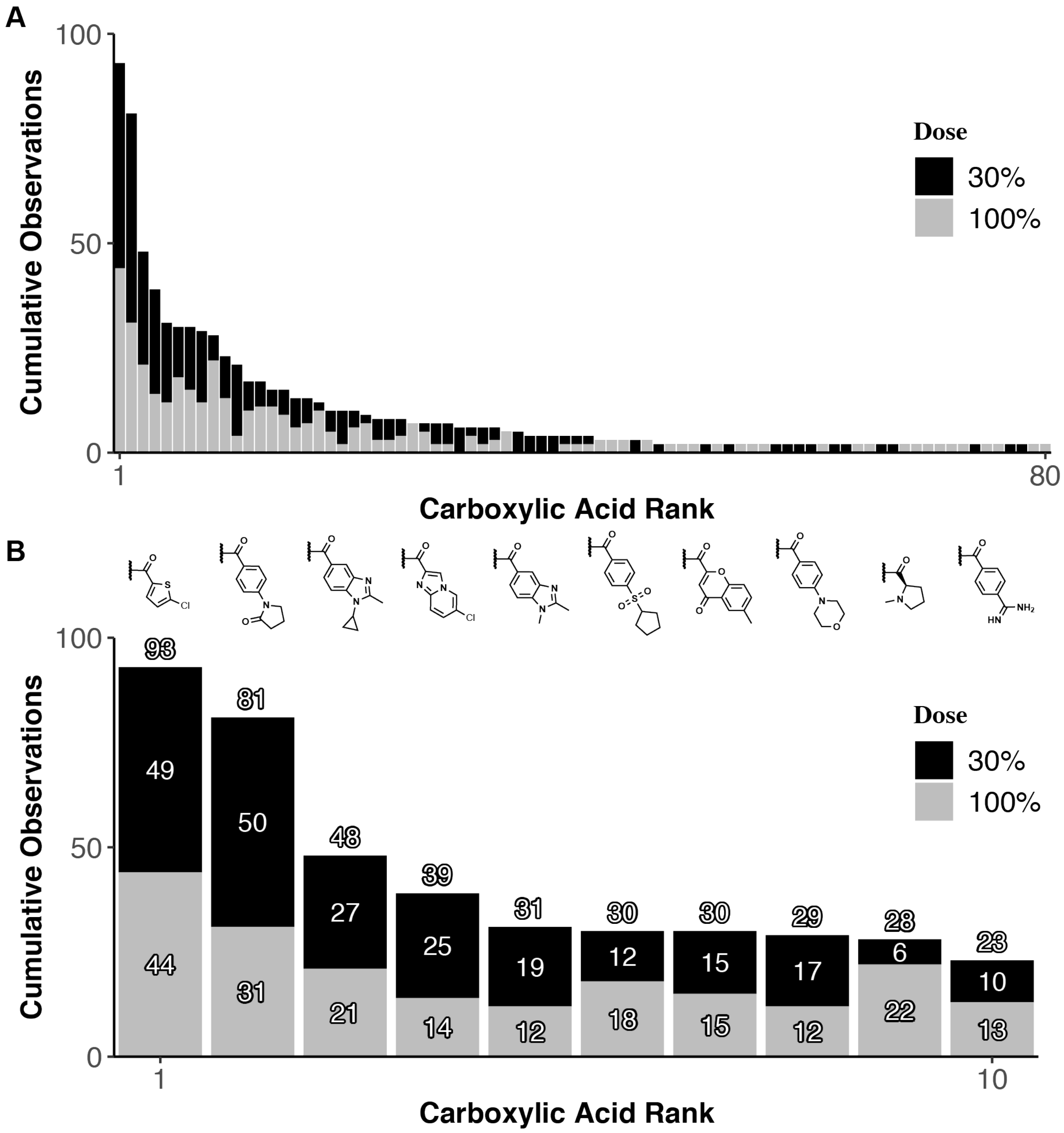

Figure S6. FXa carboxylic acid hit BB enrichment. Hit beads were filtered to include only $k > 1$ hits. Hits were aggregated by carboxylic acid and UV intensity. The top 10 hit structures are indicated.

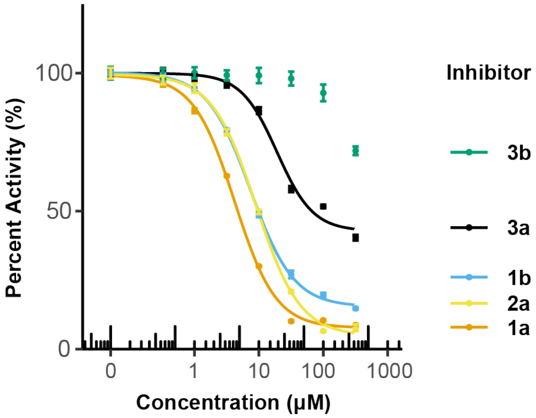

Figure S7. FXa screening hit validation. FXA was incubated with test compounds (30 min), then combined with fluorogenic substrate to assess extent of enzyme inhibition at each concentration. Error bars reflect standard error of the mean (n = 3).

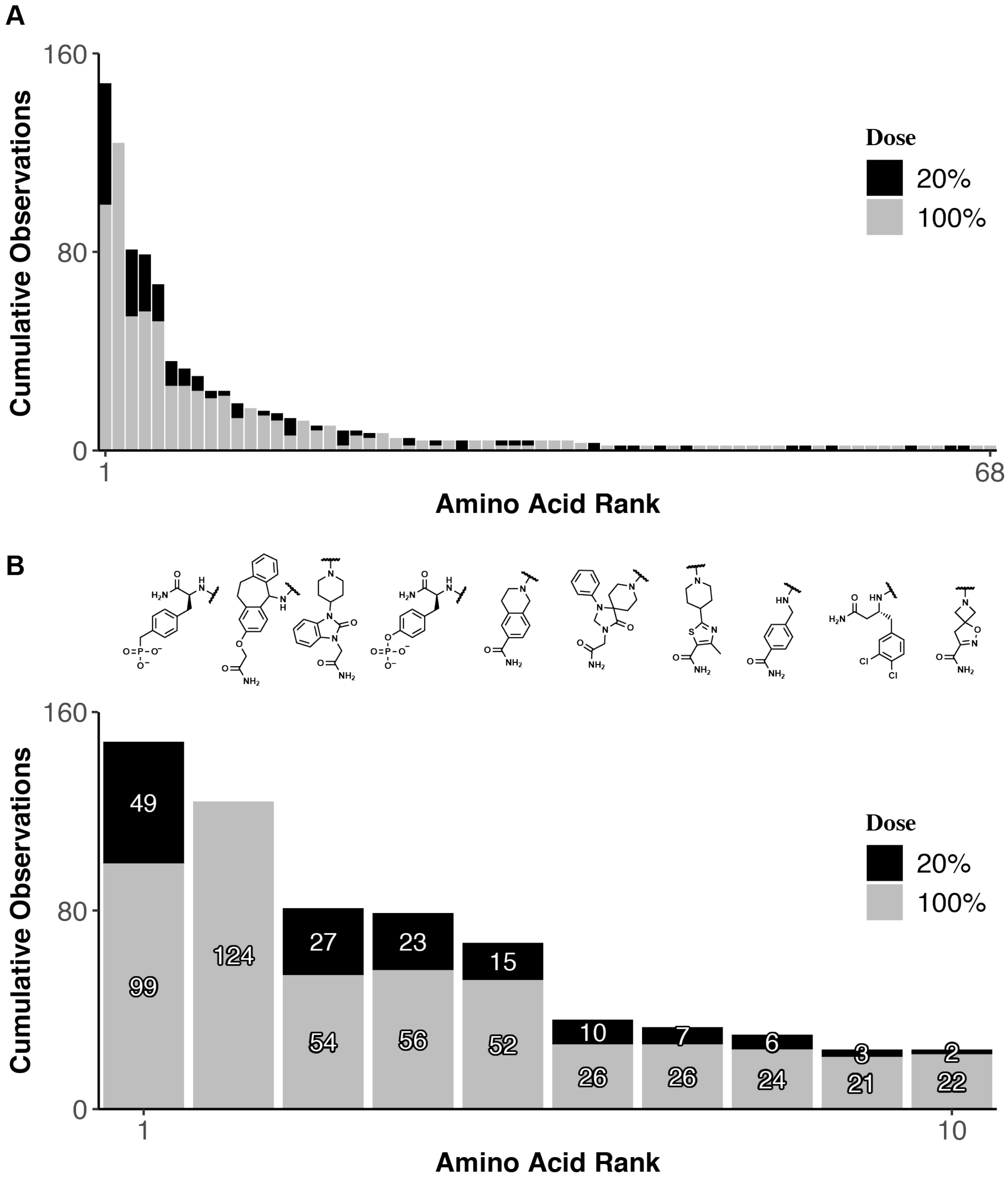

Figure S8. ATX amino acid hit BB enrichment. Hit beads were filtered to include only $k > 1$ hits. (A) Hits were aggregated by amino acid and UV intensity. (B) The top 10 hit structures are indicated.

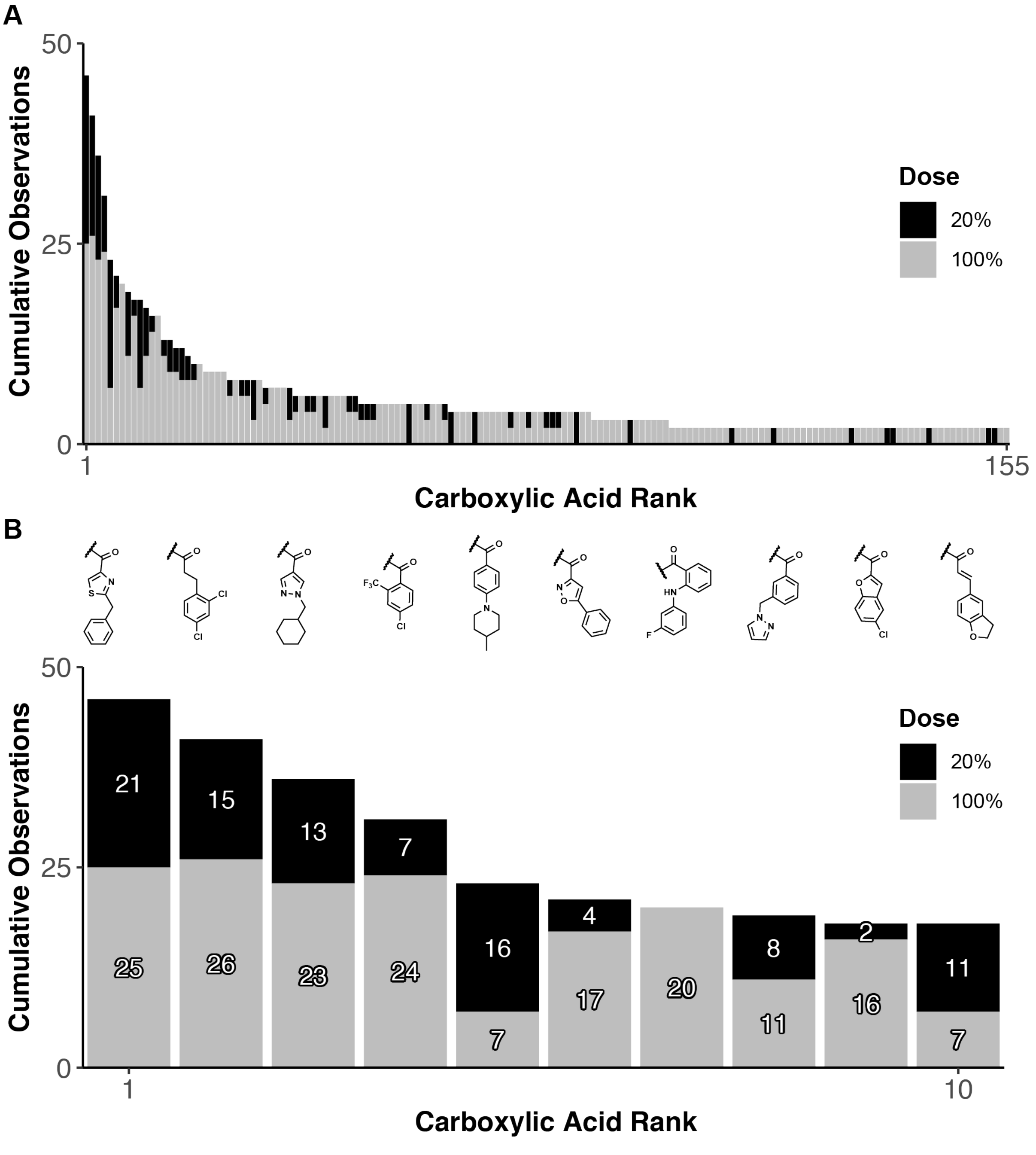

Figure S9. ATX carboxylic acid hit BB enrichment. Hit beads were filtered to include only $k > 1$ hits. (A) Hits were aggregated by carboxylic acid and UV intensity. (B) The top 10 hit structures are indicated.

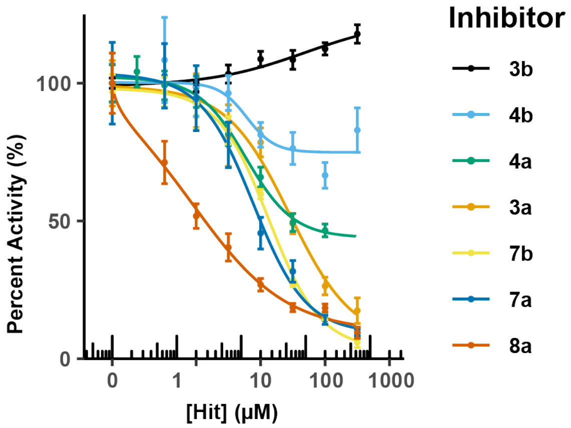

Figure S10. ATX was incubated with test compounds (30 min), then combined with fluorogenic substrate to measure ATX inhibition at each concentration. Error bars reflect standard error of the mean (n = 3).

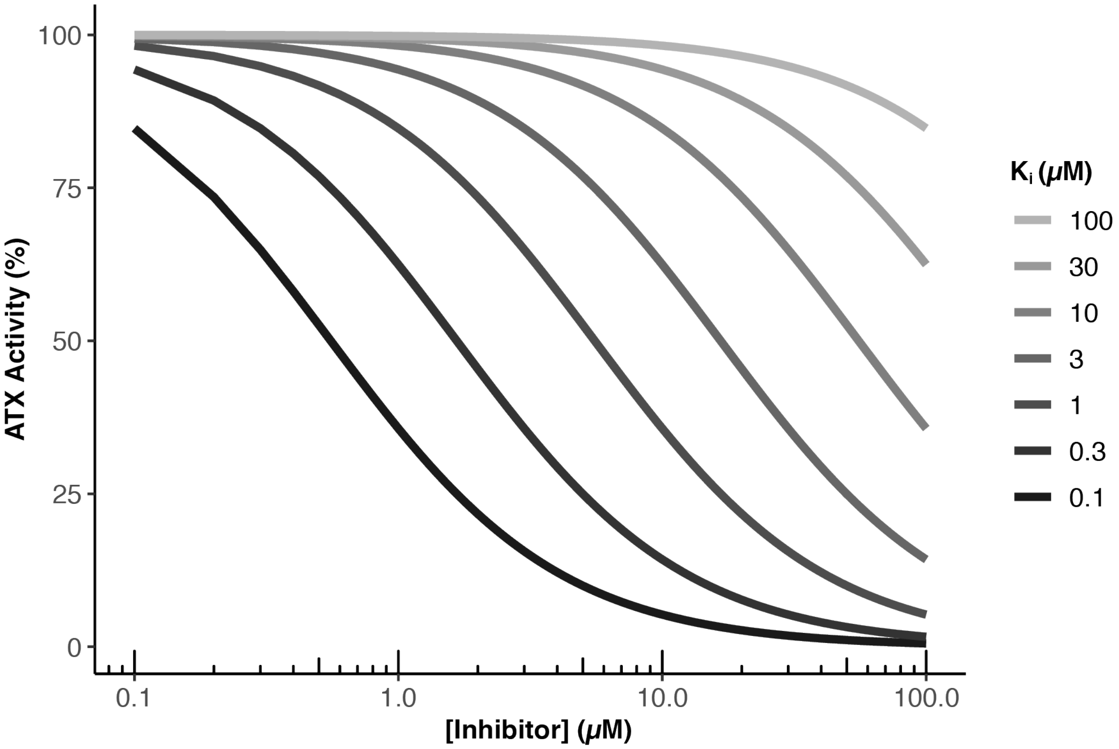

Figure S11. Modeling ATX competitive inhibition. ATX inhibition was modeled using the Michaelis-Menten steady-state approximation for competitive inhibition with a fixed K_M, fixed $[S]$, a fixed set of K_i values (0.1, 0.3, 1, 3, 10, 30, and 100 μM), and variable inhibitor concentration (0.1–100 μM).

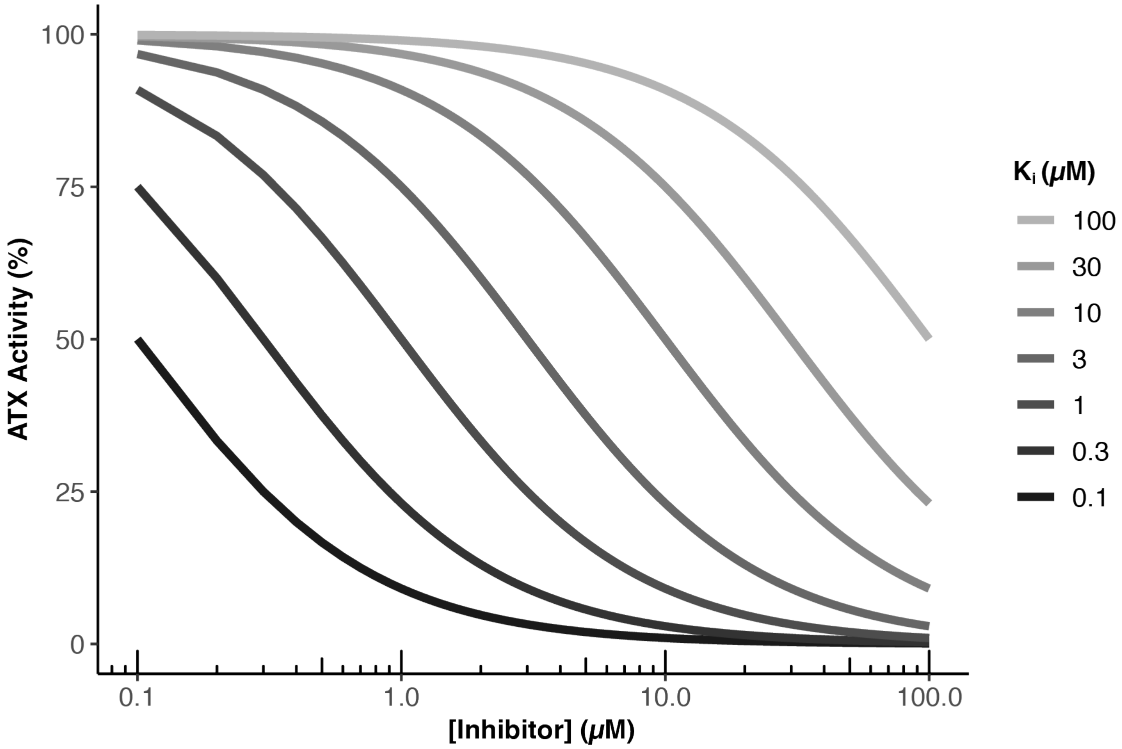

Figure S12. Modeling ATX noncompetitive inhibition. ATX noncompetitive inhibition was modeled using the Michaelis-Menten steady-state approximation for noncompetitive inhibition with a fixed K_M, fixed substrate concentration, a fixed set of K_i values (0.1, 0.3, 1, 3, 10, 30, and 100 μM), and variable inhibitor concentration (0.1-100 μM).

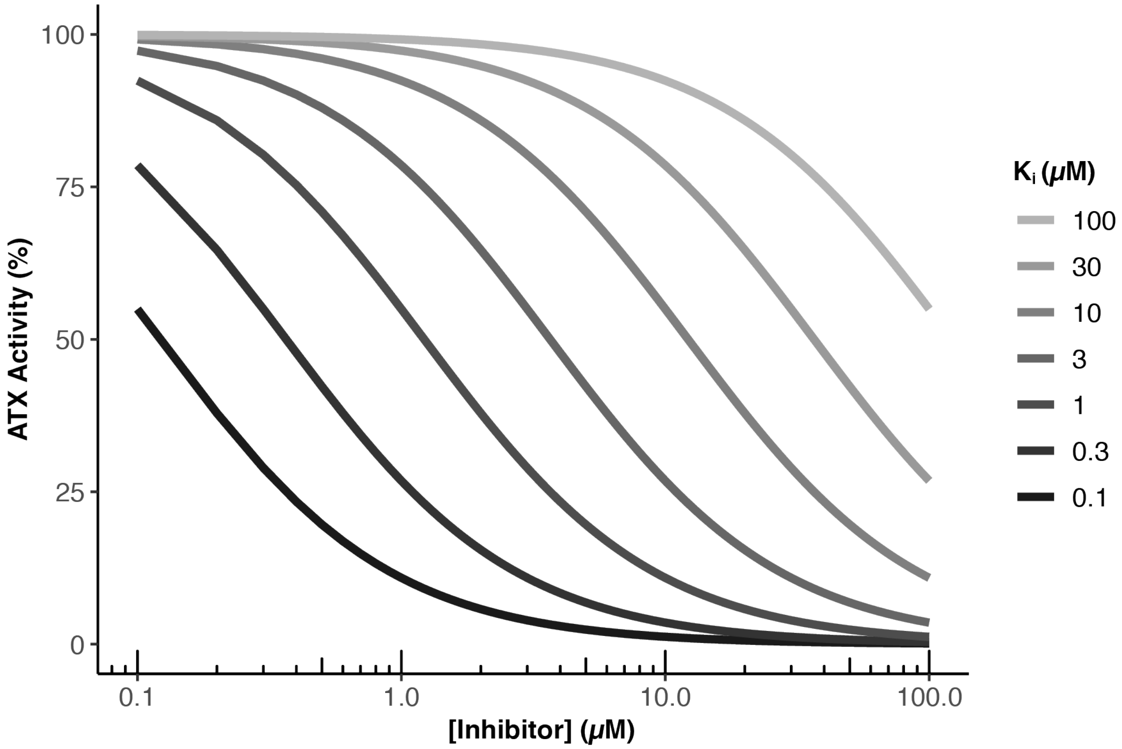

Figure S13. Modeling ATX unncompetitive inhibition. ATX unncompetitive inhibition was modeled using the Michaelis-Menten steady-state approximation for unncompetitive inhibition with a fixed K_M, fixed substrate concentration, a fixed set of K_i values (0.1, 0.3, 1, 3, 10, 30, and 100 µM), and variable inhibitor concentration (0.1-100 µM).

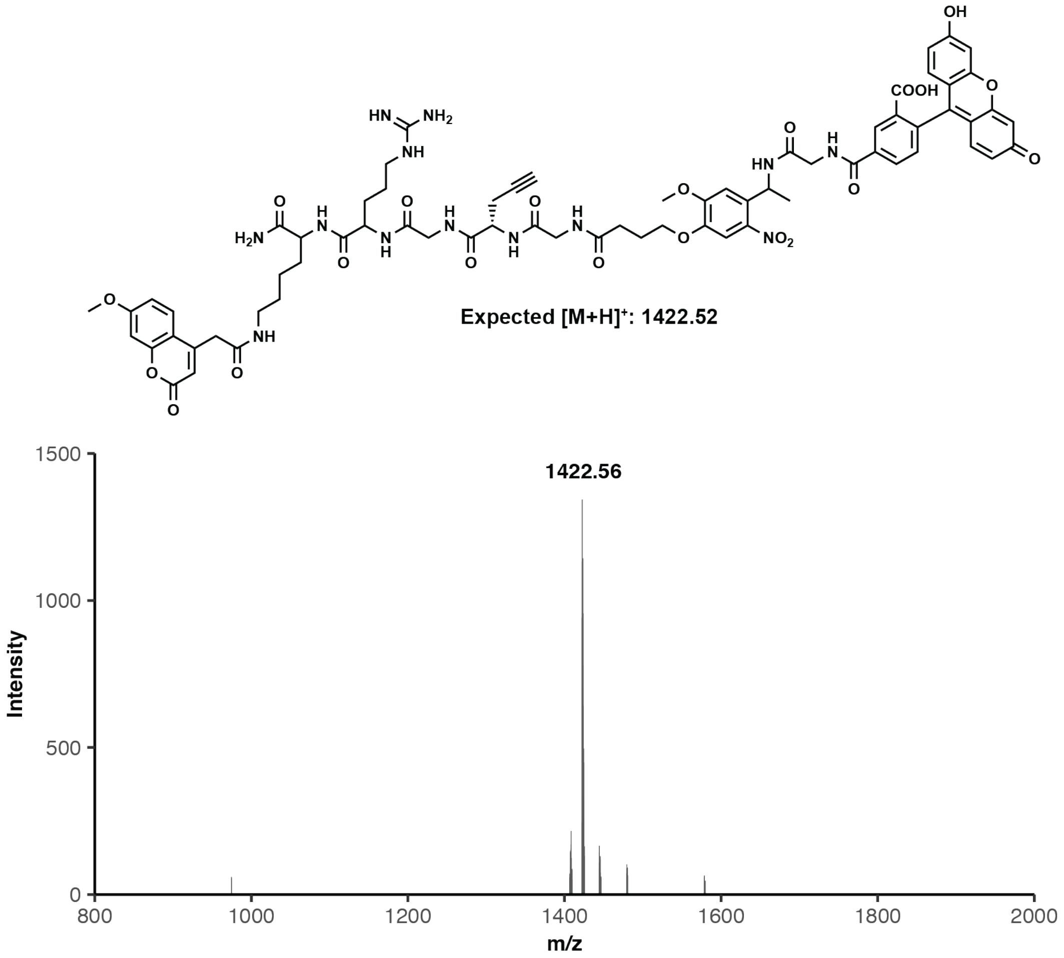

Figure S14. Characterization of PC-Gly-FAM. Compound was cleaved from QC-resin and analyzed by MALDI-TOF MS. The mass spectrum of the product contained a peak with the observed ion (1422.56 m/z) corresponding to the expected $[M+H]^+$ (1422.52 Da). Matrix peaks below 800 m/z are omitted.

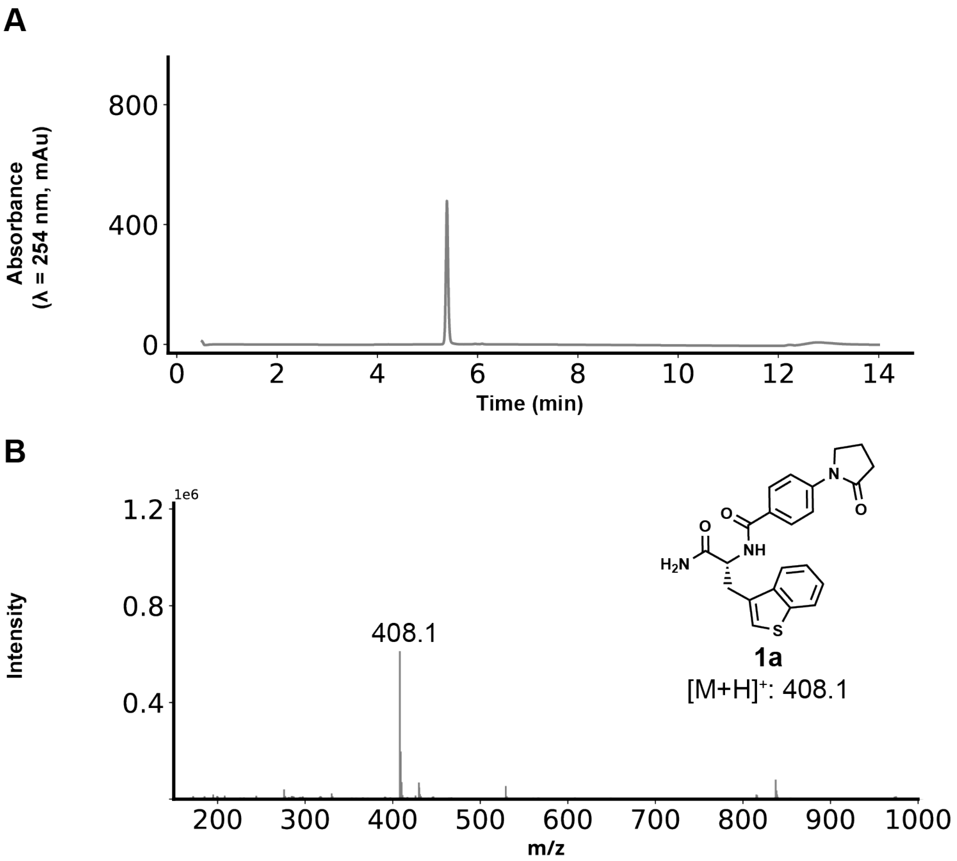

Figure S15. Characterization of **1a**. (A) The major peak of the HPLC chromatogram (λ = 254 nm) contained **1a**. (B) The mass spectrum of the product contained a peak with the observed ion (408.1 m/z) corresponding to the expected $[M+H]^+$ (408.1 Da).

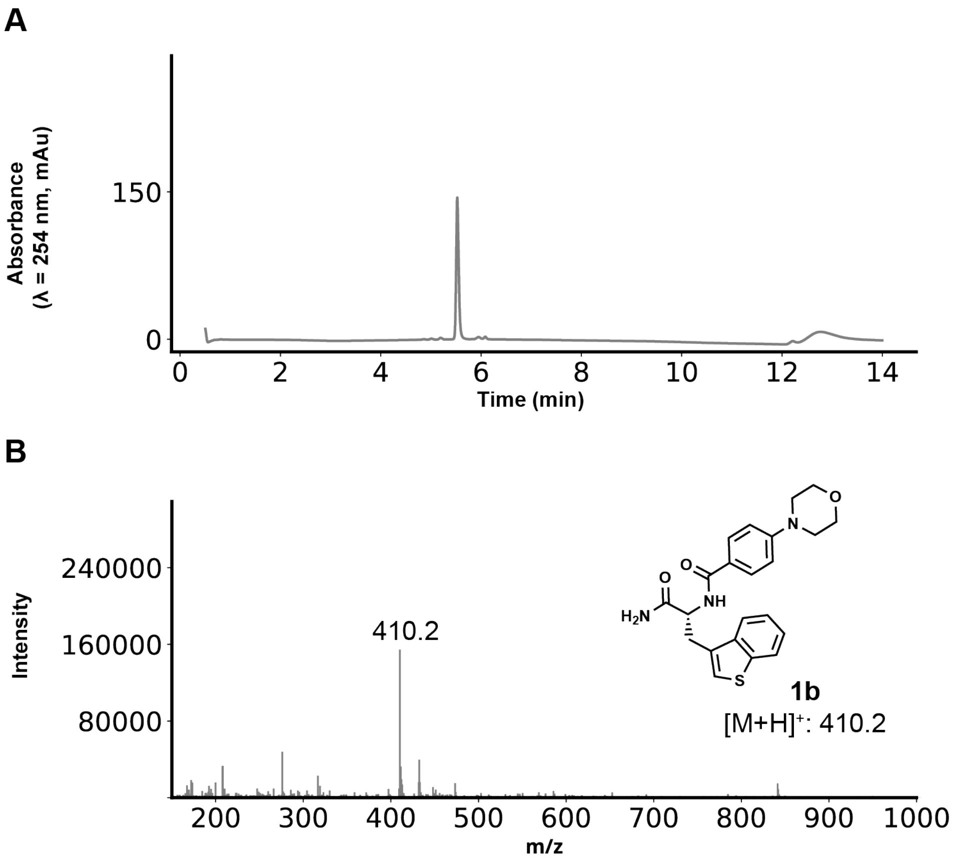

Figure S16. Characterization of **1b**. (A) The major peak of the HPLC chromatogram (λ = 254 nm) contained **1b**. (B) The mass spectrum of the product contained a peak with the observed ion (410.2 m/z) corresponding to the expected $[M+H]^+$ (410.2 Da).

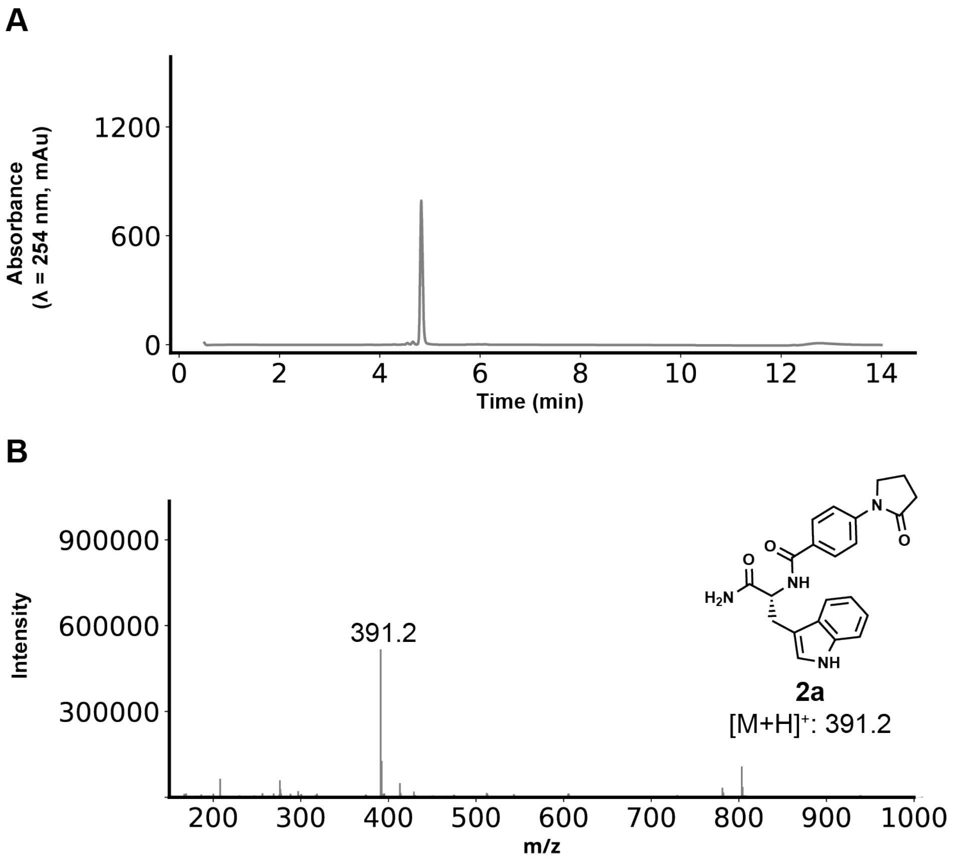

Figure S17. Characterization of **2a**. (A) The major peak of the HPLC chromatogram (λ = 254 nm) contained **2a**. (B) The mass spectrum of the product contained a peak with the observed ion (391.2 m/z) corresponding to the expected $[M+H]^+$ (391.2 Da).

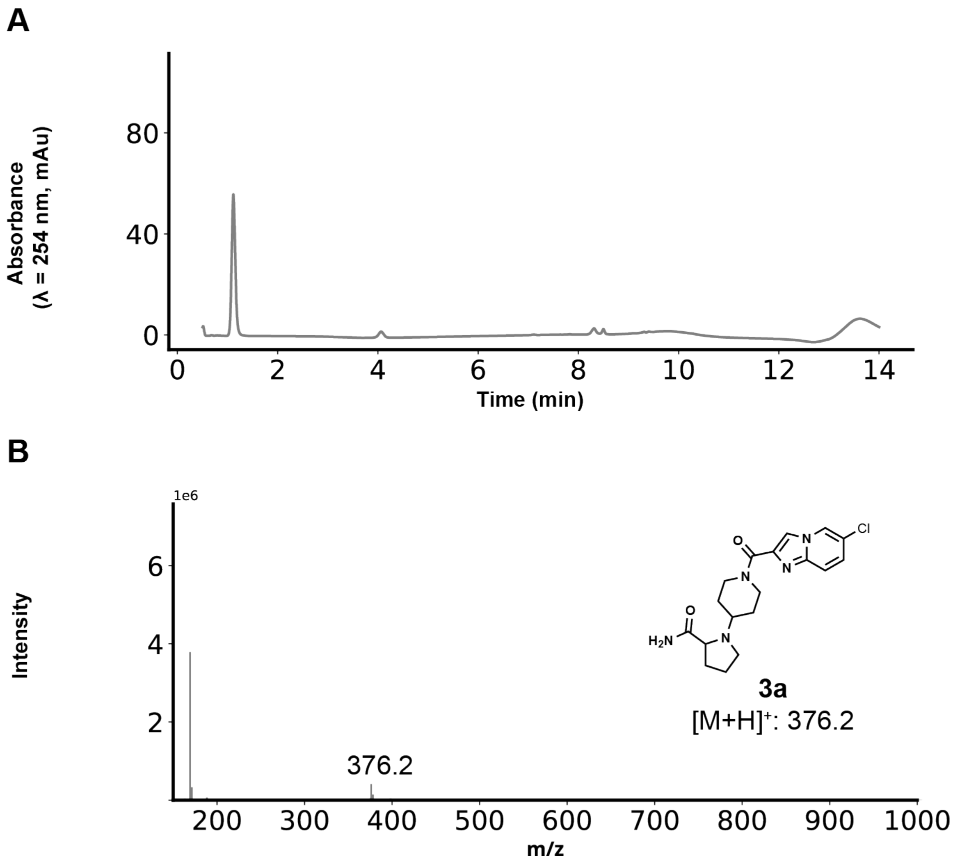

Figure S18. Characterization of **3a**. (A) The major peak of the HPLC chromatogram (λ = 254 nm) contained **3a**. (B) The mass spectrum of the product contained a peak with the observed ion (376.2 m/z) corresponding to the expected $[M+H]^+$ (376.2 Da).

A

B

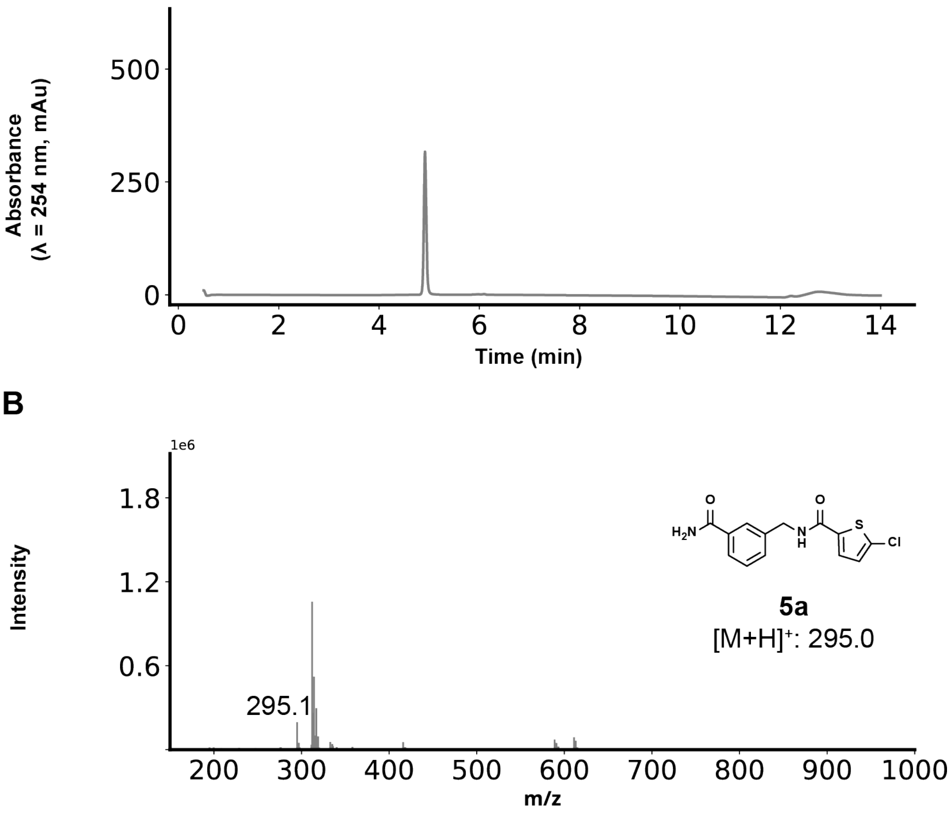

Figure S19. Characterization of **5a**. (A) The major peak of the HPLC chromatogram (λ = 254 nm) contained **5a**. (B) The mass spectrum of the product contained a peak with the observed ion (295.1 m/z) corresponding to the expected $[M+H]^+$ (295.0 Da).

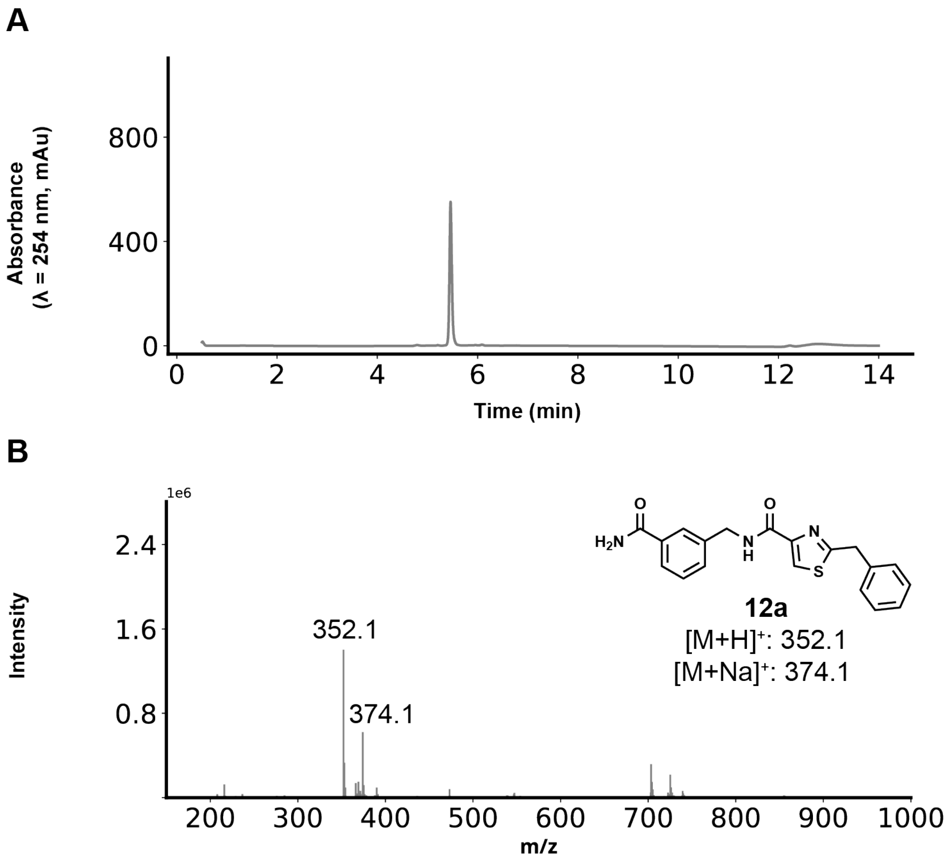

Figure S20. Characterization of **12a**. (A) The major peak of the HPLC chromatogram (λ = 254 nm) contained **12a**. (B) The mass spectrum of the product contained a peak with the observed ion (352.1 m/z) corresponding to the expected $[M+H]^+$ (352.1 Da).

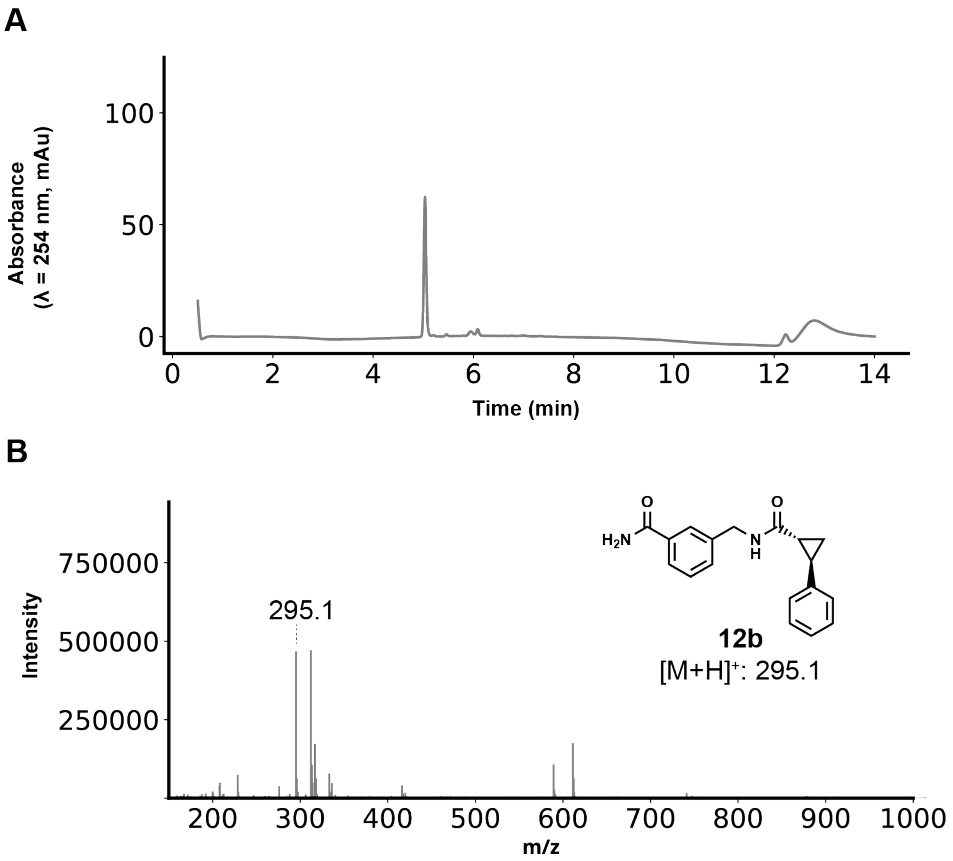

Figure S21. Characterization of **12b**. (A) The major peak of the HPLC chromatogram (λ = 254 nm) contained **12b**. (B) The mass spectrum of the product contained a peak with the observed ion (295.1 m/z) corresponding to the expected $[M+H]^+$ (295.1 Da).

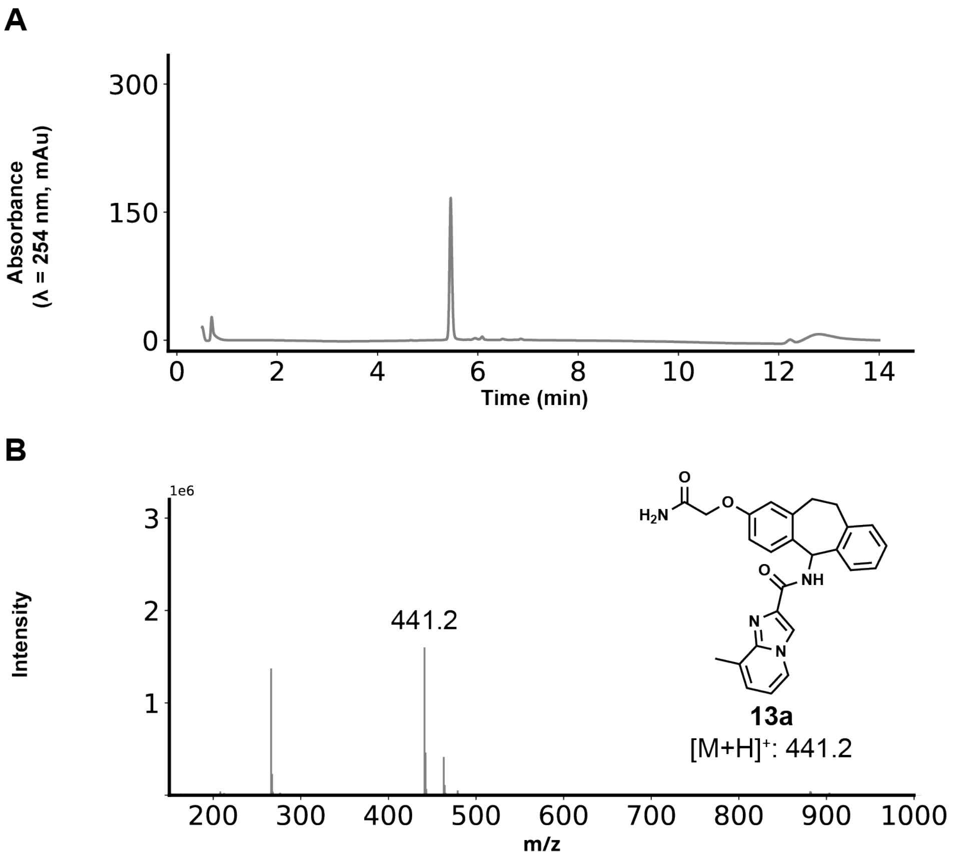

Figure S22. Characterization of **13a**. (A) The major peak of the HPLC chromatogram (λ = 254 nm) contained **13a**. (B) The mass spectrum of the product contained a peak with the observed ion (441.2 m/z) corresponding to the expected $[M+H]^+$ (441.2 Da).

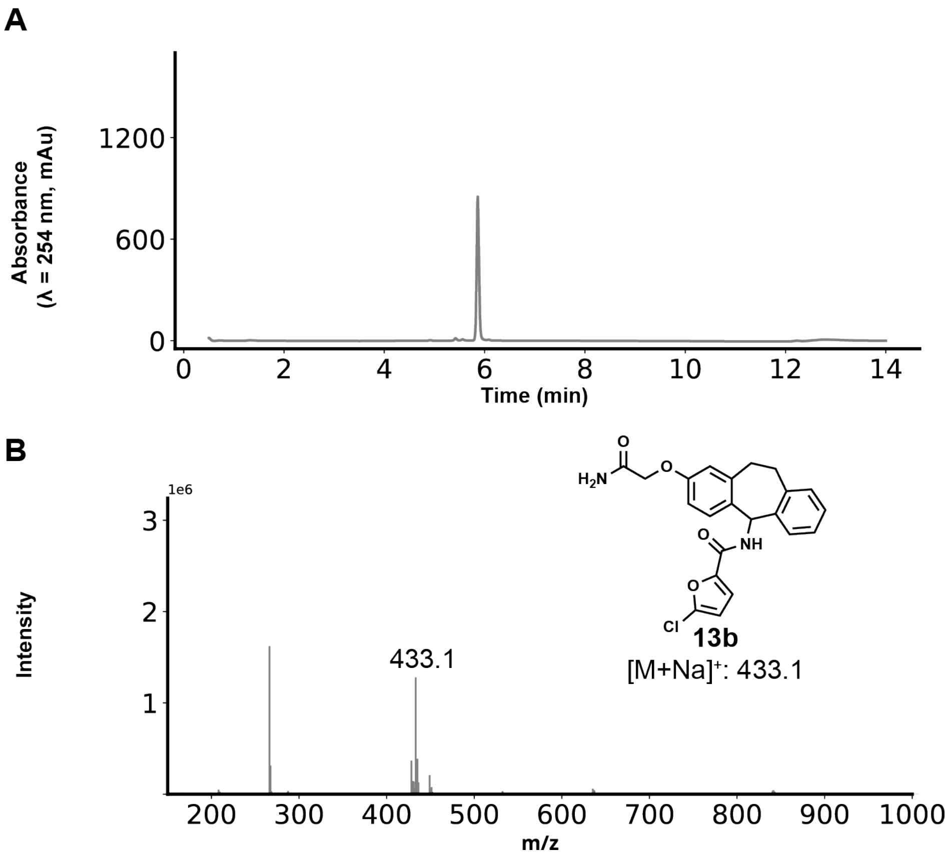

Figure S23. Characterization of **13b**. (A) The major peak of the HPLC chromatogram (λ = 254 nm) contained **13b**. (B) The mass spectrum of the product contained a peak with the observed ion (411.1 m/z) corresponding to the expected $[M+H]^+$ (411.1 Da).

A

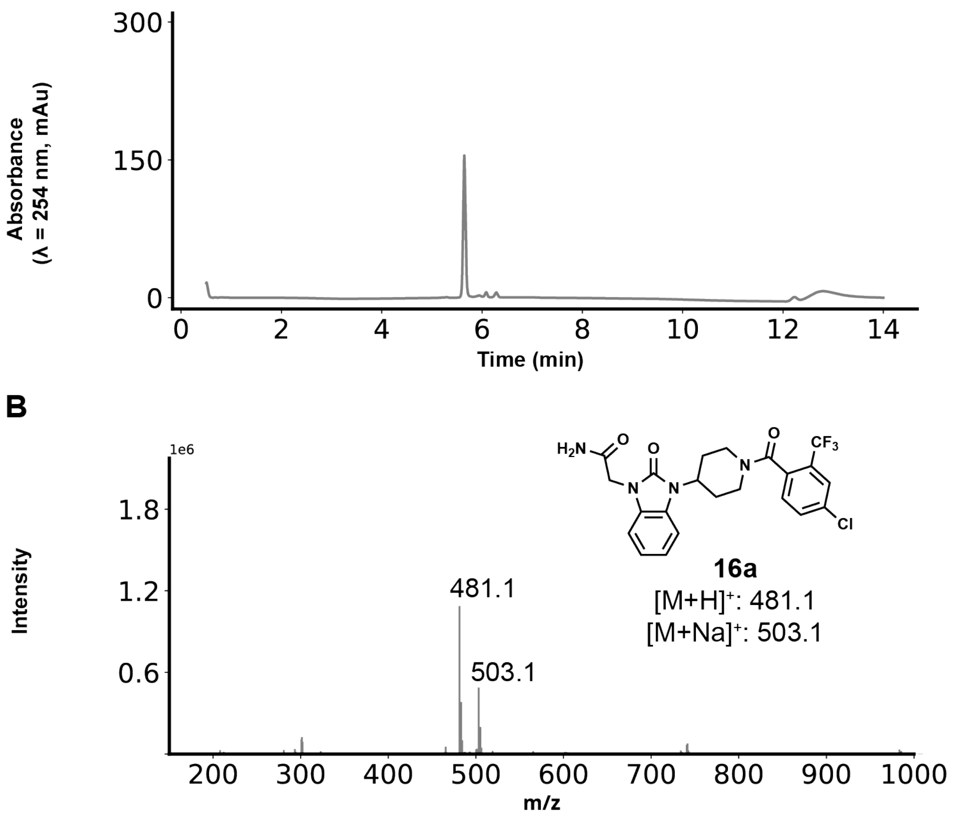

Figure S24. Characterization of **16a**. (A) The major peak of the HPLC chromatogram (λ = 254 nm) contained **16a**. (B) The mass spectrum of the product contained a peak with the observed ion (481.1 m/z) corresponding to the expected $[M+H]^+$ (481.1 Da).

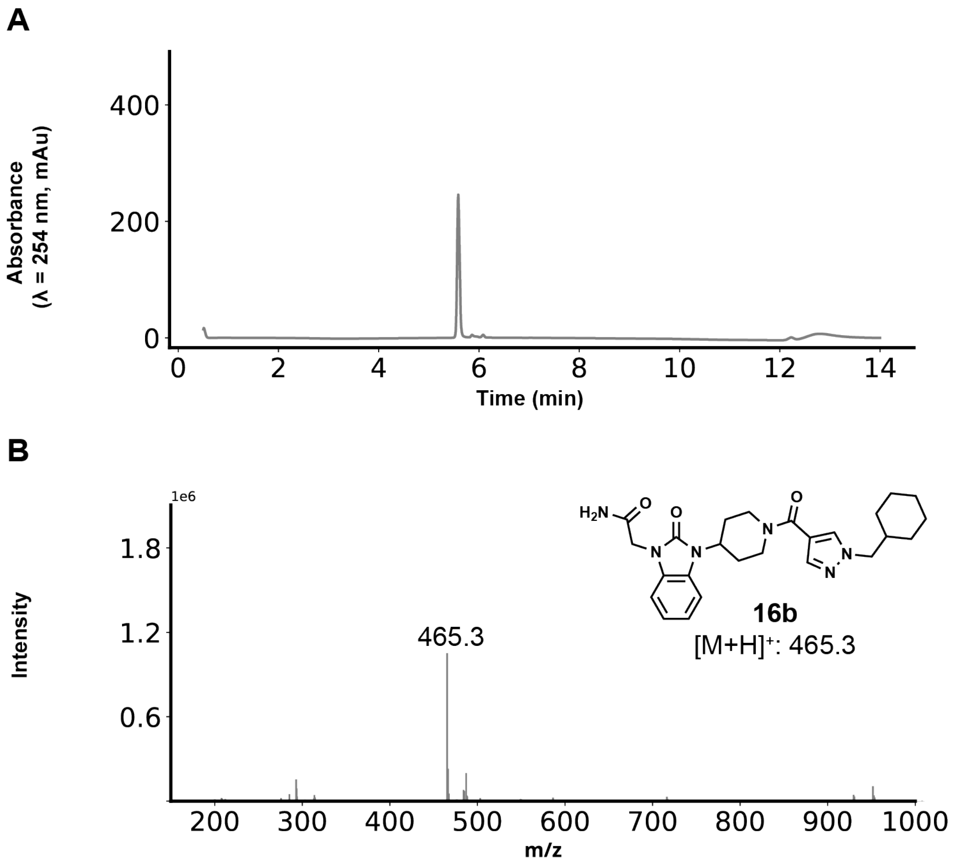

Figure S25. Characterization of **16b**. (A) The major peak of the HPLC chromatogram (λ = 254 nm) contained **16b**. (B) The mass spectrum of the product contained a peak with the observed ion (465.2 m/z) corresponding to the expected $[M+H]^+$ (465.3 Da).

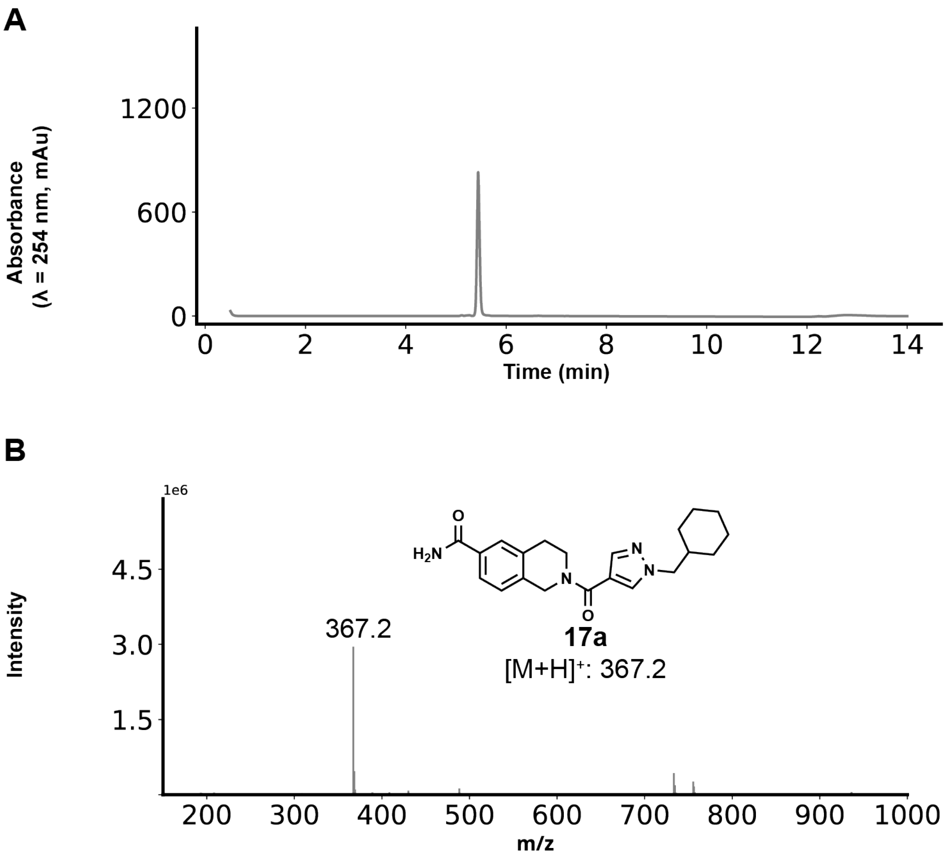

Figure S26. Characterization of **17a**. (A) The major peak of the HPLC chromatogram (λ = 254 nm) contained **17a**. (B) The mass spectrum of the product contained a peak with the observed ion (367.2 m/z) corresponding to the expected $[M+H]^+$ (367.1 Da).

Supporting Schemes

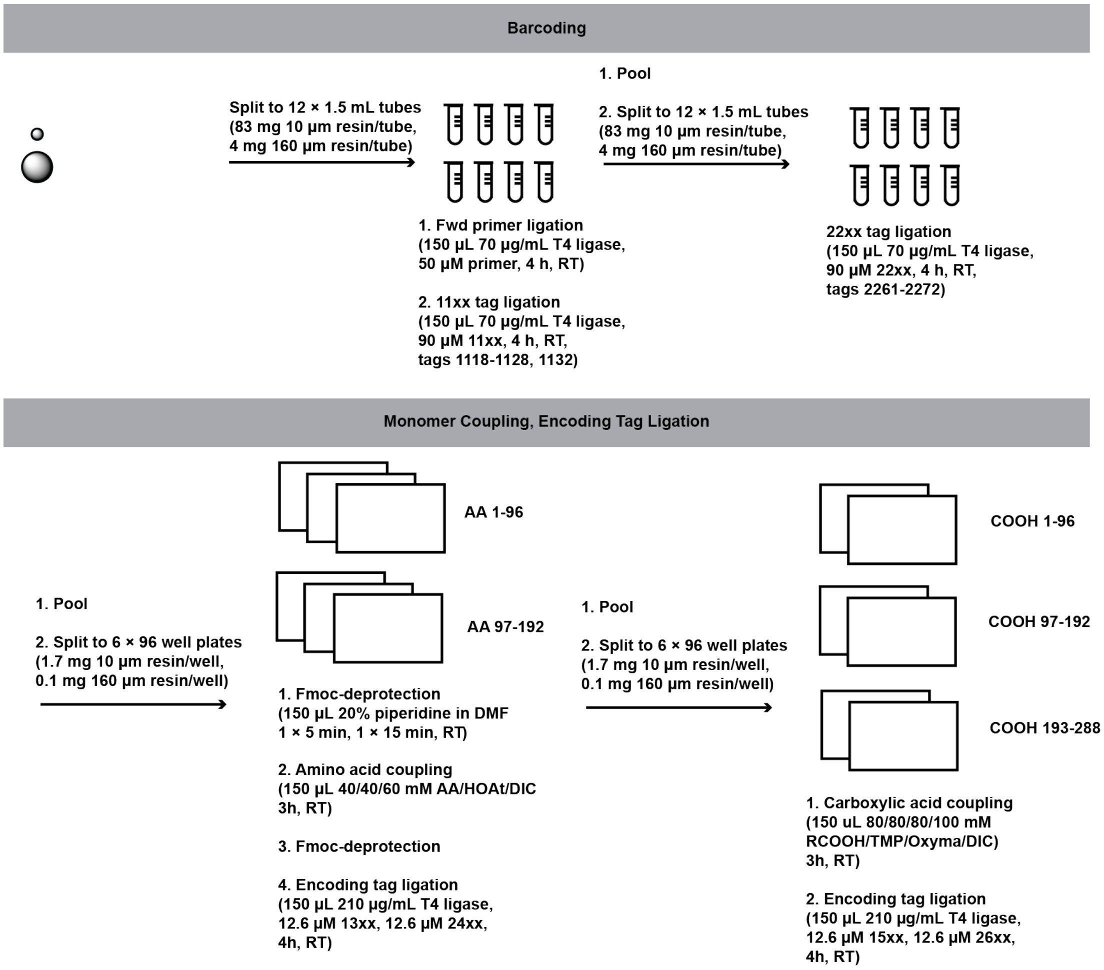

Scheme S1. DEL Combinatorial Synthesis.

References

(1) MacConnell, A. B.; McEnaney, P. J.; Cavett, V. J.; Paegel, B. M. DNA-Encoded Solid-Phase Synthesis: Encoding Language Design and Complex Oligomer Library Synthesis. *ACS Comb. Sci.* **2015**, *17*, 518–534.

(2) Mendes, K. R.; Malone, M. L.; Ndungu, J. M.; Suponitsky-Kroyter, I.; Cavett, V. J.; McEnaney, P. J.; MacConnell, A. B.; Doran, T. D.; Ronacher, K.; Stanley, K.; Utset, O.; Walzl, G.; Paegel, B. M.; Kodadek, T. High-throughput Identification of DNA-encoded IgG Ligands that Distinguish Active and Latent Mycobacterium Tuberculosis Infections. *ACS Chem. Biol.* **2017**, *12*, 234–243.

(3) MacConnell, A. B.; Paegel, B. M. Poisson Statistics of Combinatorial Library Sampling Predict False Discovery Rates of Screening. *ACS Comb. Sci.* **2017**, *19*, 524–532.

(4) Cochrane, W. G.; Malone, M. L.; Dang, V. Q.; Cavett, V. J.; Satz, A. L.; Paegel, B. M. Activity-Based DNA-Encoded Library Screening. *ACS Comb. Sci.* **2019**, *21*, 425–435.

(5) Hackler, A. L.; FitzGerald, F. G.; Dang, V. Q.; Satz, A. L.; Paegel, B. M. Off-DNA DNA-Encoded Library Affinity Screening. *ACS Comb. Sci.* **2020**, *22*, 25–34.

(6) Duffy, D. C.; McDonald, J. C.; Schueller, O. J. A.; Whitesides, G. M. Rapid Prototyping of Microfluidic Systems in Poly(dimethylsiloxane). *Anal. Chem.* **1998**, *70*, 4974–4984.

(7) Price, A. K.; MacConnell, A. B.; Paegel, B. M. hνSABR: Photochemical Dose-Response Bead Screening in Droplets. *Anal. Chem.* **2016**, *88*, 2904–2911.

(8) Cochrane, W. G.; Hackler, A. L.; Cavett, V. J.; Price, A. K.; Paegel, B. M. Integrated, Continuous Emulsion Creamer. *Anal. Chem.* **2017**, *89*, 13227–13234.

(9) Ferguson, C. G.; Bigman, C. S.; Richardson, R. D.; Van Meeteren, L. A.; Moolenaar, W. H.; Prestwich, G. D. Fluorogenic Phospholipid Substrate To Detect Lysophospholipase D/Autotaxin Activity. *Org. Lett.* **2006**, *8*, 2023–2026.

(10) Saunders, L. P.; Cao, W.; Chang, W. C.; Albright, R. A.; Braddock, D. T.; De La Cruz, E. M. Kinetic Analysis of Autotaxin Reveals Substrate-Specific Catalytic Pathways and a Mechanism for Lysophosphatidic Acid Distribution. *J. Biol. Chem.* **2011**, *286*, 30130–30141.

(11) Chou, Y.-L. L.; Davey, D. D.; Eagen, K. A.; Griedel, B. D.; Karanjawala, R.; Phillips, G. B.; Sacchi, K. L.; Shaw, K. J.; Wu, S. C.; Lentz, D.; Liang, A. M.; Trinh, L.; Morrissey, M. M.; Kochanny, M. J. Structure-Activity Relationships of Substituted Benzothiophene-Anthranilamide Factor Xa Inhibitors. *Bioorganic Med. Chem. Lett.* **2003**, *13*, 507–511.

(12) Fujimoto, T.; Imaeda, Y.; Konishi, N.; Hiroe, K.; Kawamura, M.; Textor, G. P.; Aertgeerts, K.; Kubo, K. Discovery of a Tetrahydropyrimidin-2(1 H)-One Derivative (TAK-442) as a Potent, Selective, and Orally Active Factor Xa Inhibitor. *J. Med. Chem.* **2010**, *53*, 3517–3531.

(13) Haginoya, N.; Kobayashi, S.; Komoriya, S.; Hirokawa, Y.; Furugori, T.; Nagahara, T. Orally Active Factor Xa Inhibitors: 4,5, 6,7-tetrahydrothiazolo[5,4-c] Pyridine Derivatives. *Bioorganic Med. Chem. Lett.* **2004**, *14*, 2935–2939.

(14) Roehrig, S.; Straub, A.; Pohlmann, J.; Lampe, T.; Pernerstorfer, J.; Schlemmer, K. H.; Reinemer, P.; Perzborn, E. Discovery of the Novel Antithrombotic Agent 5-Chloro-N-({(5S)-2-Oxo-3-[4-(3-Oxomorpholin-4-Yl)phenyl]-1,3-Oxazolidin-5-Yl}methyl)thiophene-2-Carboxamide (BAY 59–7939): An Oral, Direct Factor Xa Inhibitor. *J. Med. Chem.* **2005**, *48*, 5900–5908.

(15) Pinto, D. J.; Orwat, M. J.; Koch, S.; Rossi, K. A.; Alexander, R. S.; Smallwood, A.; Wong, P. C.; Rendina, A. R.; Luettgen, J. M.; Knabb, R. M.; He, K.; Xin, B.; Wexler, R. R.; Lam, P. Y. Discovery of 1-(4-methoxyphenyl)-7-oxo-6-(4-(2-oxopiperidin-1-yl)phenyl)-4, 5,6,7-tetrahydro-1H-pyrazolo[3,4-c]pyridine-3-carboxamide (Apixa-

ban, BMS-562247), a Highly Potent, Selective, Efficacious, and Orally Bioavailable Inhibitor of Blood Coagulation F. *J. Med. Chem.* **2007**, *50*, 5339–5356.

(16) Zhao, Z. S.; Arnaiz, D. O.; Griedel, B.; Sakata, S.; Dallas, J. L.; Whitlow, M.; Trinh, L.; Post, J.; Liang, A.; Morrissey, M. M.; Shaw, K. J. Design, Synthesis, and In Vitro Biological Activity of Benzimidazole Based Factor Xa Inhibitors. *Bioorg. Med. Chem. Lett.* **2000**, *10*, 963–966.
